# Chiral multifunctional thiourea-phosphine catalyzed asymmetric [3 + 2] annulation of Morita–Baylis–Hillman carbonates with maleimides

**DOI:** 10.3762/bjoc.8.121

**Published:** 2012-07-16

**Authors:** Hong-Ping Deng, De Wang, Yin Wei, Min Shi

**Affiliations:** 1 State Key Laboratory of Organometallic Chemistry, Shanghai Institute of Organic Chemistry, Chinese Academy of Sciences, 354 Fenglin Road, Shanghai 200032, People’s Republic of China; 2 Key Laboratory for Advanced Materials and Institute of Fine Chemicals, School of Chemistry & Molecular Engineering, East China University of Science and Technology, and 130 MeiLong Road, Shanghai 200237, People’s Republic of China

**Keywords:** asymmetric [3 + 2] annulation, maleimides, Morita–Baylis–Hillman carbonates, multifunctional thiourea-phosphine, organocatalysis

## Abstract

We have developed a multifunctional thiourea-phosphine catalyzed asymmetric [3 + 2] annulation of Morita–Baylis–Hillman (MBH) carbonates with maleimides, which can efficiently construct functionalized cyclopentenes bearing three contiguous stereocenters in moderate to excellent yields and excellent diastereo- and enantioselectivities. A plausible mechanism has been also proposed on the basis of control experiments and previous literature.

## Introduction

**Scheme 1:**
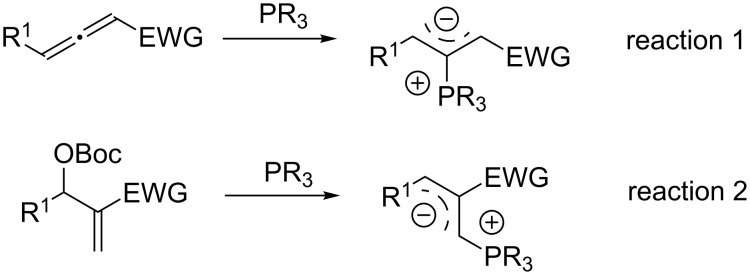
Paths to the formation of 1,3-dipolar synthons by using a catalytic amount of phosphines.

Highly functionalized cyclopentene derivatives are important subunits in a variety of biologically active molecules and have attracted the broad attention of synthetic and pharmaceutical chemists [1–2]. Among numerous synthetic approaches, phosphine-mediated [3 + 2] annulation of electron-deficient olefins is an efficient method to construct this interesting structural motif [3–10]. According to the pioneering work of Lu [11–20], all phosphine-mediated [3 + 2] annulations proceeded through an important intermediate “1,3-dipolar synthon”. The formation of a 1,3-dipolar synthon by using a catalytic amount of phosphines have been directed toward the following two paths: phosphines attack the middle carbon atom of allenes to produce the 1,3-dipolar synthon (Scheme 1, reaction 1), and phosphines add to the *β*-position of MBH carbonate to remove carbon dioxide and *tert*-butanol, affording the 1,3-dipolar synthon (Scheme 1, reaction 2). Concerning the asymmetric [3 + 2] annulation catalyzed by chiral phosphines, Zhang and co-workers first reported the asymmetric [3 + 2] annulation of allenoates with acrylates catalyzed by a bicyclic chiral phosphine in 1997 [21]. Moreover, Fu [22–24], Marinetti [25–28], Lu [29–31] and other researchers [32–36] have also developed asymmetric [3 + 2] annulations of allenoates to give the corresponding cyclopentene derivatives in good yields with excellent enantioselectivities [37].

On the other hand, some examples of phosphine-catalyzed [3 + 2] annulation of MBH carbonates with electron-deficient alkenes have been reported in the literature recently. Lu and co-workers first disclosed the formation of 1,3-dipoles through phosphine-catalyzed MBH carbonates under mild conditions, which can efficiently react with various electron-deficient olefins to afford the desired cyclic products in good yields along with high regioselectivities, through intra- and intermolecular [3 + *n*] annulations [38–47]. To the best of our knowledge, there is little in the literature regarding the asymmetric version of this reaction. In 2010, Tang and co-workers reported the first example of asymmetric intramolecular [3 + 2] annulations of MBH carbonates tethered with another electron-deficient olefin in the presence of spirobiindane-based chiral phosphines, giving the corresponding cycloadducts in good yields along with high ee values [48]. Barbas and co-workers first reported asymmetric intermolecular [3 + 2] cycloaddition of MBH carbonates with methyleneindolinones to afford the corresponding spirocyclopentaneoxindoles in good yields and high ee values in 2011 [49]. Moreover, Lu and co-workers have recently explored a series of thiourea-phosphine catalysts derived from L-threonine, which are effective catalysts in the [3 + 2] annulation of MBH carbonates with isatylidene malononitriles to give the desired products in high yields with high enantioselectivities [50]. Furthermore, our group recently also synthesized a series of L-phenylalanine-derived multifunctional thiourea-phosphine catalysts and used them in the [3 + 2] annulation of MBH carbonates with trifluoroethylidenemalonates to give the cycloadducts in high yields along with excellent diastereo- and enantioselectivities [51]. Herein we wish to report the development of a multifunctional thiourea-phosphine catalyzed asymmetric [3 + 2] annulation of MBH carbonates with maleimides, which can efficiently construct functionalized cyclopentene derivatives bearing three contiguous stereocenters in moderate to excellent yields along with excellent diastereo- and enantioselectivities.

## Results and Discussion

In our previous work, it was shown that chiral multifunctional thiourea-phosphines were excellent catalysts for the asymmetric aza-MBH reaction, asymmetric allylic substitution of MBH adducts, and asymmetric [3 + 2] annulation of MBH carbonates with electron-deficient olefins [52–56]. Hence, we initially used multifunctional thiourea-phosphine (TP), which could be easily prepared from L-phenylalanine in four steps [57], as a catalyst to investigate the asymmetric [3 + 2] annulation of *N*-phenylmaleimide (**1a**) with MBH carbonate **2a** in toluene at room temperature. We were pleased to find that the corresponding highly functionalized cyclopentene **3a** was obtained in 54% yield with 96% ee after 24 h (Table 1, entry 1). The examination of solvent effects revealed that toluene is the best solvent (Table 1, entries 2–4). Increasing the ratio of **1a**/**2a** from 1:1.3 to 2:1 afforded **3a** in 74% yield and 96% ee, while continuously increasing the ratio of **1a**/**2a** to 4:1 gave **3a** in lower yield (Table 1, entries 5 and 6).

**Table 1:** Optimization of the reaction conditions for asymmetric [3 + 2] annulation of MBH carbonates and maleimides.

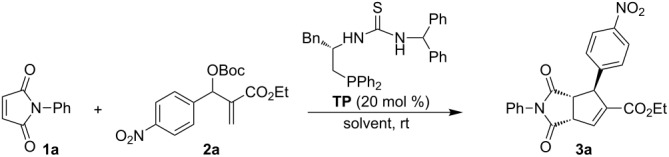				

entry^a^	**1a**/**2a**	solvent	yield (%)^b^	ee (%)^c^

1	1/1.3	toluene	54	96
2	1/1.3	CHCl_3_	54	96
3	1/1.3	THF	25	90
4	1/1.3	CH_3_CN	12	43
5^d^	2/1	toluene	74	96
6^e^	4/1	toluene	62	96

^a^Reactions were performed with **1a** (0.1 mmol) and **2a** (0.13 mmol) in toluene (1.0 mL) at room temperature for 24 h. ^b^Isolated yield of major isomer. ^c^Determined by chiral HPLC. ^d^Reaction was performed with **1a** (0.2 mmol) and **2a** (0.1 mmol) in toluene (1.0 mL) at room temperature for 24 h. ^e^Reaction was performed with **1a** (0.4 mmol) and **2a** (0.1 mmol) in toluene (1.0 mL) at room temperature for 24 h.

**Figure 1:**
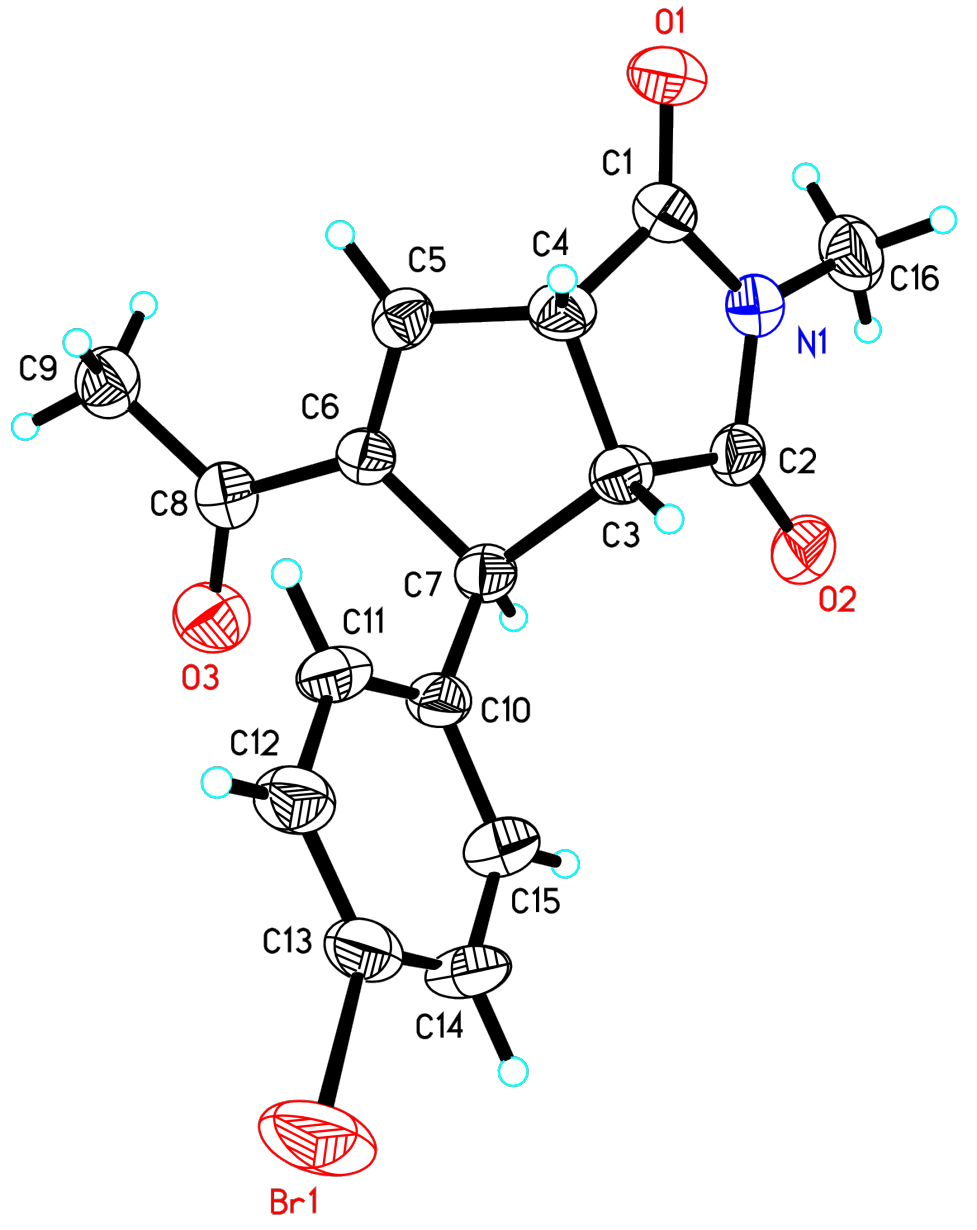
The ORTEP plot of compound **3r**.

Having identified the optimal reaction conditions, we next set out to examine the scope and limitations of this asymmetric [3 + 2] annulation of maleimides with MBH carbonates, and the results are summarized in Table 2. All of the reactions proceeded smoothly under the optimal conditions, providing annulation products with good to excellent diastereo- and enantioselectivities. Using MBH carbonate **2a** as a substrate, we examined its reaction with maleimides **1b**–**1e** and found that the reactions proceeded smoothly to give the corresponding products **3b**–**3e** in excellent yields along with excellent ee values (Table 2, entries 2–5). Taking *N*-benzhydrylmaleimide (**1d**) as a substrate, we found that substrates with an electron-withdrawing substituent on the aromatic ring of MBH carbonates **2** produced **3f** and **3g** in good yields and excellent ee values (Table 2, entries 6 and 7), and MBH carbonate **2d** gave the corresponding annulation product **3h** in 3:1 dr value with 74% yield of major isomer and 97% ee (Table 2, entry 8). A variety of MBH carbonates **2** having either electron-donating or -withdrawing groups as substituents at the *para*- and *meta*-position of the benzene ring and *N*-methylmaleimide (**1b**) underwent this asymmetric [3 + 2] annulation smoothly, affording the corresponding products **3** in moderate to excellent yields with excellent ee values upon lengthening of the reaction time (Table 2, entries 9–11, 13–15). Substrate **2k**, incorporating a heteroaromatic group, could also react with **1b** under the same reaction conditions, giving the corresponding cyclopentene **3p** in 69% yield with 96% ee, and substrates **2l** and **2m** derived from methyl vinyl ketone (MVK) afforded the corresponding annulation products **3q** and **3r** in excellent yields and ee values under the standard conditions (Table 2, entries 16–18). The absolute configuration of the major product **3r** was determined as (1*S*,2*R*,3*S*) by X-ray crystal structure. Its ORTEP plot is shown in Figure 1 and the corresponding CIF data are presented in the Supporting Information File 1 [58]. As for MBH carbonate **2g**, having a chlorine substituent at the *ortho*-position of the aromatic ring, this produced **3l** in lower yield and ee value, perhaps due to the steric effect; reaction of MBH carbonate **2n** derived from isobutyraldehyde did not occur (entries 12 and 19). When we utilized dimethyl maleate or dimethyl fumarate instead of maleimides, it was found that the reactions could not be performed either.

**Table 2:** Substrate scope of asymmetric [3 + 2] annulation.

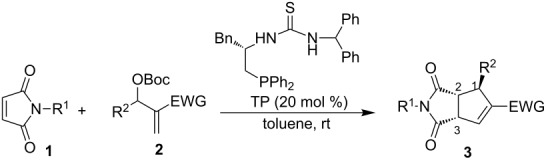					

entry^a^	R^1^	R^2^	EWG	yield (%)^b^	ee (%)^c^

1	**1a**, Ph	**2a**, 4-NO_2_C_6_H_4_	CO_2_Et	**3a**, 74	96
2	**1b**, Me	**2a**, 4-NO_2_C_6_H_4_	CO_2_Et	**3b**, 99	98
3	**1c**, Bn	**2a**, 4-NO_2_C_6_H_4_	CO_2_Et	**3c**, 86	94
4	**1d**, benzhydryl	**2a**, 4-NO_2_C_6_H_4_	CO_2_Et	**3d**, >99	98
5	**1e**, 1-methylnaphthyl	**2a**, 4-NO_2_C_6_H_4_	CO_2_Et	**3e**, 91	96
6	**1d**, benzhydryl	**2b**, 3-NO_2_C_6_H_4_	CO_2_Et	**3f**, 87	94
7	**1d**, benzhydryl	**2c**, 4-CNC_6_H_4_	CO_2_Et	**3g**, 84	96
8^d,e^	**1d**, benzhydryl	**2d**, 4-BrC_6_H_4_	CO_2_Et	**3h**, 74	97
9^e^	**1b**, Me	**2d**, 4-BrC_6_H_4_	CO_2_Et	**3i**, 79	95
10^e^	**1b**, Me	**2e**, 4-ClC_6_H_4_	CO_2_Et	**3j**, 90	96
11^e^	**1b**, Me	**2f**, 3-ClC_6_H_4_	CO_2_Et	**3k**, 81	97
12^f^	**1b**, Me	**2g**, 2-ClC_6_H_4_	CO_2_Et	**3l**, 39	73
13^f^	**1b**, Me	**2h**, C_6_H_5_	CO_2_Et	**3m**, 74	94
14^f^	**1b**, Me	**2i**, 4-MeC_6_H_4_	CO_2_Et	**3n**, 64	96
15^f^	**1b**, Me	**2j**, 4-MeOC_6_H_4_	CO_2_Et	**3o**, 55	98
16^f^	**1b**, Me	**2k**, 2-furyl	CO_2_Et	**3p**, 69	96
17	**1b**, Me	**2l**, 4-NO_2_C_6_H_4_	COMe	**3q**, >99	97
18	**1b**, Me	**2m**, 4-NO_2_C_6_H_4_	COMe	**3r**, 92	98
19^f^	**1b**, Me	**2n**, (CH_3_)_2_CH	CO_2_Et	–	–

^a^Reactions were performed with **1** (0.2 mmol), **2** (0.1 mmol) in toluene (1.0 mL) at room temperature for 24 h. ^b^Isolated yield of major isomer. ^c^Determined by chiral HPLC. ^d^dr = 3:1. ^e^Reaction was performed for 36 h. ^f^Reaction was performed for 48 h.

**Figure 2:**
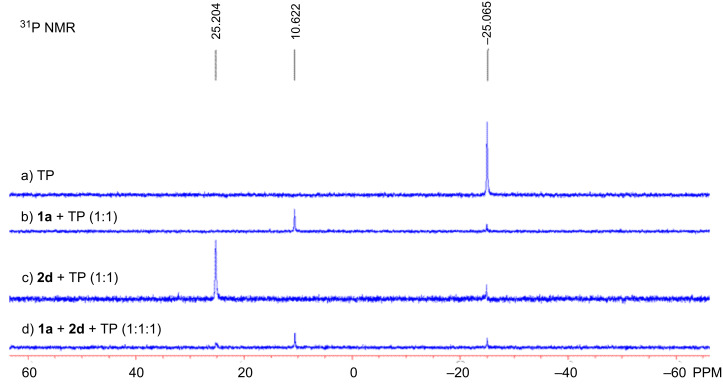
^31^P NMR spectra (161.9 MHz, CDCl_3_) of control experiments.

As aforementioned, increasing the ratio of **1a**/**2a** from 1:1.3 to 2:1 obtained the highest yield. In order to explain this finding, three control experiments were performed in CDCl_3_ and the results were studied by using ^31^P NMR spectroscopy (Figure 2, also see Figure SI-1 in the Supporting Information File 2). The NMR studies revealed that catalyst TP seems to react with *N*-phenylmaleimide (**1a**) to form a new species (Figure 2, spectra b), which costs some maleimide **1a**. Thus, the excess **1a** has to be added in order to make the reaction complete. This is why increasing the ratio of **1a**/**2a** raised the yield of product **3a** (Table 1, entry 5). On the other hand, if too much maleimide **1a** is used in this reaction, it will waste some of the catalyst TP, leading to a decrease in the yield of product **3a** (Table 1, entry 6). The allylic phosphorus ylide species can be recognized in spectra c from the combination of **2d** and TP (1:1) (Figure 2, also see Figure SI-1 in the Supporting Information File 2) [59].

**Scheme 2:**
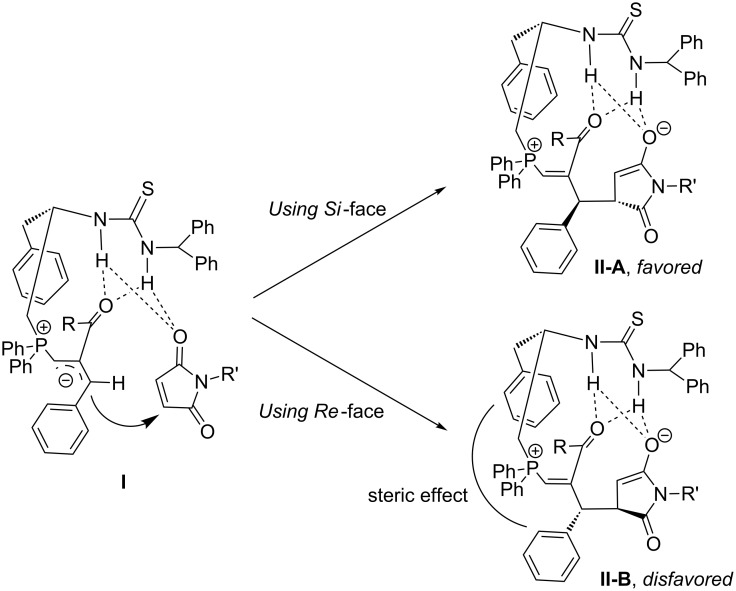
Proposed transition models.

As reported before [15,51], TP attacks MBH carbonate to afford allylic phosphorus ylide **I**, which attacked the maleimide to produce intermediate **II** (Scheme 2). Since there is a steric effect between the phenyl group and the benzyl group in intermediate **II-B**, allylic phosphorus ylide **I** using its *Si*-face to attack maleimide is favored (intermediate **II-A**). Undergoing Michael addition and elimination of catalyst along with the double-bond formation, intermediate **I** affords the corresponding highly functionalized cyclopentene product and completes the catalytic cycle. The control experiments demonstrated that catalyst TP can react with maleimide, affording an unidentified complex and partially wasting maleimide and catalyst (spectra d in Figure 2). Hypothetically, the activity of the in situ generated allylic phosphorous ylide **I** is crucial for the yield of product. If the activity of phosphorous ylide is not high enough, it may deprotonate the NH proton in TP, which will cause the catalyst to lose activity (See Table 2, entries 12–16). In addition, if the phosphorous ylide is not active enough to react with maleimide, maleimide can directly react with the catalyst to form the other unidentified species as indicated in the ^31^P NMR spectroscopy (spectra b in Figure 2), which may retard the desired catalytic cycle.

**Scheme 3:**
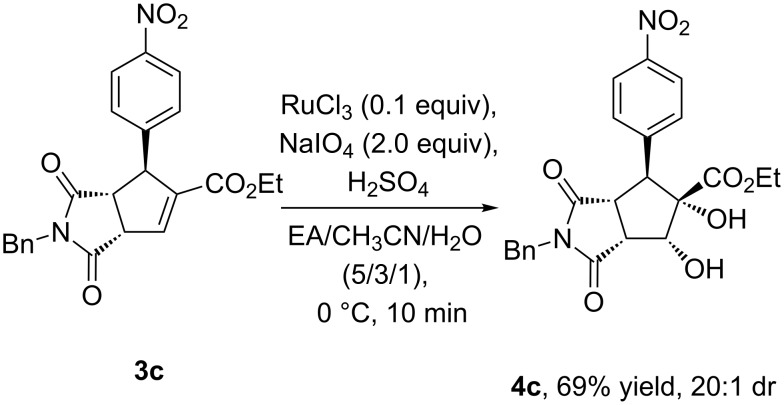
Dihydroxylation of **3c**.

To illustrate the synthetic utility of these products **3** obtained from the above asymmetric [3 + 2] annulation, the further transformation of **3c** was performed in the presence of RuCl_3_ and NaIO_4_ under mild conditions (Scheme 3) [22,60]. Upon dihydroxylation of **3c**, the corresponding product **4c** containing five stereocenters was produced in 69% yield and good diastereoselectivity on the basis of NMR spectroscopic data (dr = 20:1, see Supporting Information File 2).

## Conclusion

In conclusion, we have developed a novel multifunctional thiourea-phosphine catalyzed asymmetric [3 + 2] annulation of MBH carbonates with maleimides, which can efficiently construct functionalized cyclopentenes bearing three contiguous stereocenters in moderate to excellent yields and excellent diastereo- and enantioselectivities, and the product can be efficiently transformed into a cyclopentane containing five stereocenters under mild conditions, which was difficult to construct by other synthetic methodologies. Current efforts are in progress to apply this new methodology to synthesize biologically active products.

## Experimental

### General procedure for asymmetric [3 + 2] annulation

Under an argon atmosphere, a mixture of maleimide **1** (0.2 mmol), MBH carbonate **2** (0.2 mmol) and catalyst TP (0.02 mmol, 11 mg) in toluene (1.0 mL) was stirred at room temperature for 24–48 h. Then the solvent was removed under reduced pressure, and the residue was chromatographed on silica gel (elution with petroleum ether/EtOAc 10:1–4:1) to provide compound **3**.

## Supporting Information

File 1 Experimental procedures and characterization data of compounds given in this article.

File 2 Crystal structure data of compound **3r**.

File 3 cif data of **3r**.
