# Supplementary material for: Chiral multifunctional thiourea-phosphine catalyzed asymmetric [3 + 2] annulation of Morita–Baylis–Hillman carbonates with maleimides
Source: Beilstein J Org Chem. 2012 Jul 16;8:1098–104. doi: 10.3762/bjoc.8.121 (PMC3458726; doi:10.3762/bjoc.8.121)
Supplement: File 1 — Experimental procedures and characterization data of compounds given in this article. [file Beilstein_J_Org_Chem-08-1098-s001.pdf]

Supporting Information

for

Chiral multifunctional thiourea-phosphine catalyzed asymmetric [3 + 2] annulation of Morita–Baylis–Hillman carbonates with maleimides

Hong-Ping Deng[1], De Wang[2], Yin Wei[1] and Min Shi*[1,2,§]

Address: [1]State Key Laboratory of Organometallic Chemistry, Shanghai Institute of Organic Chemistry, Chinese Academy of Sciences, 354 Fenglin Road, Shanghai 200032, People's Republic of China and [2]Key Laboratory for Advanced Materials and Institute of Fine Chemicals, School of Chemistry & Molecular Engineering, East China University of Science and Technology, and 130 MeiLong Road, Shanghai 200237, People's Republic of China

Email: Min Shi - Mshi@mail.sioc.ac.cn

[§]Fax: 86-21-64166128

*Corresponding author

Experimental procedures and characterization data of compounds given in this article

CONTENTS

1. General procedure for asymmetric [3 + 2] annulation…......……………………….……........S2

2. General procedure and spectroscopic data of the product **4c**…..…………………………..…..S56

3. ^{31}P NMR Spectroscopy of the control experiments..……………………………………..S60

4. References…………………………………………………………...…………………..…...S62

General remarks:

Melting points were determined on a digital melting point apparatus and temperatures were uncorrected. Optical rotations were determined at 589 nm (sodium D line) by using a Perkin-Elmer-341 MC digital polarimeter; $[\alpha]_D$-values are given in units of 10 deg^{-1} cm^2 g^{-1}. ^{1}H NMR spectra were recorded on a Bruker AM-300 and AM-400 spectrometer for solution in $CDCl_3$ with tetramethylsilane (TMS) as an internal standard; coupling constants *J* are given in Hertz. ^{13}C NMR spectra were recorded on a Bruker AM-300 and AM-400 spectrophotometers (75 or 100 MHz) with complete proton decoupling spectrophotometers ($CDCl_3$: 77.0 ppm). ^{31}P NMR spectra were recorded on a Bruker AM-400 spectrophotometers (161.9 MHz) for solution in $CDCl_3$ with 85% H_3PO_4 as an internal standard; infrared spectra were recorded on a Perkin-Elmer PE-983 spectrometer with absorption in cm^{-1}. Flash column chromatography was performed with 300–400 mesh silica gel. For thin-layer chromatography (TLC), silica gel plates (Huanghai GF254) were used. Chiral HPLC was performed on a SHIMADZU SPD-10A *vp* series with chiral columns (Chiralpak AD-H, OD-H, and IC-H columns 4.6 × 250 mm, (Daicel Chemical Ind., Ltd.)) and chiral column (Phenomenex Lux 5µ Amylose-2 column 4.6 × 250 mm (PA-2, (Phenomenex Ind., Ltd.)). Mass spectra were recorded by EI, ESI, MALDI and HRMS, measured on a HP-5989 instrument.

1. General procedure for asymmetric [3 + 2] annulation.

Catalyst **TP** was synthesized according to the literature [1], and its yield, characterization and spectra are provided in [1].

General Procedure for Asymmetric [3 + 2] Annulation:

Under an argon atmosphere, a mixture of maleimide **1** (0.2 mmol), MBH carbonate **2** (0.2 mmol) and catalyst **TP** (0.02 mmol, 11 mg) in toluene (1.0 mL) was stirred at room temperature for 24–48 h. Then the solvent was removed under reduced pressure, and the residue was chromatographed on silica gel (elution with petroleum ether/EtOAc 10:1–4:1) to provide compound **3**.

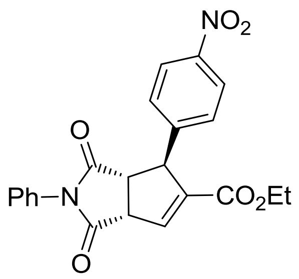

(3a*R*,4*S*,6a*S*)-ethyl 4-(4-nitrophenyl)-1,3-dioxo-2-phenyl-1,2,3,3a,4,6a-hexahydro-cyclopenta[*c*]pyrrole-5-carboxylate (3a). Yield: 30 mg, 74%; yellow oil; IR (CH_2Cl_2): ν 3076, 2981, 1779, 1716, 1635, 1597, 1519, 1496, 1456, 1348, 1273, 1181, 1089, 1053, 1014, 988, 889, 852, 814, 769, 752, 734, 692, 661 cm^{-1}; ^{1}H NMR (400 MHz, $CDCl_3$, TMS): δ 1.19 (3H, t, J = 7.2 Hz, CH_3), 3.52 (1H, dd, J = 2.4, 7.2 Hz, CH), 4.06–4.18 (2H, m, CH_2), 4.34–4.38 (1H, m, CH), 4.77 (1H, d, J = 1.2 Hz, CH), 7.06 (1H, dd, J = 1.6, 2.4 Hz, CH), 7.28–7.30 (2H, m, ArH), 7.40–7.44 (3H, m, ArH), 7.47–7.51 (2H, m, ArH), 8.22 (2H, d, J = 8.8 Hz, ArH); ^{13}C NMR (100 MHz, $CDCl_3$, TMS): δ 13.9, 52.2, 52.9, 53.2, 61.3, 124.3, 126.3, 128.0, 129.0, 131.2, 138.6, 140.8, 147.3, 148.9, 162.4, 173.7, 176.3; MS (ESI) *m/z* (%): 407.0 (100) [M^+ + 1]; HRMS (ESI) Calcd. for $C_{22}H_{18}N_2NaO_6$ (M^+ + Na) requires 429.1057, found: 429.1062; Enantiomeric excess was determined by HPLC with a Chiralcel IC-H column [λ = 214 nm; eluent: hexane/isopropanol 50/50; flow rate: 0.7 mL/min; t_{major} = 28.13 min, t_{minor} = 38.19 min; ee = 96%; $[\alpha]^{20}_D$ = +383.9 (*c* 1.0, $CHCl_3$)].

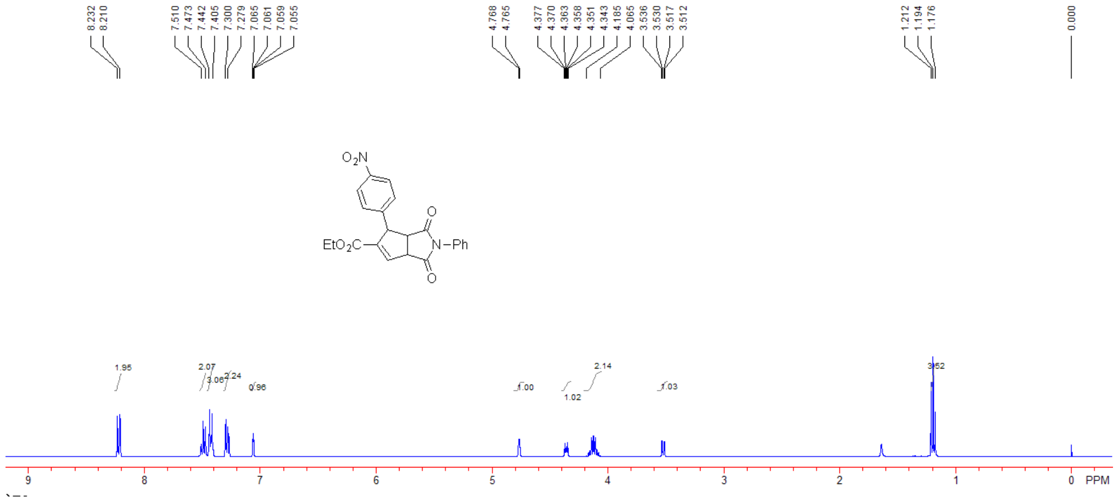

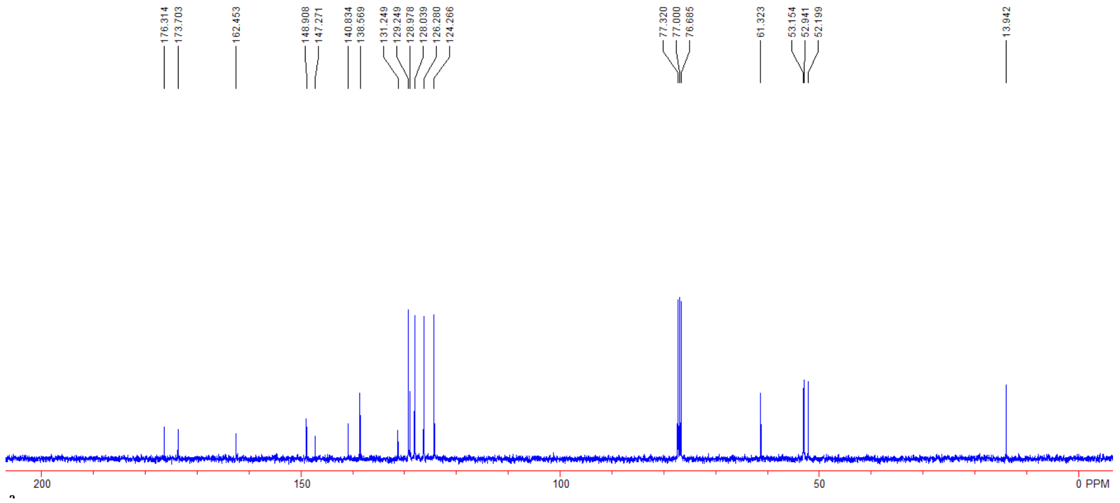

N2000 数据工作站 1

实验时间：2011-07-11, 16:06:09
谱图文件:E:\实验数据\HPLC\dhp\dhp-17-24-IC-50-50-0.7-214-rac.org

实验者：
报告时间：2011-11-18, 21:53:10
积分方法:面积归一法

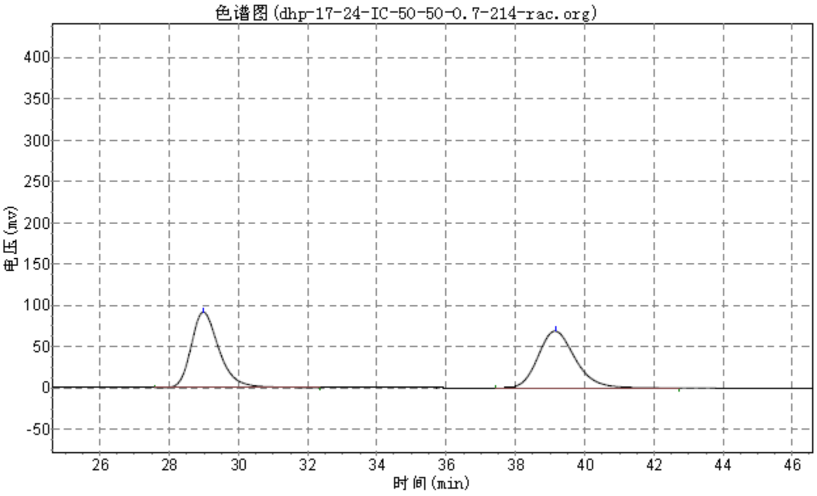

分析结果表

峰号	峰名	保留时间	峰高	峰面积	含量
1		28.998	91347.523	5141725.500	49.9864
2		39.165	68916.156	5144520.500	50.0136
**总计**			160263.680	10286246.000	100.0000

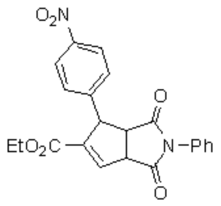

rac

IC-H, 214 nm
Hexane:^{i}PrOH = 50:50
0.7 mL/min

N2000 数据工作站 1

实验时间：2011-07-14,17:13:46
谱图文件:E:\实验数据\HPLC\dhp\dhp-17-45-IC-50-50-0.7-214-asy.org

实验者：
报告时间：2011-11-18,21:54:09
积分方法:面积归一法

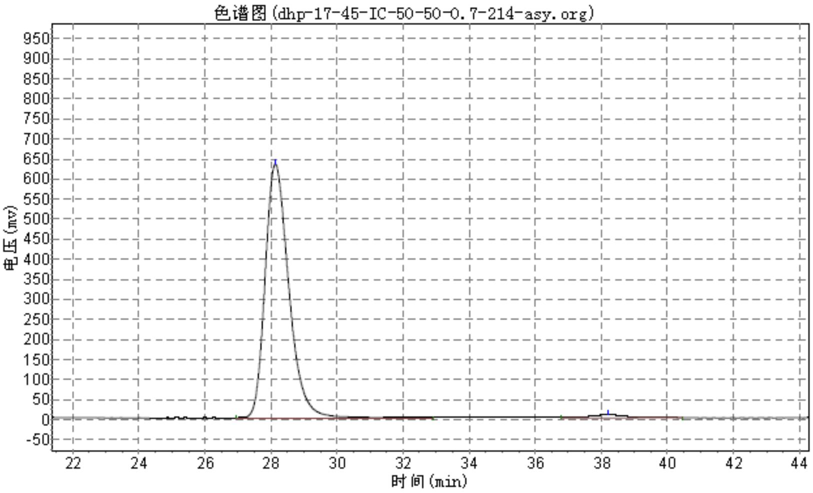

分析结果表

峰号	峰名	保留时间	峰高	峰面积	含量
1		28.130	633080.813	31597968.000	98.1329
2		38.193	8477.035	601178.625	1.8671
总计			641557.848	32199146.625	100.0000

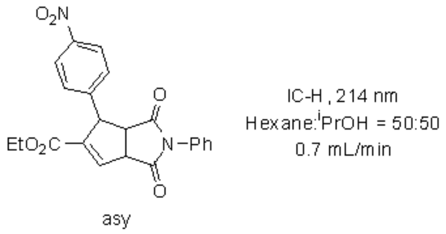

Chiral HPLC report: Enantiomeric excess was determined by HPLC with a Chiralcel IC-H column [λ = 214 nm; eluent: hexane/isopropanol 50/50; flow rate: 0.7 mL/min; t_{major} = 28.13 min, t_{minor} = 38.19 min; ee = 96%; $[\alpha]^{20}_{D}$ = +338.9 (*c* 1.0, $CHCl_3$)].

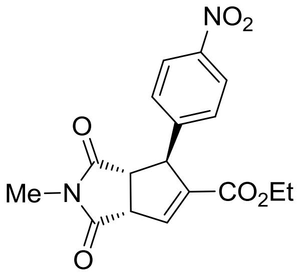

(3a*R*,4*S*,6a*S*)-ethyl 2-methyl-4-(4-nitrophenyl)-1,3-dioxo-1,2,3,3a,4,6a-hexahydrocyclo-penta[*c*]pyrrole-5-carboxylate (3b). Yield: 34 mg, 99%; yellow oil; IR (CH_2Cl_2): ν 2983, 1782,

1704, 1635, 1597, 1519, 1494, 1434, 134, 1273, 1203, 1096, 1045, 1014, 1000, 959, 894, 852, 814, 792, 755, 736, 700, 659 cm^{-1}; ^{1}H NMR (300 MHz, $CDCl_3$, TMS): δ 1.18 (3H, t, J = 7.2 Hz, CH_3), 3.03 (3H, s, CH_3), 3.36 (1H, dd, J = 2.1, 7.2 Hz, CH), 4.02–4.17 (2H, m, CH_2), 4.20–4.25 (1H, m, CH), 4.64 (1H, d, J = 1.2 Hz, CH), 6.98–6.99 (1H, m, CH), 7.40 (2H, d, J = 9.0 Hz, ArH), 8.21 (2H, d, J = 9.0 Hz, ArH); ^{13}C NMR (75 MHz, $CDCl_3$, TMS): δ 13.9, 25.2, 52.1, 52.7, 52.9, 61.2, 124.0, 128.0, 138.6, 140.4, 147.2, 149.1, 162.4, 174.8, 177.3; MS (ESI) m/z (%): 345.0 (100) [M^+ + 1]; HRMS (ESI) Calcd. for $C_{17}H_{16}N_2NaO_6$ (M^+ + Na) requires 367.0901, found: 367.0893; Enantiomeric excess was determined by HPLC with a Chiralcel IC-H column [λ = 214 nm; eluent: hexane/isopropanol 50/50; Flow rate: 0.7 mL/min; t_{major} = 39.74 min, t_{minor} = 46.57 min; ee = 98%; $[\alpha]^{20}_D$ = +282.8 (c 1.0, $CHCl_3$)].

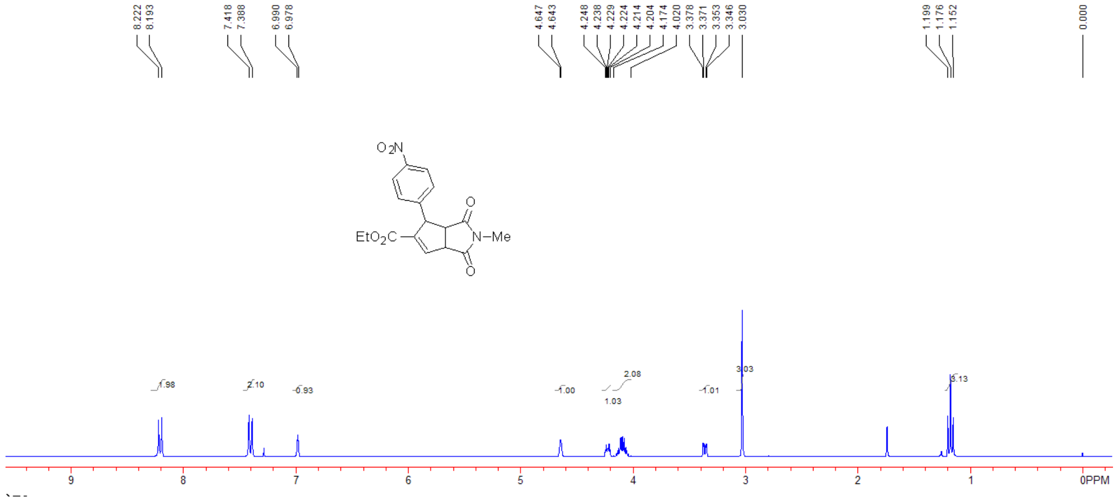

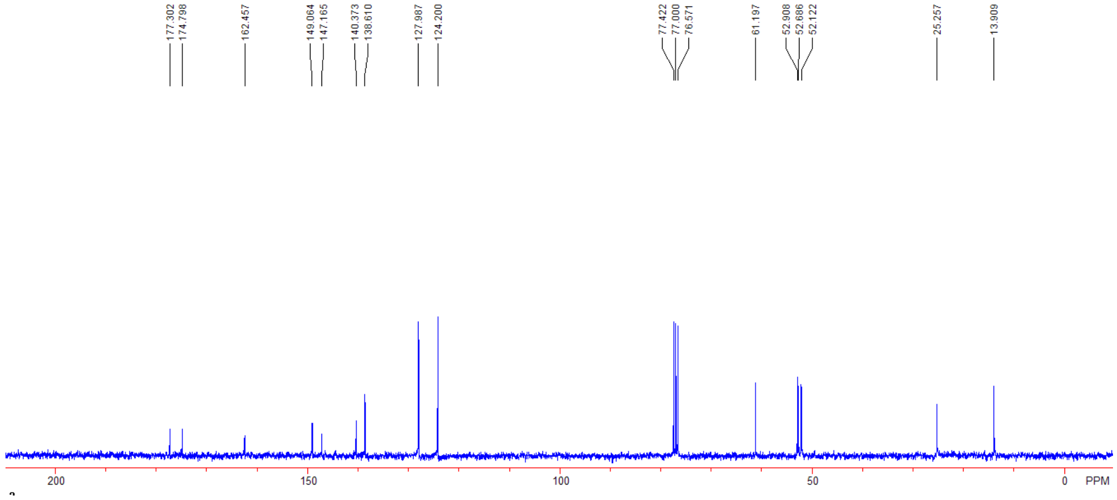

实验时间：2011-08-17,16:23:31
谱图文件:E:\实验数据\HPLC\dhp\dhp-17-73-1-N-Me-IC-50-50-0.7-214-rac.org

实验者：
报告时间：2011-11-18,21:46:17
积分方法:面积归一法

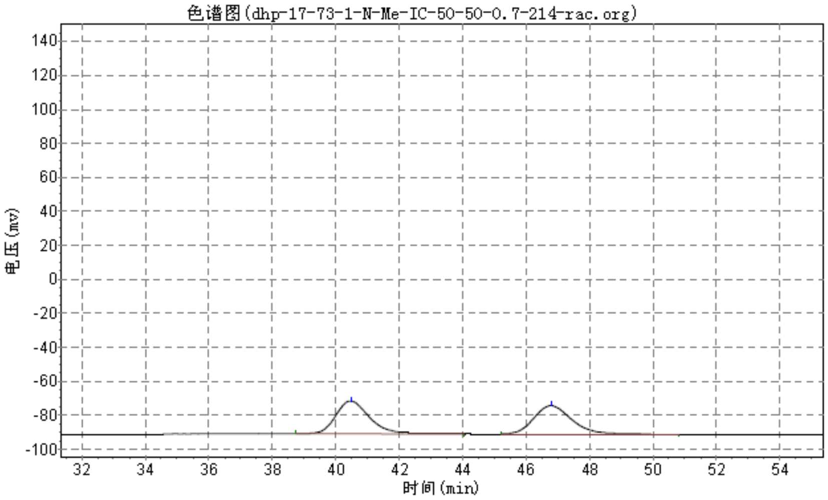

分析结果表

峰号	峰名	保留时间	峰高	峰面积	含量
1		40.498	19236.787	1412594.375	50.7159
2		46.798	16573.801	1372712.250	49.2841
**总计**			35810.588	2785306.625	100.0000

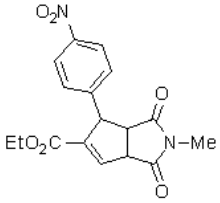

rac

IC-H , 214 nm
Hexane:^{i}PrOH = 50:50
0.7 mL/min

N2000 数据工作站 1

实验时间：2011-08-17, 17:26:10
谱图文件:E:\实验数据\HPLC\dhp\dhp-17-73-1-N-Me-IC-50-50-0.7-214-asy.org

实验者：
报告时间：2011-11-18, 21:48:57
积分方法:面积归一法

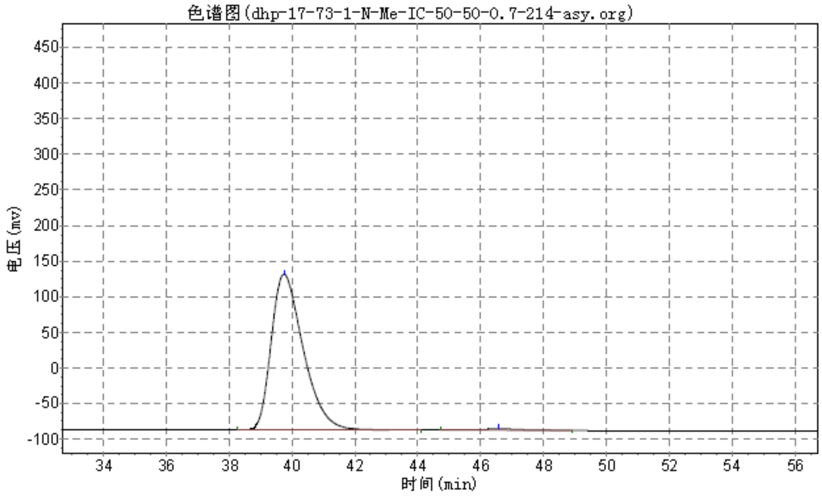

分析结果表

峰号	峰名	保留时间	峰高	峰面积	含量
1		39.743	218998.922	15517876.000	99.0861
2		46.575	1748.814	143119.609	0.9139
**总计**			220747.736	15660995.609	100.0000

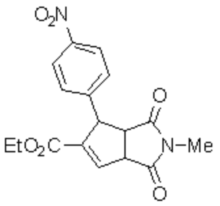

IC-H, 214 nm
Hexane:^{i}PrOH = 50:50
0.7 mL/min

asy

Chiral HPLC Report: Enantiomeric excess was determined by HPLC with a Chiralcel IC-H column [λ = 214 nm; eluent: hexane/isopropanol 50/50; flow rate: 0.7 mL/min; t_{major} = 39.74 min, t_{minor} = 46.57 min; ee = 98%; $[\alpha]^{20}_{D}$ = +282.8 (*c* 1.0, $CHCl_3$)].

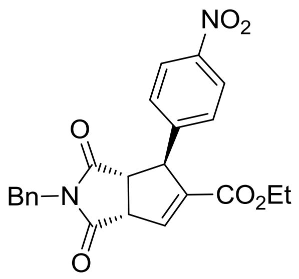

(3a*R*,4*S*,6a*S*)-ethyl 2-benzyl-4-(4-nitrophenyl)-1,3-dioxo-1,2,3,3a,4,6a-hexahydrocyclo-penta[*c*]pyrrole-5-carboxylate (3c). Yield: 36 mg, 86%; yellow oil; IR (CH_2Cl_2): ν 3077, 3033,

2981, 2936, 1776, 1708, 1635, 1604, 1558, 1455, 1431, 1393, 1348, 1271, 1202, 1169, 1095, 1054, 1014, 991, 931, 905, 851, 814, 753, 714, 699, 660 cm^{-1}; ^{1}H NMR (400 MHz, $CDCl_3$, TMS): δ 1.16 (3H, t, J = 7.2 Hz, CH_3), 3.34 (1H, dd, J = 2.4, 7.2 Hz, CH), 4.04–4.13 (2H, m, CH_2), 4.18–4.21 (1H, m, CH), 4.62–4.70 (3H, m, CH_2+CH), 6.97 (1H, s, CH), 7.26–7.38 (7H, m, ArH), 8.18–8.20 (2H, m, ArH); ^{13}C NMR (100 MHz, $CDCl_3$, TMS): δ 13.9, 42.8, 52.2, 52.7, 52.9, 61.2, 124.2, 127.9, 128.2, 128.72, 128.75, 135.1, 138.6, 140.4, 147.2, 149.1, 162.4, 174.4, 176.9; MS (ESI) m/z (%): 421.1 (100) [M^{+}+1]; HRMS (ESI) Calcd. for $C_{23}H_{20}N_2NaO_6$ (M^{+} + Na) requires 443.1214, found: 443.1198; Enantiomeric excess was determined by HPLC with a Chiralcel IC-H column [λ = 214 nm; eluent: hexane/isopropanol 50/50; flow rate: 0.7 mL/min; t_{major} = 29.77 min, t_{minor} = 33.60 min; ee = 94%; $[\alpha]^{20}_{D}$ = +243.3 (c 1.0, $CHCl_3$)].

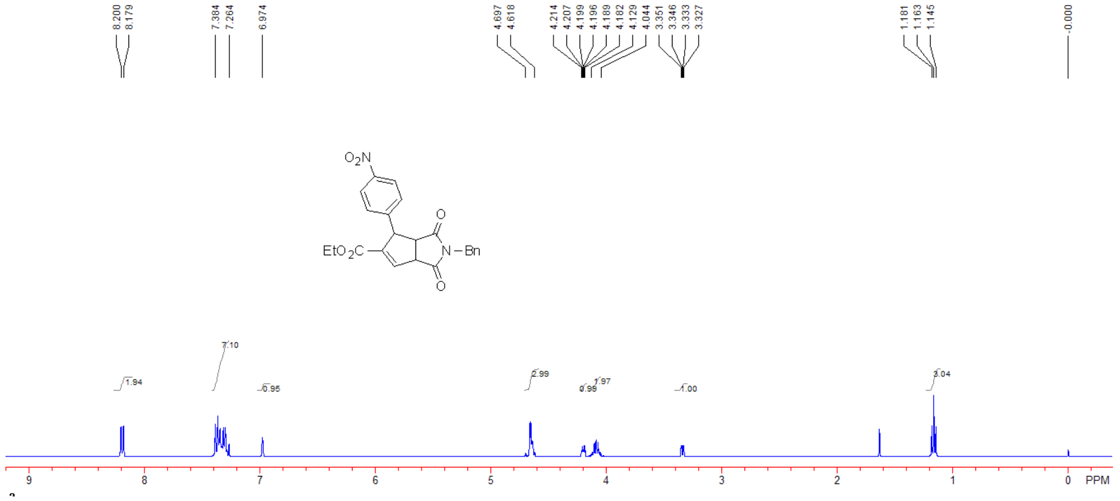

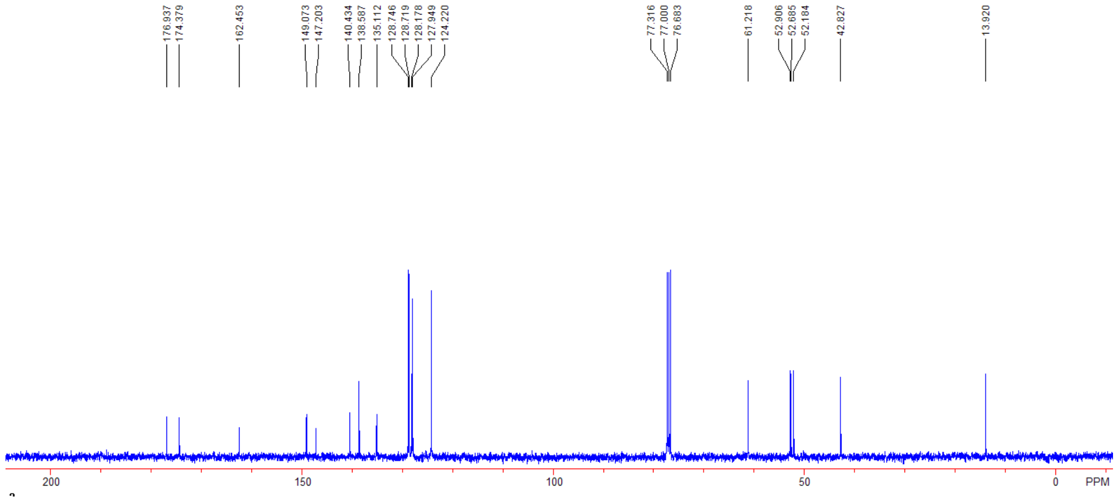

实验时间：2011-07-21,23:15:38
谱图文件:E:\实验数据\HPLC\dhp\dhp-17-59-IC-50-50-0.7-214-rac.org

实验者：
报告时间：2011-11-18,21:54:45
积分方法:面积归一法

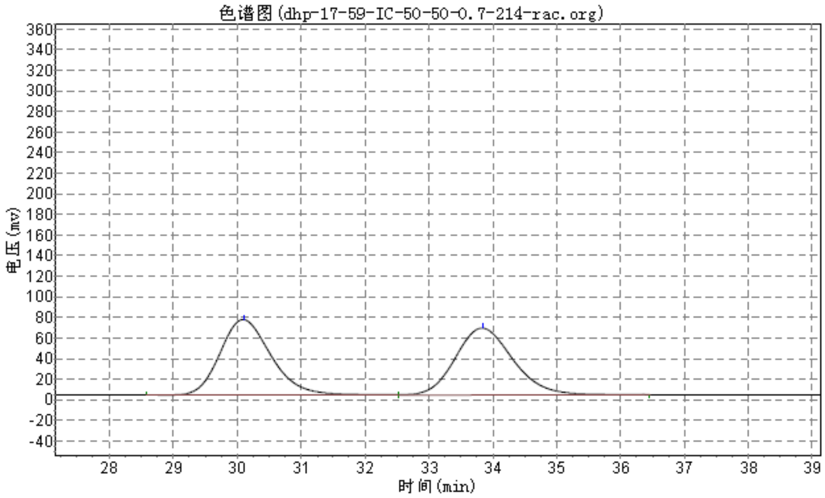

分析结果表

峰号	峰名	保留时间	峰高	峰面积	含量
1		30.123	73285.547	4081439.000	50.1095
2		33.857	64830.836	4063593.750	49.8905
**总计**			138116.383	8145032.750	100.0000

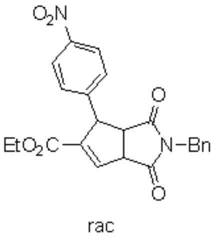

IC-H, 214 nm
Hexane:^{i}PrOH = 50:50
0.7 mL/min

实验时间：2011-07-21,22:28:06
谱图文件:E:\实验数据\HPLC\dhp\dhp-17-59-IC-50-50-0.7-214-asy.org

实验者：
报告时间：2011-11-18,21:55:47
积分方法:面积归一法

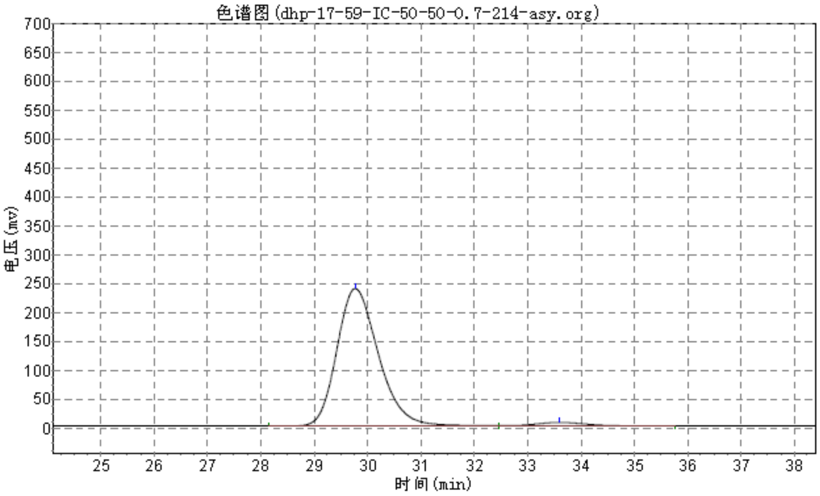

分析结果表

峰号	峰名	保留时间	峰高	峰面积	含量
1		29.773	237640.906	12853007.000	96.9194
2		33.605	6289.451	408535.344	3.0806
**总计**			243930.357	13261542.344	100.0000

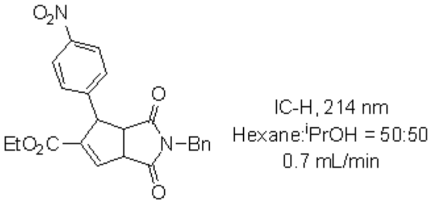

asy

Chiral HPLC report: Enantiomeric excess was determined by HPLC with a Chiralcel IC-H column [λ = 214 nm; eluent: hexane/isopropanol 50/50; flow rate: 0.7 mL/min; t_{major} = 29.77 min, t_{minor} = 33.60 min; ee = 94%; $[\alpha]^{20}_{D}$ = +243.3 (*c* 1.0, $CHCl_3$)].

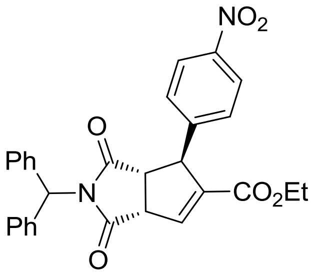

(3a*R*,4*S*,6a*S*)-ethyl 2-benzhydryl-4-(4-nitrophenyl)-1,3-dioxo-1,2,3,3a,4,6a-hexahydrocyclo-penta[*c*]pyrrole-5-carboxylate (3d). Yield: 50 mg, >99%; yellow oil; IR (CH_2Cl_2): ν 3064, 3030,

2924, 2853, 1776, 1712, 1634, 1598, 1520, 1495, 1449, 1348, 1272, 1190, 1170, 1094, 1031, 1014, 988, 920, 895, 853, 810, 738, 699, 663 cm^{-1}; ^{1}H NMR (300 MHz, $CDCl_3$, TMS): δ 1.18 (3H, t, J = 7.2 Hz, CH_3), 3.36 (1H, dd, J = 2.1, 7.8 Hz, CH), 4.06–4.16 (2H, m, CH_2), 4.19–4.23 (1H, m, CH), 4.68 (1H, d, J = 1.2 Hz, CH), 6.52 (1H, s, CH), 7.00 (1H, d, J = 1.2 Hz, CH), 7.23–7.28 (4H, m, ArH), 7.31–7.38 (8H, m, ArH), 8.17–8.20 (2H, m, ArH); ^{13}C NMR (75 MHz, $CDCl_3$, TMS): δ 13.9, 52.0, 52.8, 52.9, 58.7, 61.2, 124.2, 127.9, 128.0, 128.2, 128.42, 128.46, 128.5, 136.8, 136.9, 138.8, 140.5, 147.2, 149.0, 162.5, 174.2, 176.9; MS (ESI) m/z (%): 497.0 (100) [M^+ + 1]; HRMS (ESI) Calcd. for $C_{29}H_{24}N_2NaO_6$ (M^+ + Na) requires 519.1527, found: 519.1507; Enantiomeric excess was determined by HPLC with a Chiralcel IC-H column [λ = 214 nm; eluent: hexane/isopropanol 50/50; flow rate: 0.7 mL/min; t_{major} = 16.59 min, t_{minor} = 21.27 min; ee = 98%; $[\alpha]^{20}_{D}$ = +271.7 (c 1.0, $CHCl_3$)].

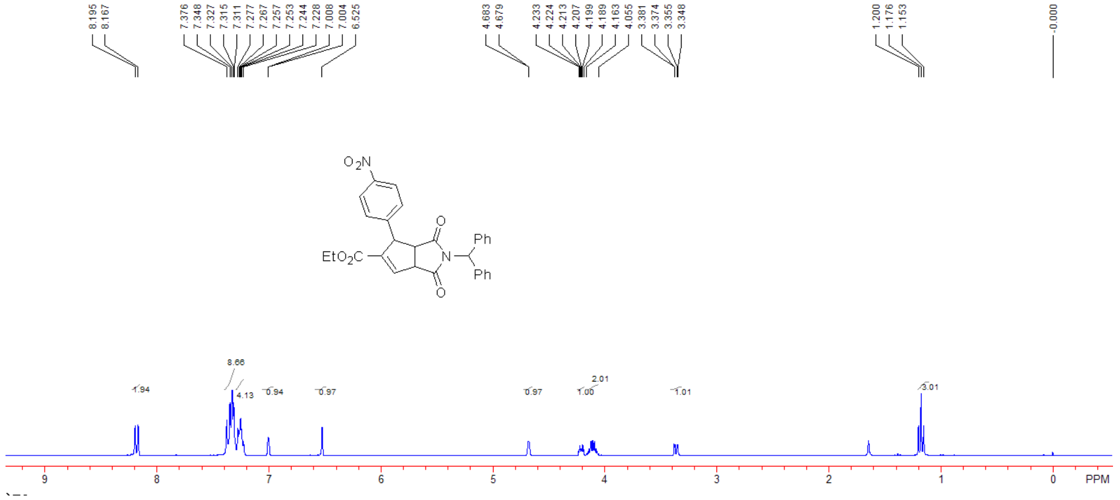

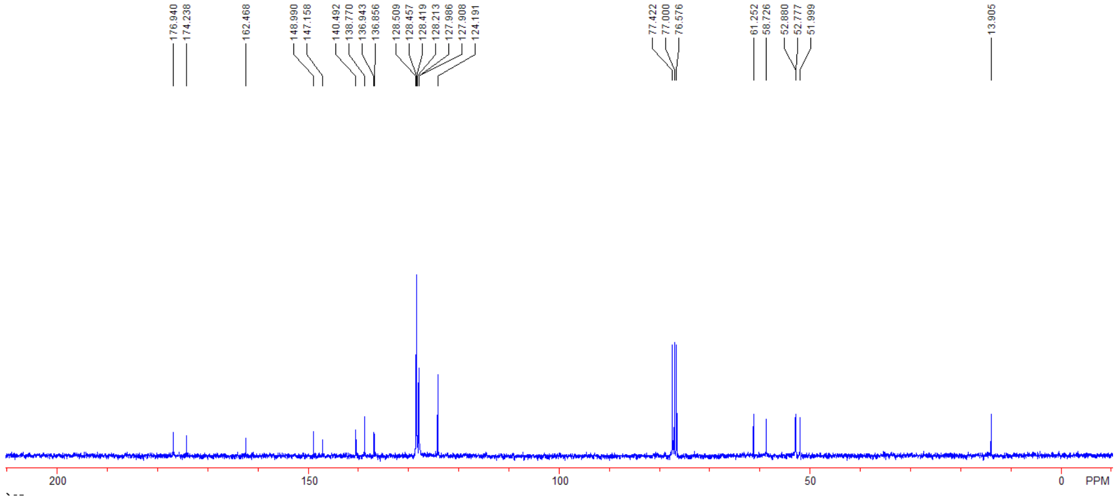

实验时间：2011-08-17,21:03:56
谱图文件:E:\实验数据\HPLC\dhp\dhp-17-73-2-N-CHPh2-IC-50-50-0.7-214-rac.org

实验者：
报告时间：2011-11-18,21:50:25
积分方法:面积归一法

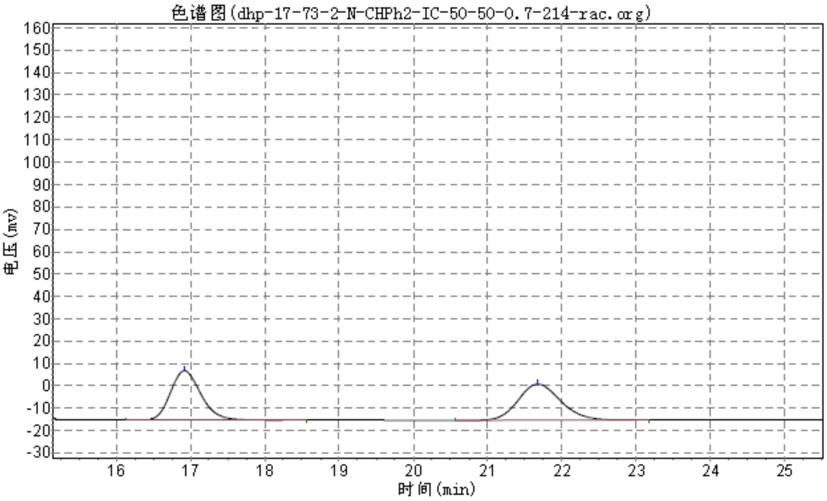

分析结果表

峰号	峰名	保留时间	峰高	峰面积	含量
1		16.918	22167.262	637809.063	49.5466
2		21.677	16221.405	649481.438	50.4534
**总计**			38388.667	1287290.500	100.0000

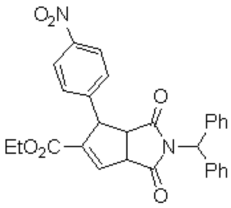

rac

IC-H, 214 nm
Hexane:^{i}PrOH = 50:50
0.7 mL/min

实验时间：2011-08-17, 21:54:52
谱图文件:E:\实验数据\HPLC\dhp\dhp-17-73-2-N-CHPh2-IC-50-50-0.7-214-asy.org

实验者：
报告时间：2011-11-18, 21:52:10
积分方法:面积归一法

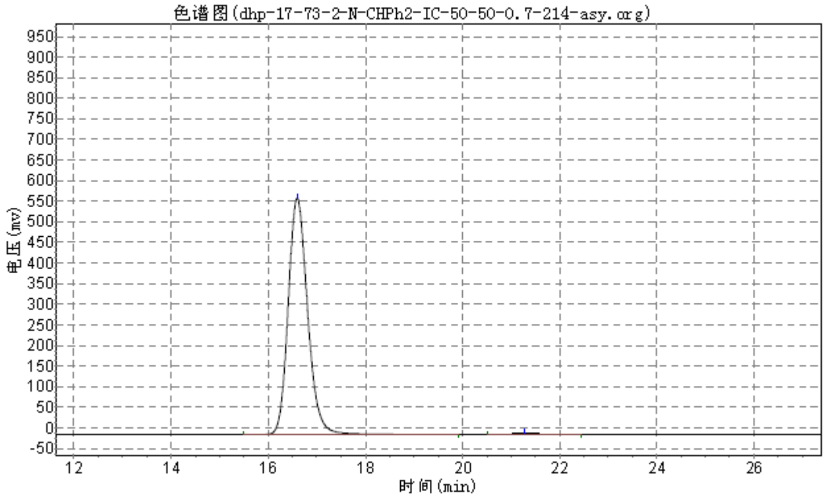

分析结果表

峰号	峰名	保留时间	峰高	峰面积	含量
1		16.593	573381.438	15920479.000	99.1817
2		21.275	3427.888	131353.188	0.8183
**总计**			576809.325	16051832.188	100.0000

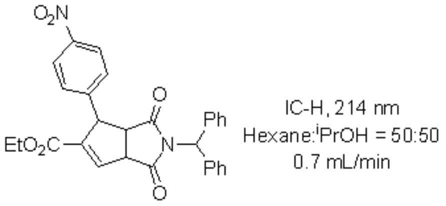

asy

Chiral HPLC report: Enantiomeric excess was determined by HPLC with a Chiralcel IC-H column [λ = 214 nm; eluent: hexane/isopropanol 50/50; flow rate: 0.7 mL/min; t_{major} = 16.59 min, t_{minor} = 21.27 min; ee = 98%; $[\alpha]^{20}_{D}$ = +271.7 (*c* 1.0, $CHCl_3$)].

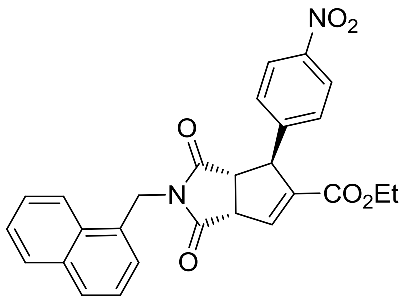

(3a*R*,4*S*,6a*S*)-ethyl 2-(naphthalen-1-ylmethyl)-4-(4-nitrophenyl)-1,3-dioxo-1,2,3,3a,4,6a-hexahydrocyclopenta[*c*]pyrrole-5-carboxylate (3e). Yield: 43 mg, 91%; yellow oil; IR (CH_2Cl_2): ν 2981, 2920, 1777, 1708, 1635, 1598, 1519, 1428, 1394, 1347, 1271, 1174, 1096, 1014, 990, 852, 792, 778, 736, 699 cm^{-1}; ^{1}H NMR (300 MHz, $CDCl_3$, TMS): δ 1.16 (3H, t, *J* = 6.9 Hz, CH_3), 3.30 (1H, dd, *J* = 1.8, 7.2 Hz, CH), 4.03–4.19 (3H, m, CH+CH_2), 4.65 (1H, d, *J* = 1.8 Hz, CH), 5.12 (2H, s, CH_2), 6.98 (1H, dd, *J* = 1.8, 2.7 Hz, CH), 7.33–7.36 (2H, m, ArH), 7.42 (1H, d, *J* = 7.5 Hz, ArH), 7.46–7.58 (3H, m, ArH), 7.78–7.86 (2H, m, ArH), 8.16 (2H, d, *J* = 8.7 Hz, ArH), 8.23 (1H, d, *J* = 8.1 Hz, ArH); ^{13}C NMR (75 MHz, $CDCl_3$, TMS): δ 13.9, 40.6, 52.0, 52.6, 52.8, 61.2, 123.2, 124.2, 125.1, 125.9, 126.6, 127.9, 128.7, 129.0, 130.1, 131.1, 133.7, 138.6, 140.4, 147.1, 149.0, 162.4, 174.5, 177.2; MS (ESI) *m/z* (%): 471.0 (100) [M^+ + 1]; HRMS (ESI) Calcd. for $C_{27}H_{22}N_2NaO_6$ (M^+ + Na) requires 493.1370, found: 493.1363; Enantiomeric excess was determined by HPLC with a Chiralcel PC-2 column [λ = 214 nm; eluent: hexane/isopropanol 50/50; flow rate: 0.7 mL/min; t_{major} = 34.85 min, t_{minor} =83.52 min; ee = 96%; $[\alpha]^{20}_{D}$ = +204.5 (*c* 1.0, $CHCl_3$)].

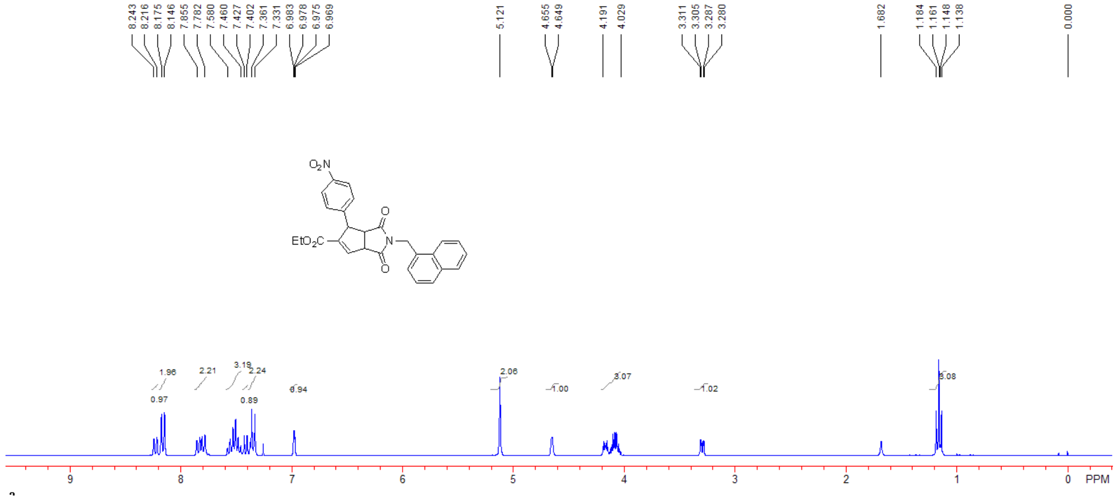

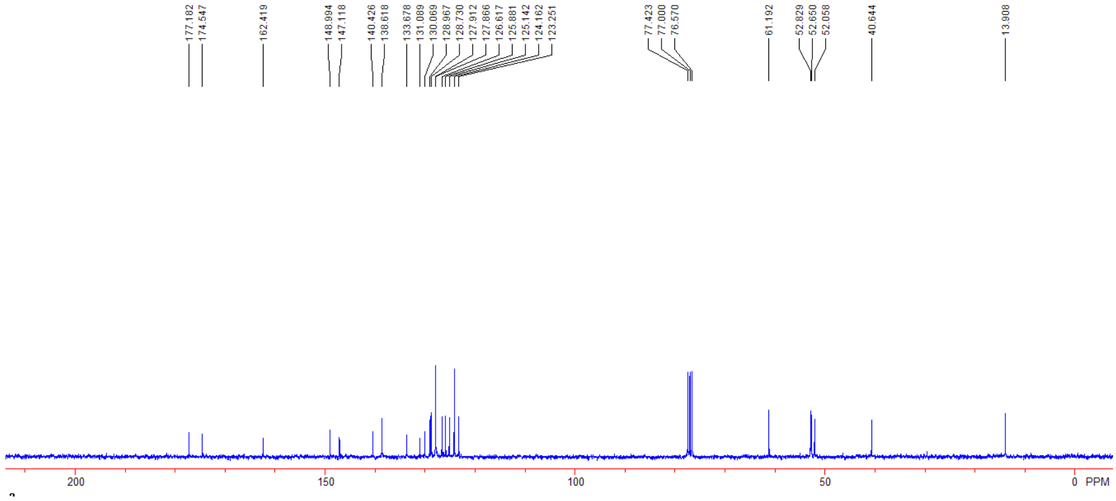

192 DHP-17-72-3+- PC-2 55 214 0.7

Sample Name:	**DHP-17-72-3+- PC-2 55 214 0.7**	*Injection Volume:*	**5.0**
Vial Number:	**RA1**	*Channel:*	**UV_VIS_1**
Sample Type:	**unknown**	*Wavelength:*	**214**
Control Program:	**WXL**	*Bandwidth:*	**n.a.**
Quantif. Method:	**WXL**	*Dilution Factor:*	**1.0000**
Recording Time:	**2011-8-19 11:02**	*Sample Weight:*	**1.0000**
Run Time (min):	**116.57**	*Sample Amount:*	**1.0000**

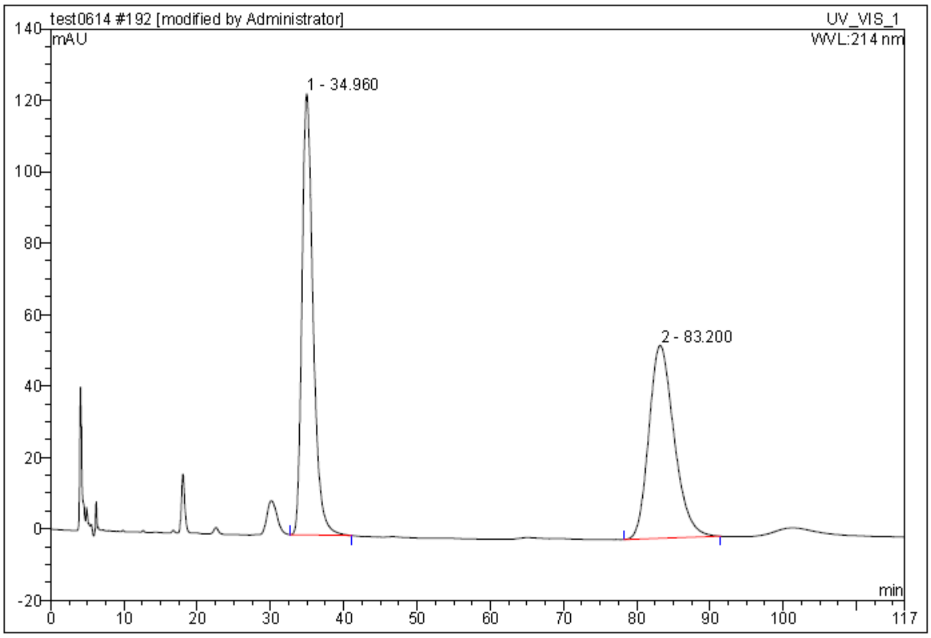

No.	Ret.Time min	Peak Name	Height mAU	Area mAU*min	Rel.Area %	Amount	Type
1	34.96	n.a.	123.529	217.480	50.18	n.a.	BMB
2	83.20	n.a.	54.025	215.933	49.82	n.a.	BMB
**Total:**			177.554	433.413	100.00	0.000	

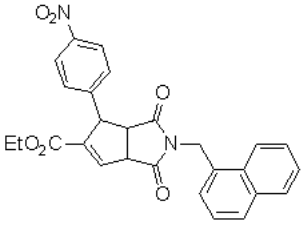

rac

PC-2, 214 nm
Hexane:^{i}PrOH = 50:50
0.7 mL/min

default/Integration

Chromeleon (c) Dionex 1996-2006
Version 6.80 SR10 Build 2818 (166959)

193 DHP-17-72-3

Sample Name:	**DHP-17-72-3**	*Injection Volume:*	**5.0**
Vial Number:	**RA2**	*Channel:*	**UV_VIS_1**
Sample Type:	**unknown**	*Wavelength:*	**214**
Control Program:	**WXL**	*Bandwidth:*	**n.a.**
Quantif. Method:	**WXL**	*Dilution Factor:*	**1.0000**
Recording Time:	**2011-8-19 13:00**	*Sample Weight:*	**1.0000**
Run Time (min):	**115.18**	*Sample Amount:*	**1.0000**

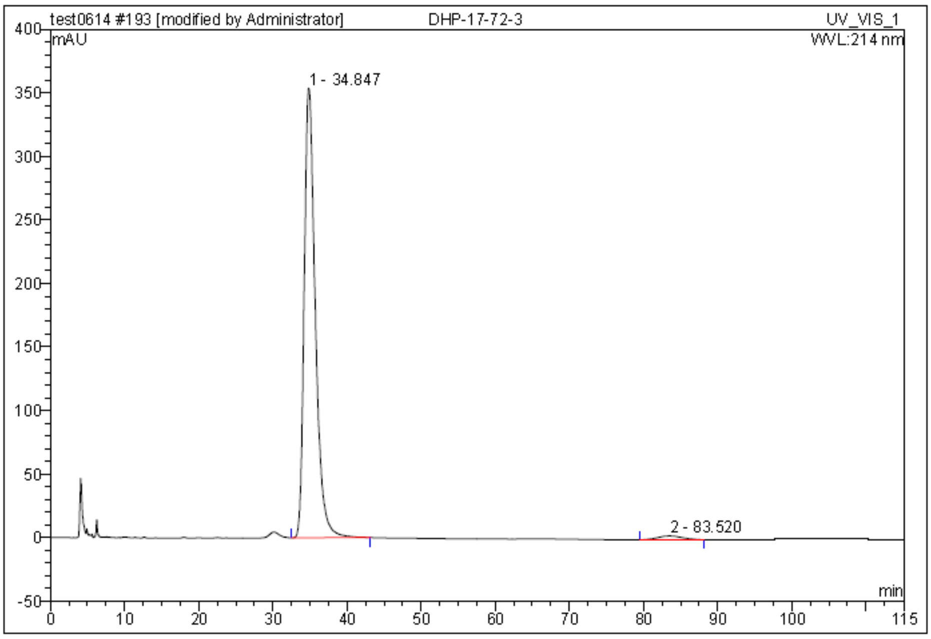

No.	Ret.Time min	Peak Name	Height mAU	Area mAU*min	Rel.Area %	Amount	Type
1	34.85	n.a.	353.746	613.639	98.18	n.a.	BMB*
2	83.52	n.a.	3.027	11.382	1.82	n.a.	BMB*
**Total:**			356.773	625.022	100.00	0.000	

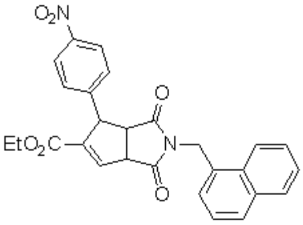

asy

PC-2, 214 nm
Hexane:^{i}PrOH = 50:50
0.7 mL/min

default/Integration

Chromeleon (c) Dionex 1996-2006
Version 6.80 SR10 Build 2818 (166959)

Chiral HPLC report: Enantiomeric excess was determined by HPLC with a Chiralcel PC-2 column [λ = 214 nm; eluent: hexane/isopropanol 50/50; flow rate: 0.7 mL/min; t_{major} = 34.85 min, t_{minor} = 83.52 min; ee = 96%; $[\alpha]^{20}_D$ = +204.5 (*c* 1.0, $CHCl_3$)].

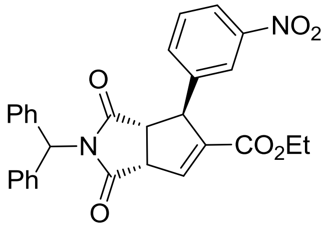

(3a*R*,4*S*,6a*S*)-ethyl 2-benzhydryl-4-(3-nitrophenyl)-1,3-dioxo-1,2,3,3a,4,6a-hexahydrocyclopenta[*c*]pyrrole-5-carboxylate (3f). Yield: 43 mg, 87%; yellow oil; IR (CH_2Cl_2): ν 3064, 3030, 2981, 2931, 1777, 1712, 1633, 1600, 1584, 1530, 1496, 1477, 1449, 1352, 1271, 1190, 1171, 1095, 1031, 989, 892, 873, 858, 808, 737, 700 cm^{-1}; ^1H NMR (300 MHz, $CDCl_3$, TMS): δ 1.18 (3H, t, J = 6.9 Hz, CH_3), 3.39 (1H, dd, J = 2.4, 7.5 Hz, CH), 4.06–4.17 (2H, m, CH_2), 4.23–4.27 (1H, m, CH), 4.69 (1H, d, J = 0.9 Hz, CH), 6.52 (1H, s, CH), 7.01 (1H, d, J = 0.9 Hz, CH), 7.23–7.35 (10H, m, ArH), 7.49–7.54 (1H, m, ArH), 7.59 (1H, d, J = 7.5 Hz, ArH), 8.00 (1H, s, ArH), 8.12 (1H, d, J = 8.1 Hz, ArH); ^{13}C NMR (75 MHz, $CDCl_3$, TMS): δ 13.9, 52.1, 52.8, 58.8, 61.2, 121.6, 122.6, 128.0, 128.4, 128.5, 129.9, 133.6, 136.9, 138.8, 140.5, 143.8, 148.6, 162.5, 174.2, 177.0; MS (ESI) *m/z* (%): 497.0 (100) [M^+ + 1]; HRMS (ESI) Calcd. for $C_{29}H_{24}N_2NaO_6$ (M^+ + Na) requires 519.1527, found: 519.1509; Enantiomeric excess was determined by HPLC with a Chiralcel IC-H column [λ = 214 nm; eluent: hexane/isopropanol 80/20; flow rate: 0.7 mL/min; t_{major} = 38.82 min, t_{minor} = 60.56 min; ee = 94%; $[\alpha]^{20}_D$ = +248.7 (*c* 1.0, $CHCl_3$)].

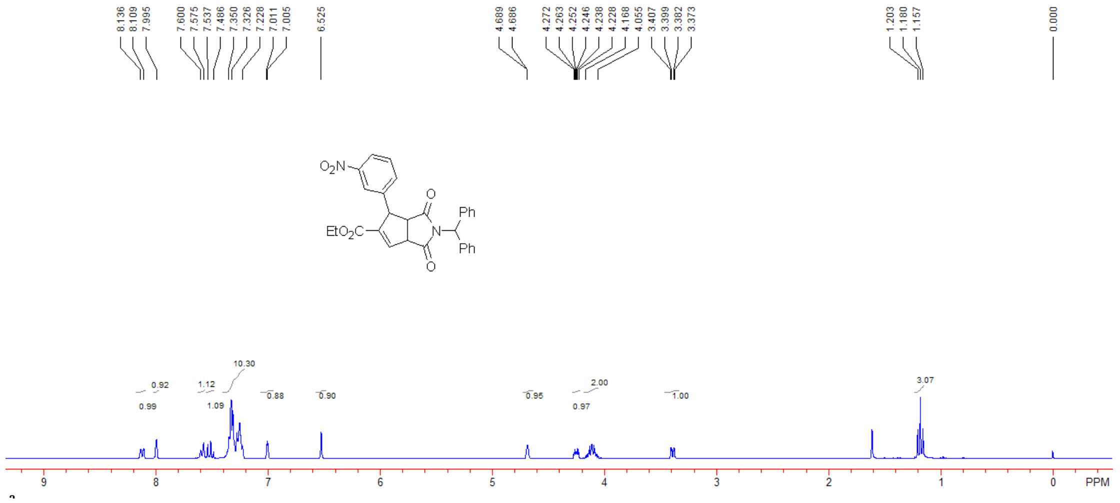

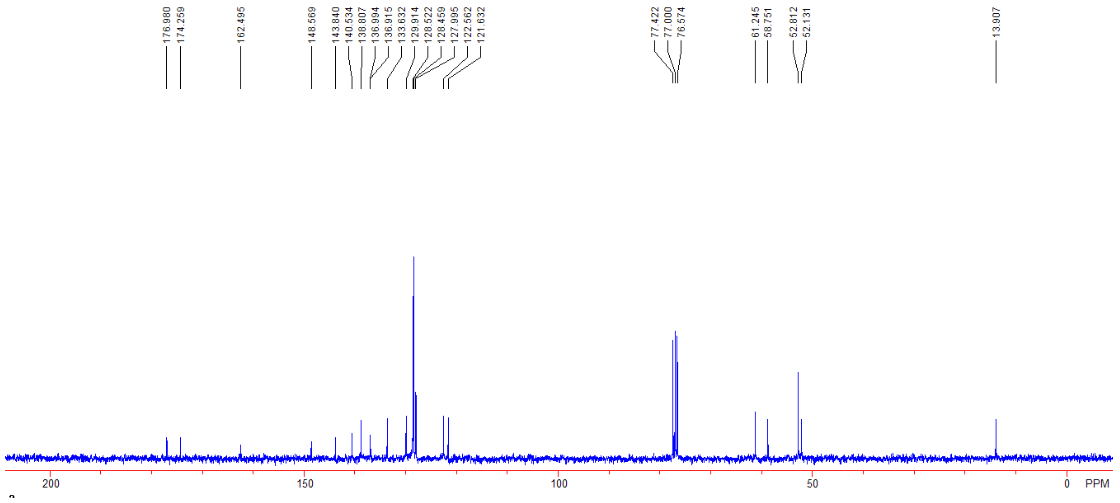

N2000 数据工作站 1

实验时间：2011-08-22,12:05:38
谱图文件:E:\实验数据\HPLC\dhp\dhp-17-77-N-CHPh2-IC-80-20-0.7-214-rac.org

实验者：
报告时间：2011-11-18,21:57:43
积分方法:面积归一法

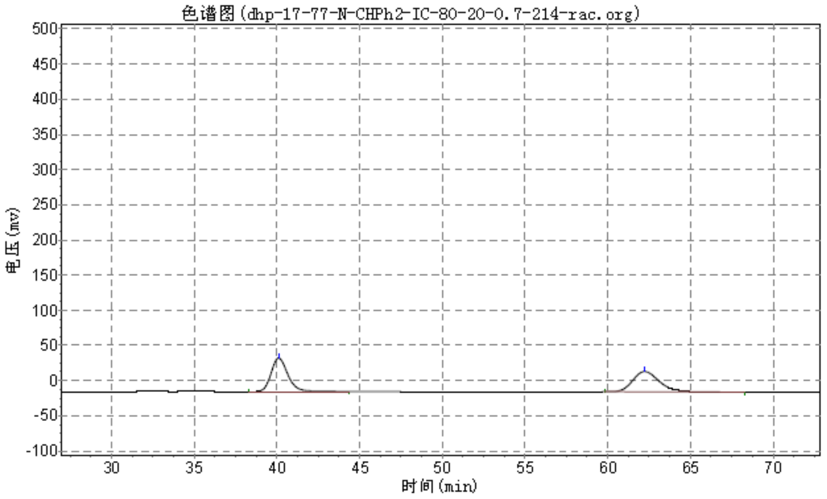

分析结果表

峰号	峰名	保留时间	峰高	峰面积	含量
1		40.108	47517.902	3314779.000	50.6086
2		62.227	28911.902	3235059.500	49.3914
**总计**			76429.805	6549838.500	100.0000

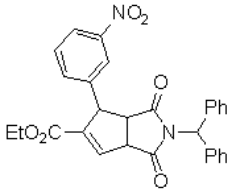

rac

IC-H, 214 nm
Hexane:^{i}PrOH = 80:20
0.7 mL/min

实验时间：2011-08-22,13:34:22
谱图文件:E:\实验数据\HPLC\dhp\dhp-17-77-N-CHPh2-IC-80-20-0.7-214-asy.org

实验者：
报告时间：2011-11-18,21:59:13
积分方法:面积归一法

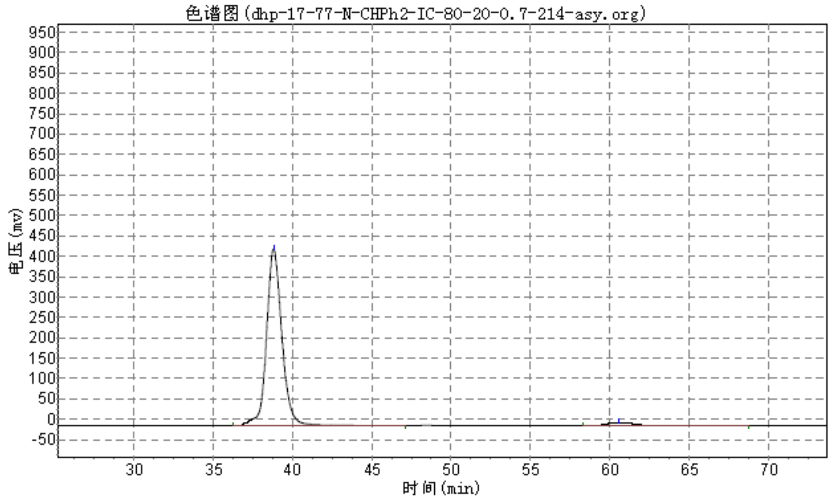

分析结果表

峰号	峰名	保留时间	峰高	峰面积	含量
1		38.817	432819.719	28980274.000	96.9618
2		60.562	7944.897	908075.500	3.0382
**总计**			440764.616	29888349.500	100.0000

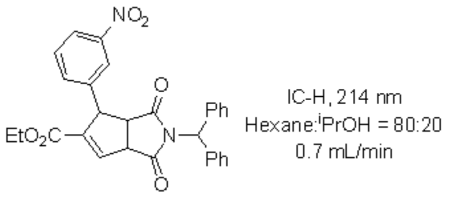

asy

Chiral HPLC report: Enantiomeric excess was determined by HPLC with a Chiralcel IC-H column [λ = 214 nm; eluent: hexane/isopropanol 80/20; flow rate: 0.7 mL/min; t_{major} = 38.82 min, t_{minor} = 60.56 min; ee = 94%; $[\alpha]^{20}_{D}$ = +248.7 (c 1.0, $CHCl_3$)].

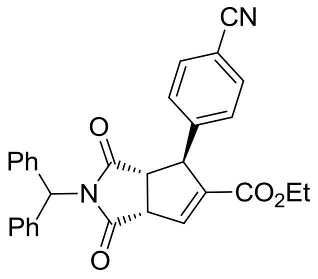

(3a*R*,4*S*,6a*S*)-ethyl 2-benzhydryl-4-(4-cyanophenyl)-1,3-dioxo-1,2,3,3a,4,6a-hexahydrocyclopenta[*c*]pyrrole-5-carboxylate (3g). Yield: 40 mg, 84%; colorless oil; IR (CH_2Cl_2): ν 3030, 2982, 2228, 1776, 1712, 1634, 1607, 1496, 1449, 1348, 1355, 1327, 1271, 1189, 1170, 1094, 1019, 987, 919, 843, 807, 739, 700 cm^{-1}; ^1H NMR (300 MHz, $CDCl_3$, TMS): δ 1.17 (3H, t, J = 7.2 Hz, CH_3), 3.34 (1H, dd, J = 2.4, 7.5 Hz, CH), 4.05–4.20 (3H, m, CH+CH_2), 4.62 (1H, s, CH), 6.52 (1H, s, CH), 6.98 (1H, d, J = 1.8, 2.7 Hz, CH), 7.22–7.34 (12H, m, ArH), 7.62 (2H, d, J = 8.1 Hz, ArH); ^{13}C NMR (75 MHz, $CDCl_3$, TMS): δ 13.9, 52.0, 52.8, 53.1, 58.7, 61.2, 114.4, 118.4, 127.8, 128.0, 128.4, 128.5, 132.7, 136.9, 137.0, 138.6, 140.5, 147.0, 162.4, 174.3, 177.0; MS (ESI) *m/z* (%): 477.0 (100) $[M^{+}+1]$; HRMS (ESI) Calcd. for $C_{30}H_{24}N_2NaO_4$ (M^+ + Na) requires 499.1628, found: 499.1611; Enantiomeric excess was determined by HPLC with a Chiralcel IC-H column [λ = 214 nm; eluent: hexane/isopropanol 50/50; flow rate: 0.7 mL/min; t_{major} = 16.50 min, t_{minor} = 19.33 min; ee = 96%; $[\alpha]^{20}_{D}$ = +299.1 (c 1.0, $CHCl_3$)].

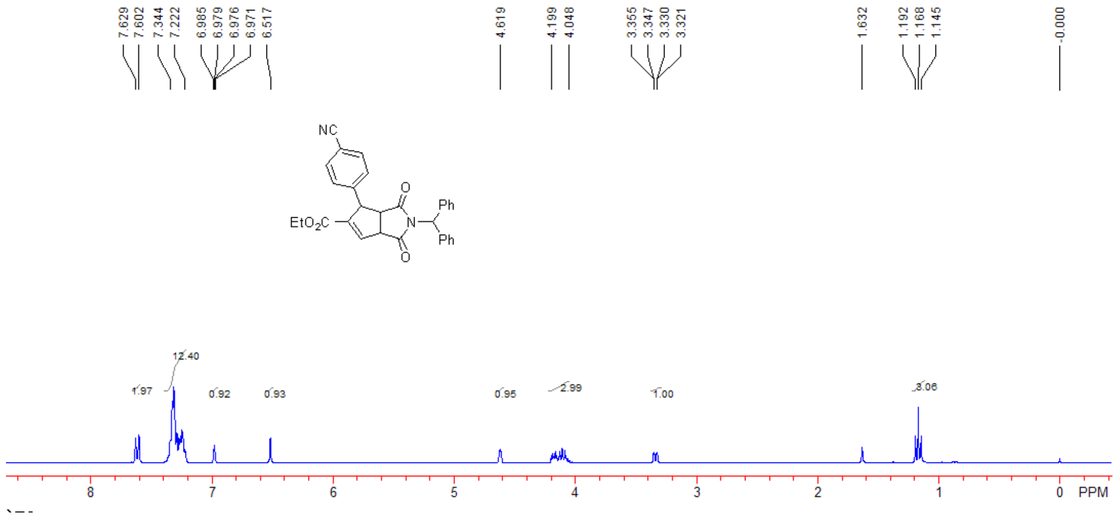

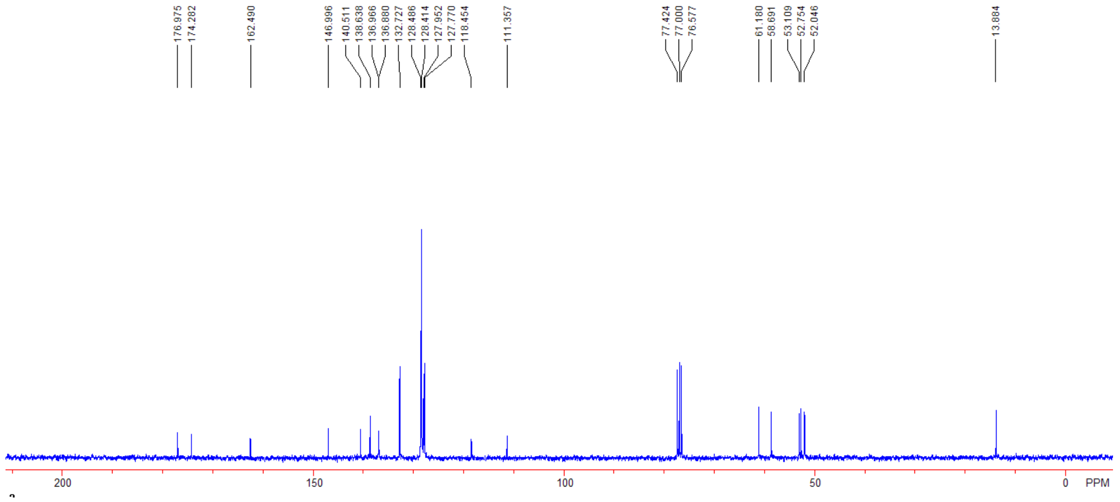

实验时间：2011-08-21,19:13:36
谱图文件:E:\实验数据\HPLC\dhp\dhp-17-78-N-CHPh2-IC-50-50-0.7-214-rac.org

实验者：
报告时间：2011-11-18,21:59:42
积分方法:面积归一法

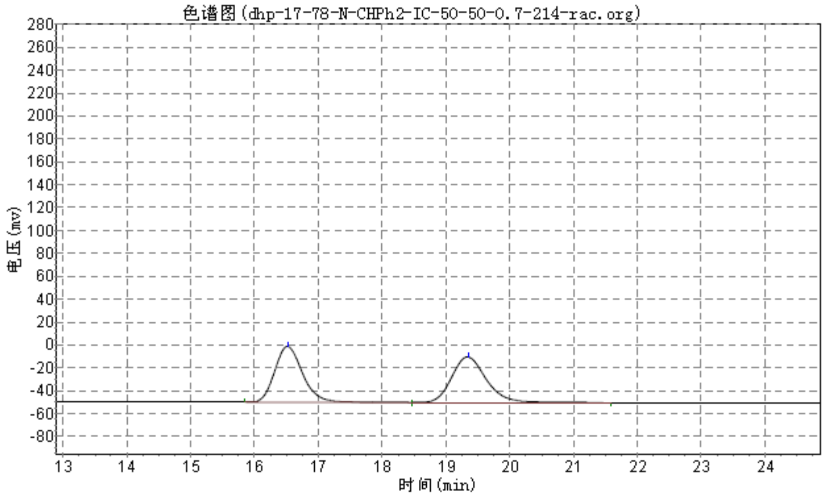

分析结果表

峰号	峰名	保留时间	峰高	峰面积	含量
1		16.523	48836.254	1495605.000	50.0133
2		19.357	39720.594	1494810.125	49.9867
**总计**			88556.848	2990415.125	100.0000

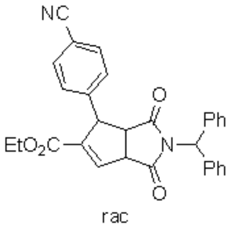

IC-H, 214 nm
Hexane:^{i}PrOH = 50:50
0.7 mL/min

N2000 数据工作站 1

实验时间：2011-08-21,19:53:53
谱图文件:E:\实验数据\HPLC\dhp\dhp-17-78-N-CHPh2-IC-50-50-0.7-214-asy.org

实验者：
报告时间：2011-11-18,22:00:34
积分方法:面积归一法

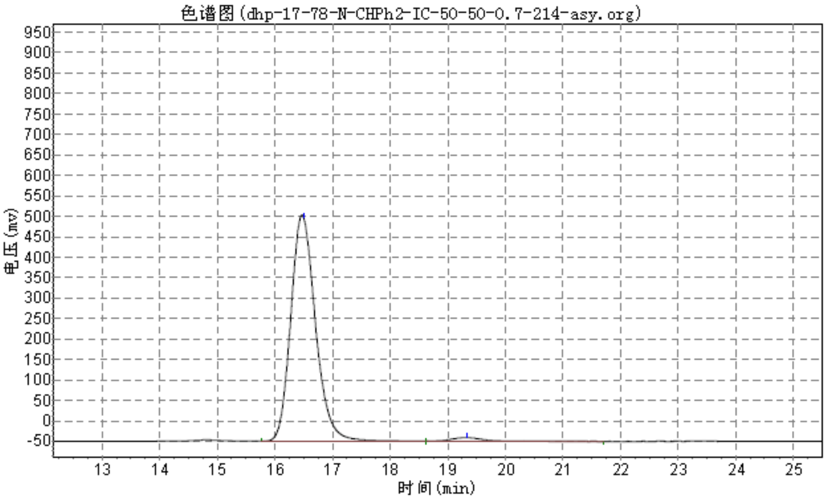

分析结果表

峰号	峰名	保留时间	峰高	峰面积	含量
1		16.498	550996.250	16559629.000	97.8055
2		19.332	9099.071	371545.938	2.1944
**总计**			560095.321	16931174.938	100.0000

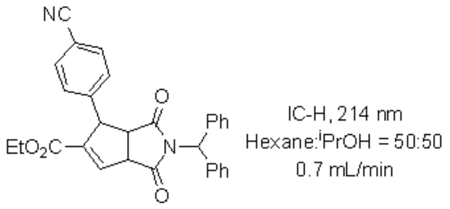

asy

Chiral HPLC report: Enantiomeric excess was determined by HPLC with a Chiralcel IC-H column [λ = 214 nm; eluent: hexane/isopropanol 50/50; flow rate: 0.7 mL/min; t_{major} = 16.50 min, t_{minor} = 19.33 min; ee = 96%; $[\alpha]^{20}_{D}$ = +299.1 (*c* 1.0, $CHCl_3$)].

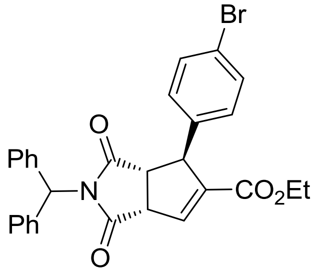

(3a*R*,4*S*,6a*S*)-ethyl 2-benzhydryl-4-(4-bromophenyl)-1,3-dioxo-1,2,3,3a,4,6a-hexahydrocyclo-penta[*c*]pyrrole-5-carboxylate (3h). Yield: 39 mg, 74%; colorless oil; IR (CH_2Cl_2): ν 3029, 2980,

1775, 1712, 1635, 1488, 1448, 1383, 1354, 1270, 1190, 1170, 1093, 1011, 987, 918, 877, 734, 698 cm^{-1}; ^{1}H NMR (400 MHz, $CDCl_3$, TMS): δ 1.17 (3H, t, J = 7.2 Hz, CH_3), 3.33 (1H, d, J = 7.2 Hz, CH), 4.07–4.16 (3H, m, CH+CH_2), 4.54 (1H, s, CH), 6.51 (1H, s, CH), 6.92 (1H, s, CH), 7.05 (2H, d, J = 8.4 Hz, ArH), 7.22–7.32 (10H, m, ArH), 7.43 (2H, d, J = 8.4 Hz, ArH); ^{13}C NMR (100 MHz, $CDCl_3$, TMS): δ 13.9, 52.4, 52.68, 52.75, 58.6, 61.1, 121.2, 127.9, 128.46, 128.49, 128.6, 132.0, 137.0, 137.9, 140.7, 141.2, 162.8, 174.6, 177.3; MS (ESI) m/z (%): 530.0 (100) [M^+ + 1]; HRMS (MALDI) Calcd. for $C_{29}H_{24}NO_4BrNa$ (M^+ + Na) requires 552.0781, found: 552.0798; Enantiomeric excess was determined by HPLC with a Chiralcel IC-H column [λ = 214 nm; eluent: hexane/isopropanol 50/50; flow rate: 0.7 mL/min; t_{major} = 9.06 min, t_{minor} = 10.38 min; ee = 97%; $[\alpha]^{20}_D$ = +240.9 (c 1.0, $CHCl_3$)].

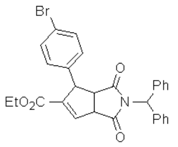

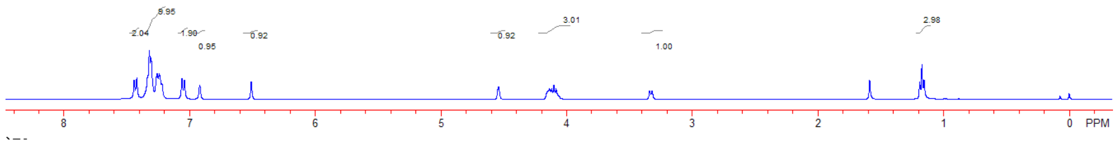

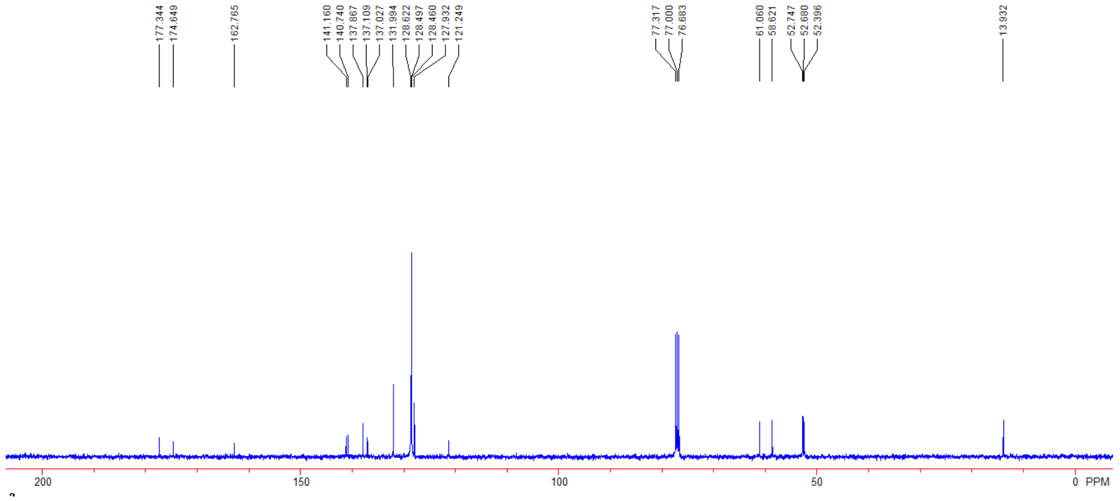

实验时间：2011-08-29,15:22:55
谱图文件:E:\实验数据\HPLC\dhp\dhp-17-84-N-CHPh2-IC-50-50-0.7-214-rac.org

实验者：
报告时间：2011-11-18,22:01:14
积分方法:面积归一法

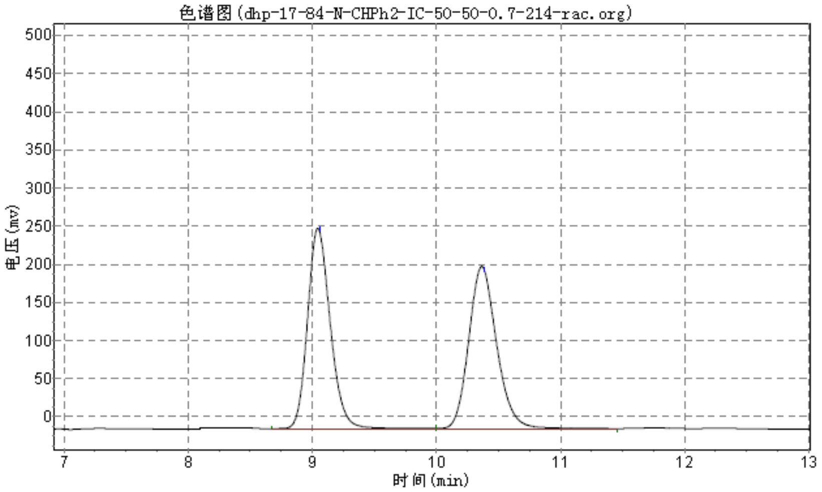

分析结果表

峰号	峰名	保留时间	峰高	峰面积	含量
1		9.057	261156.891	3332328.750	50.0473
2		10.390	210492.000	3326024.250	49.9527
**总计**			471648.891	6658353.000	100.0000

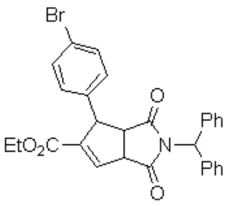

rac

IC-H, 214 nm
Hexane:^{i}PrOH = 50:50
0.7 mL/min

N2000 数据工作站 1

实验时间：2011-08-29,16:10:37
谱图文件:E:\实验数据\HPLC\dhp\dhp-17-84-N-CHPh2-IC-50-50-0.7-214-asy.org

实验者：
报告时间：2011-11-18,22:02:33
积分方法:面积归一法

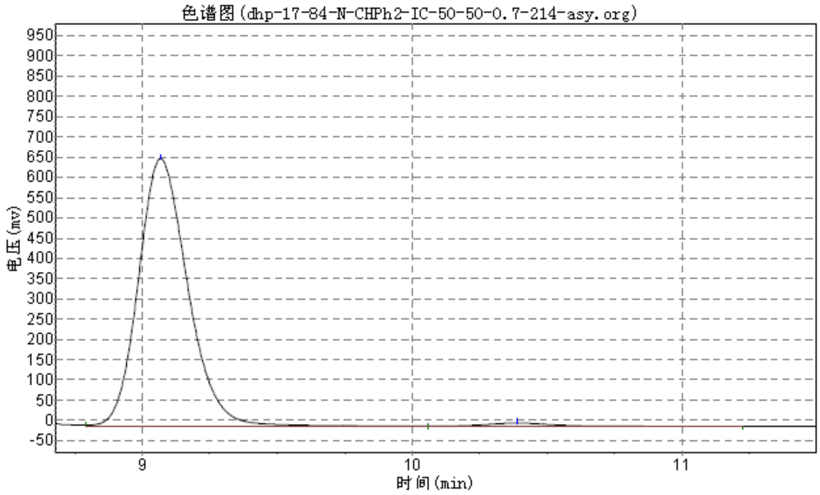

分析结果表

峰号	峰名	保留时间	峰高	峰面积	含量
1		9.067	661049.063	8379444.000	98.4997
2		10.388	7945.128	127628.711	1.5003
**总计**			668994.191	8507072.711	100.0000

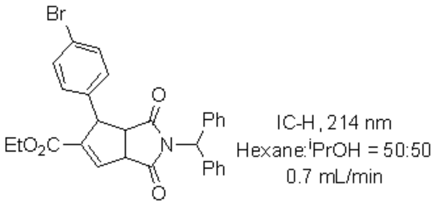

asy

Chiral HPLC report: Enantiomeric excess was determined by HPLC with a Chiralcel IC-H column [λ = 214 nm; eluent: hexane/isopropanol 50/50; flow rate: 0.7 mL/min; t_{major} = 9.06 min, t_{minor} = 10.38 min; ee = 97%; $[\alpha]^{20}_{D}$ = +240.9 (*c* 1.0, $CHCl_3$)].

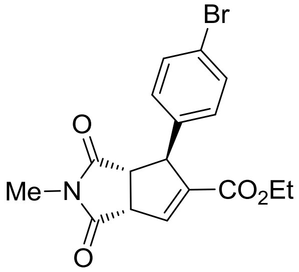

(3a*R*,4*S*,6a*S*)-ethyl 4-(4-bromophenyl)-2-methyl-1,3-dioxo-1,2,3,3a,4,6a-hexahydrocyclo-penta[*c*]pyrrole-5-carboxylate (3i). Yield: 30 mg, 79%; colorless oil; IR (CH_2Cl_2): ν 2981, 1781,

1714, 1653, 1634, 1558, 1540, 1521, 1506, 1488, 1433, 1409, 1378, 1327, 1272, 1202, 1127, 1095, 1071,1011, 960, 891, 874, 828, 790, 740, 715 cm^{-1}; ^{1}H NMR (400 MHz, $CDCl_3$, TMS): δ 1.17 (3H, t, J = 7.2 Hz, CH_3), 3.00 (3H, s, CH_3), 3.32 (1H, dd, J = 1.6, 6.8 Hz, CH), 4.04–4.16 (3H, m, CH+CH_2), 4.50 (1H, s, CH), 6.89–6.90 (1H, m, CH), 7.08–7.10 (2H, m, ArH), 7.44–7.46 (2H, m, ArH); ^{13}C NMR (100 MHz, $CDCl_3$, TMS): δ 13.9, 25.2, 52.4, 52.5, 52.9, 61.0, 121.2, 128.7, 132.0, 137.7, 140.8, 141.0, 162.7, 175.2, 177.7; MS (ESI) *m/z* (%): 378.0 (100) [M^+ + 1]; HRMS (MALDI) Calcd. for $C_{17}H_{16}NO_4BrNa$ (M^+ + Na) requires 400.0155, found: 400.0141; Enantiomeric excess was determined by HPLC with a Chiralcel IC-H column [λ = 214 nm; eluent: hexane/isopropanol 50/50; flow rate: 0.7 mL/min; t_{major} = 16.24 min, t_{minor} = 17.48 min; ee = 95%; $[\alpha]^{20}_{D}$ = +239.9 (c 1.0, $CHCl_3$)].

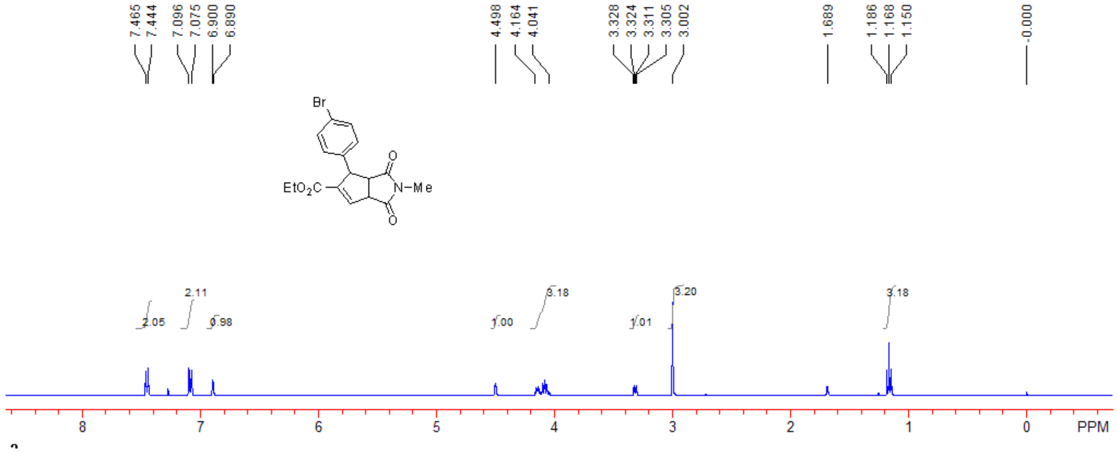

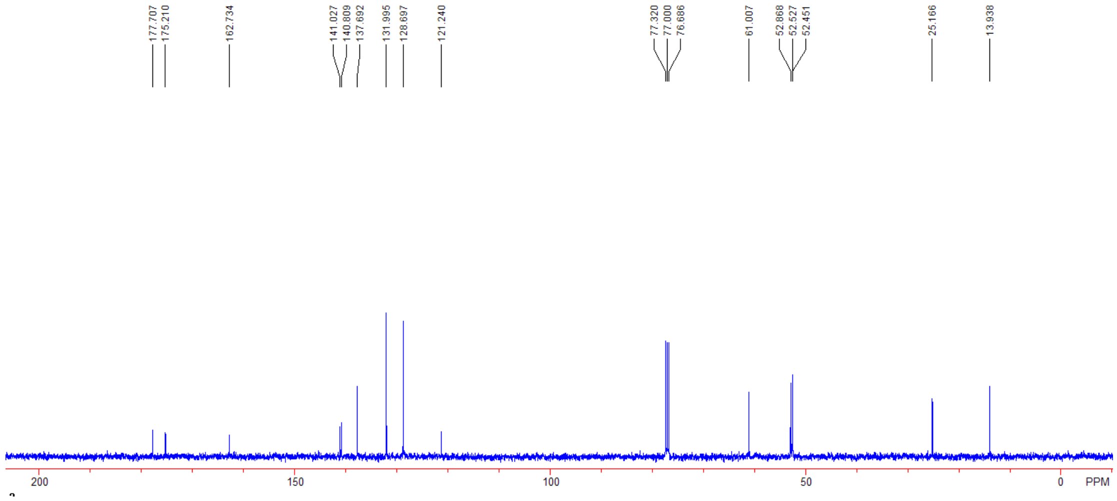

实验时间：2011-09-08,16:53:16
谱图文件:E:\实验数据\HPLC\dhp\dhp-18-4-N-Me-IC-50-50-0.7-214-rac.org

实验者：
报告时间：2011-11-18,22:15:21
积分方法:面积归一法

色谱图(dhp-18-4-N-Me-IC-50-50-0.7-214-rac.org)

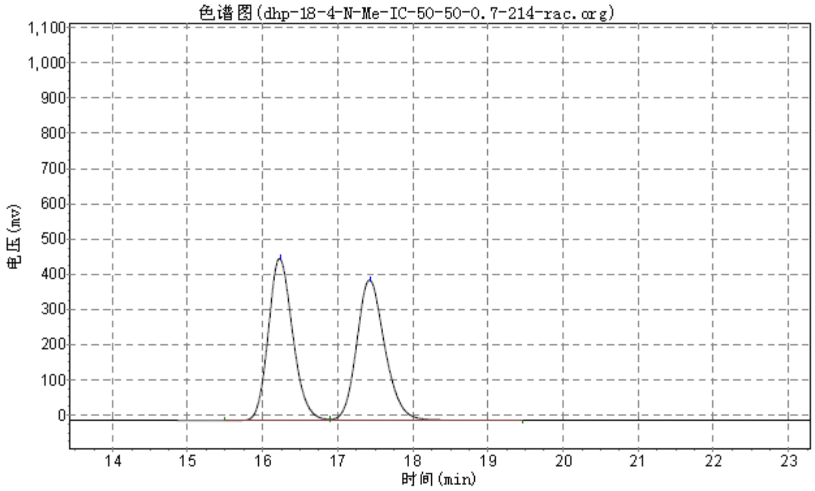

分析结果表

峰号	峰名	保留时间	峰高	峰面积	含量
1		16.232	459044.594	10378357.000	49.9012
2		17.432	398001.969	10419444.000	50.0988
**总计**			857046.563	20797801.000	100.0000

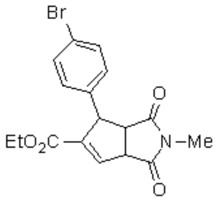

IC-H , 214 nm
Hexane:^{i}PrOH = 50:50
0.7 mL/min

rac

N2000 数据工作站 1

实验时间：2011-09-08,17:32:39
谱图文件:E:\实验数据\HPLC\dhp\dhp-18-4-N-Me-IC-50-50-0.7-214-asy.org

实验者：
报告时间：2011-11-18,22:16:28
积分方法:面积归一法

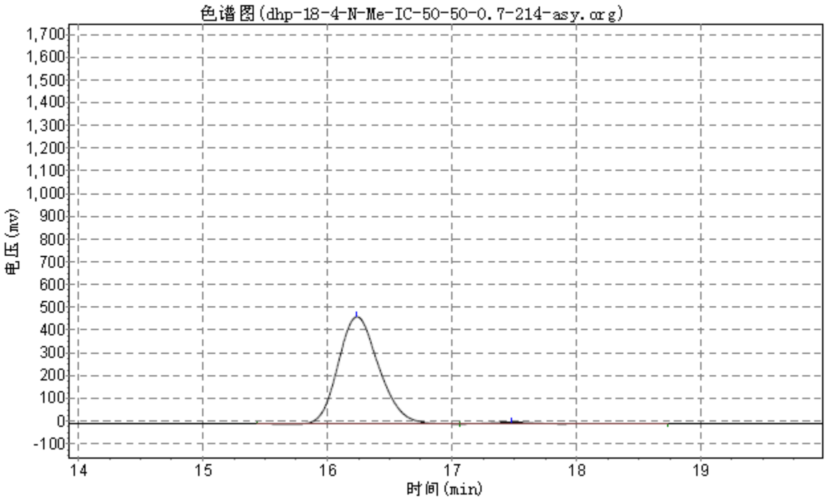

分析结果表

峰号	峰名	保留时间	峰高	峰面积	含量
1		16.238	472927.594	10805376.000	97.4862
2		17.477	9626.848	278624.625	2.5138
**总计**			482554.441	11084000.625	100.0000

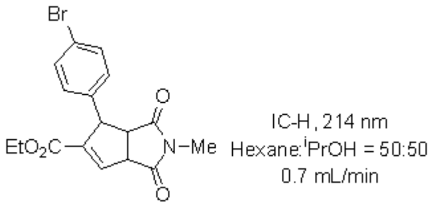

asy

Chiral HPLC report: Enantiomeric excess was determined by HPLC with a Chiralcel IC-H column [λ = 214 nm; eluent: hexane/isopropanol 50/50; flow rate: 0.7 mL/min; t_{major} = 16.24 min, t_{minor} = 17.48 min; ee = 95%; $[\alpha]^{20}_{D}$ = +239.9 (c 1.0, $CHCl_3$)].

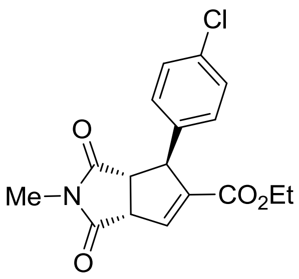

(3a*R*,4*S*,6a*S*)-ethyl 4-(4-chlorophenyl)-2-methyl-1,3-dioxo-1,2,3,3a,4,6a-hexahydrocyclo-penta[*c*]pyrrole-5-carboxylate (3j). Yield: 30 mg, 90%; colorless oil; IR (CH_2Cl_2): ν 2982, 1781,

1704, 1634, 1491, 1434, 1379, 1272, 1202, 1127, 1093, 1045, 1014, 1001, 959, 891, 874, 790, 751, 739, 666 cm^{-1}; ^{1}H NMR (400 MHz, $CDCl_3$, TMS): δ 1.17 (3H, t, J = 6.8 Hz, CH_3), 3.00 (3H, s, CH_3), 3.32 (1H, dd, J = 2.4, 7.6 Hz, CH), 4.04–4.17 (3H, m, CH+CH_2), 4.52 (1H, s, CH), 6.90 (1H, dd, J = 1.6, 2.4 Hz, CH), 7.14 (2H, d, J = 8.0 Hz, ArH), 7.30 (2H, d, J = 8.0 Hz, ArH); ^{13}C NMR (100 MHz, $CDCl_3$, TMS): δ 13.9, 25.2, 52.4, 52.6, 52.9, 61.0, 128.3, 129.0, 133.2, 137.6, 140.3, 141.1, 162.8, 175.2, 177.7; MS (ESI) m/z (%): 334.0 (100) [M^+ + 1]; HRMS (MALDI) Calcd. for $C_{17}H_{16}NO_4ClNa$ (M^+ + Na) requires 356.0660, found: 356.0659; Enantiomeric excess was determined by HPLC with a Chiralcel IC-H column [λ = 214 nm; eluent: hexane/isopropanol 80/20; flow rate: 0.7 mL/min; t_{major} = 31.48 min, t_{minor} = 35.18 min; ee = 97%; $[\alpha]^{20}_{D}$ = +263.0 (c 1.0, $CHCl_3$)].

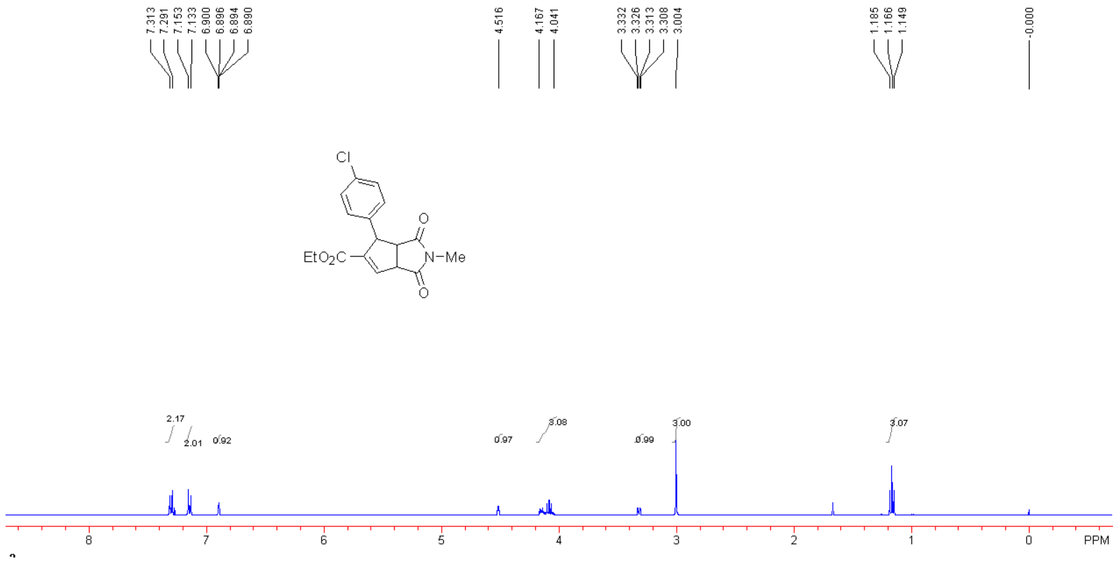

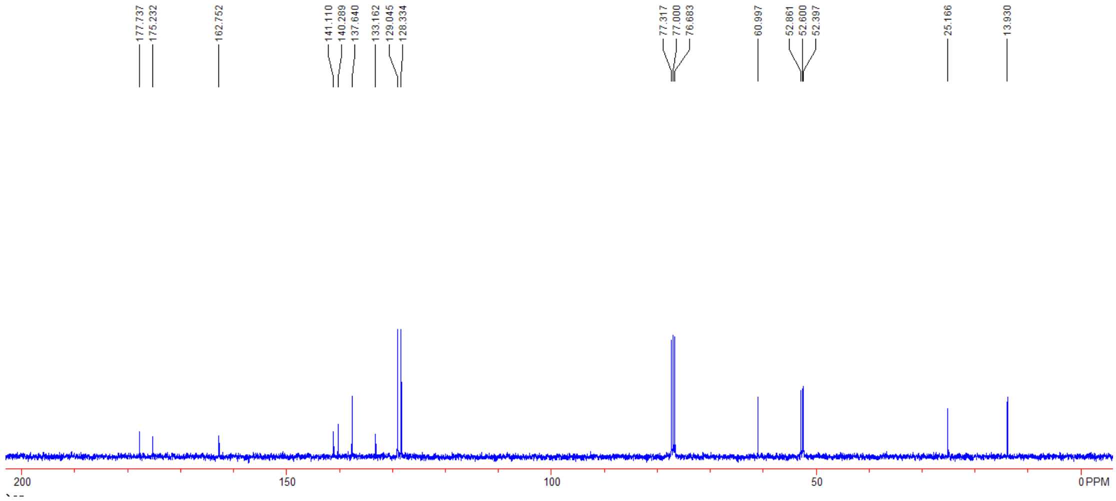

实验时间：2011-09-09，13:47:02
谱图文件:E:\实验数据\HPLC\dhp\dhp-18-16-N-Me-IC-80-20-0.7-214-rac.org

实验者：
报告时间：2011-11-18，22:07:53
积分方法:面积归一法

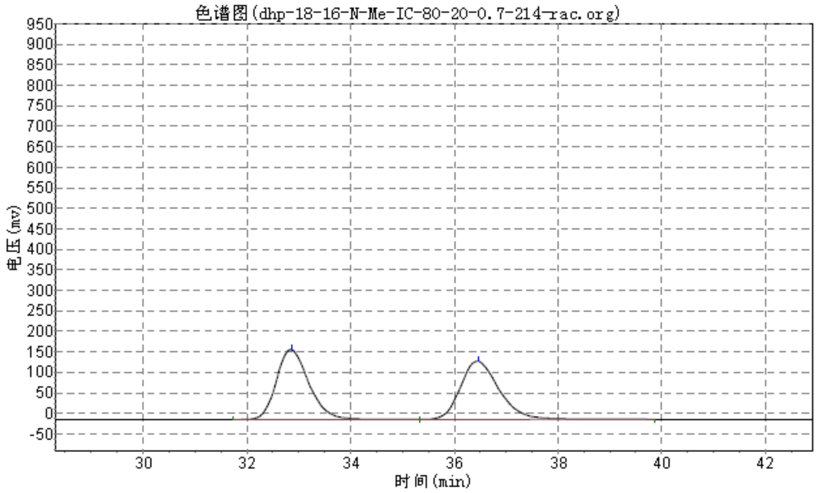

分析结果表

峰号	峰名	保留时间	峰高	峰面积	含量
1		32.865	169878.000	7370794.000	50.1006
2		36.465	141510.000	7341192.500	49.8994
**总计**			311388.000	14711986.500	100.0000

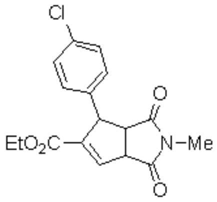

rac

IC-H, 214 nm
Hexane:^{i}PrOH = 80:20
0.7 mL/min

N2000 数据工作站 1

实验时间：2011-09-09,14:44:07
谱图文件:E:\实验数据\HPLC\dhp\dhp-18-16-N-Me-IC-80-20-0.7-214-asy.org

实验者：
报告时间：2011-11-18,22:09:22
积分方法:面积归一法

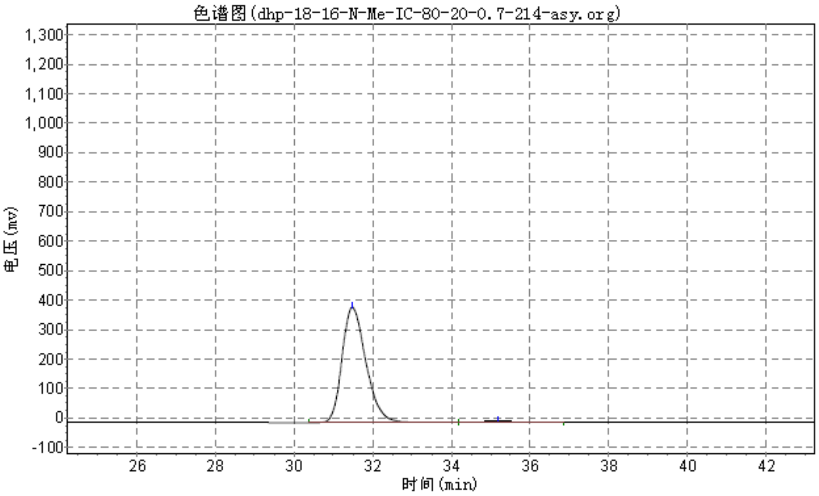

分析结果表

峰号	峰名	保留时间	峰高	峰面积	含量
1		31.480	391102.375	16476227.000	98.2510
2		35.177	5563.332	293290.656	1.7490
总计			396665.707	16769517.656	100.0000

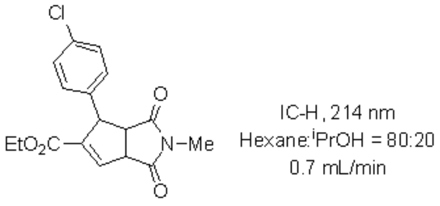

asy

Chiral HPLC report: Enantiomeric excess was determined by HPLC with a Chiralcel IC-H column [λ = 214 nm; eluent: hexane/isopropanol 80/20; flow rate: 0.7 mL/min; t_{major} = 31.48 min, t_{minor} = 35.18 min; ee = 97%; $[\alpha]^{20}_{D}$ = +263.0 (*c* 1.0, $CHCl_3$)].

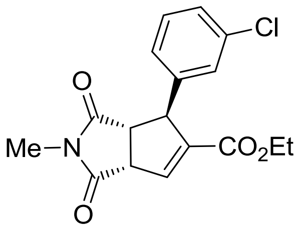

(3a*R*,4*S*,6a*S*)-ethyl 4-(3-chlorophenyl)-2-methyl-1,3-dioxo-1,2,3,3a,4,6a-hexahydrocyclo-penta[*c*]pyrrole-5-carboxylate (3k). Yield: 27 mg, 81%; colorless oil; IR (CH_2Cl_2): ν 2981, 2936,

1699, 1595, 1432, 1378, 1270, 1203, 1127, 1095, 1001, 960, 783, 765, 737, 699 cm^{-1}; ^{1}H NMR (400 MHz, $CDCl_3$, TMS): δ 1.17 (3H, t, *J* = 7.2 Hz, CH_3), 3.01 (3H, s, CH_3), 3.34 (1H, dd, *J* = 2.0, 7.2 Hz, CH), 4.05–4.19 (3H, m, CH+CH_2), 4.51 (1H, s, CH), 6.92 (1H, dd, *J* = 1.6, 2.4 Hz, CH), 7.12 (1H, dd, *J* = 1.6, 7.2 Hz, ArH), 7.17 (1H, s, ArH), 7.23–7.29 (2H, m, ArH); ^{13}C NMR (100 MHz, $CDCl_3$, TMS): δ 13.9, 25.2, 52.5, 52.6, 52.9, 61.0, 125.3, 127.0, 127.6, 130.2, 134.7, 137.9, 140.9, 143.7, 162.7, 175.2, 177.7; MS (MALDI) *m/z* (%): 379.2 (100) [M^+ + HCO_2H]; HRMS (MALDI) Calcd. for $C_{17}H_{17}NO_4Cl$ (M^+ + 1) requires 334.0841, found: 334.0847; Enantiomeric excess was determined by HPLC with a Chiralcel IC-H column [λ = 214 nm; eluent: hexane/isopropanol 80/20; flow rate: 0.7 mL/min; t_{major} = 55.36 min, t_{minor} = 70.69 min; ee = 97%; $[\alpha]^{20}_{D}$ = +316.0 (*c* 1.0, $CHCl_3$)].

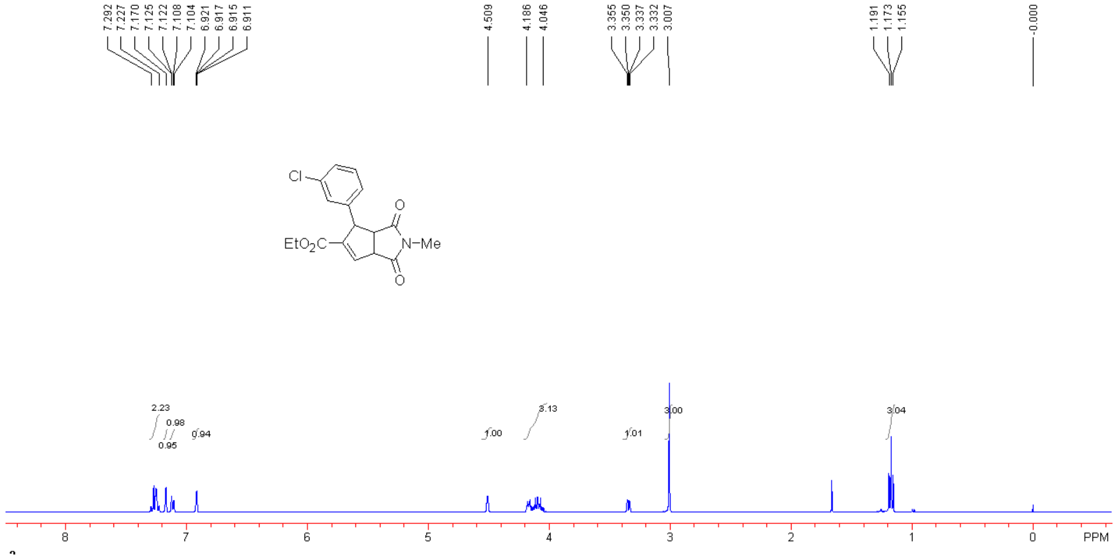

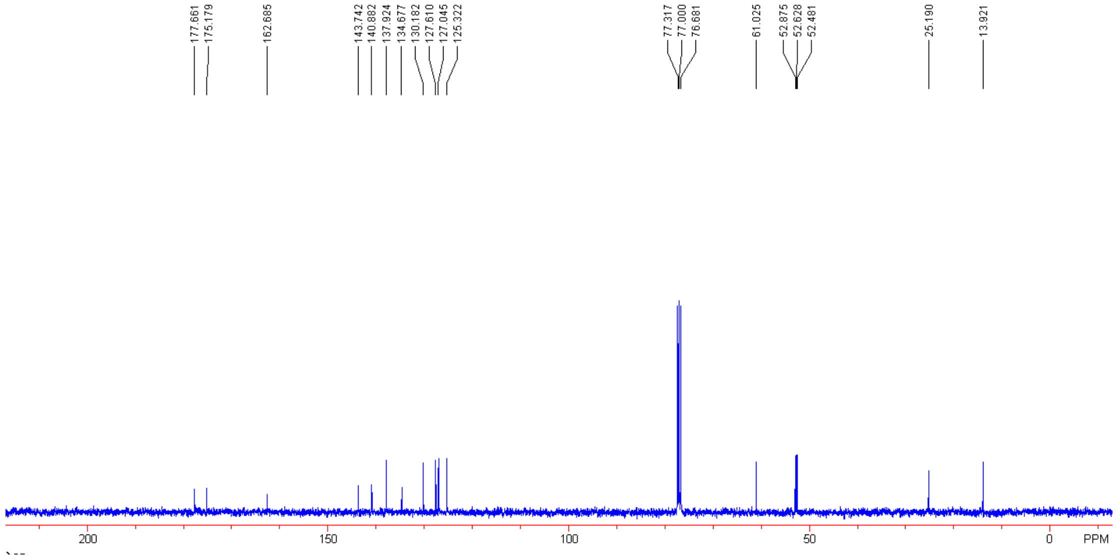

实验时间：2011-11-24,19:23:05
谱图文件:E:\实验数据\HPLC\dhp\dhp-19-15-IC-80-20-0.7-214-rac.org

实验者：
报告时间：2011-11-26,10:41:37
积分方法:面积归一法

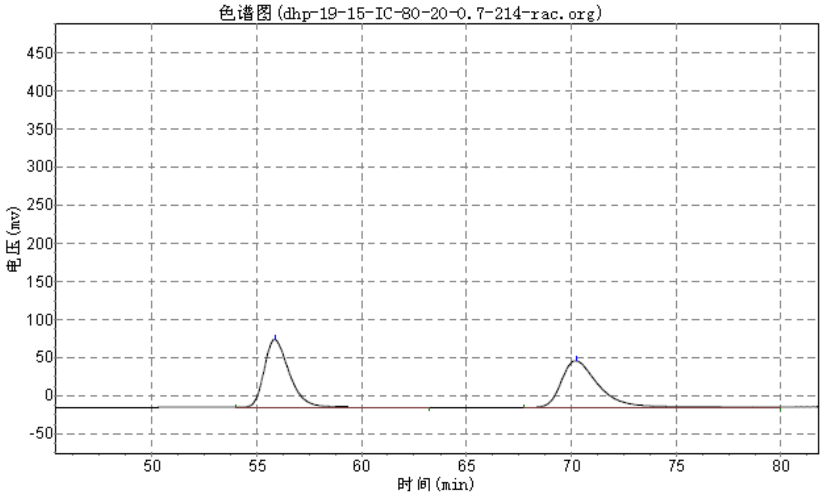

分析结果表

峰号	峰名	保留时间	峰高	峰面积	含量
1		55.865	88220.570	7376706.500	50.1241
2		70.265	60781.105	7340167.500	49.8759
总计			149001.676	14716874.000	100.0000

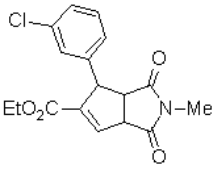

rac

IC-H, 214 nm
Hexane:^{i}PrOH = 80:20
0.7 mL/min

N2000 数据工作站 1

实验时间：2011-11-24, 20:49:22
谱图文件:E:\实验数据\HPLC\dhp\dhp-19-15-IC-80-20-0.7-214-asy.org

实验者：
报告时间：2011-11-26, 10:43:32
积分方法:面积归一法

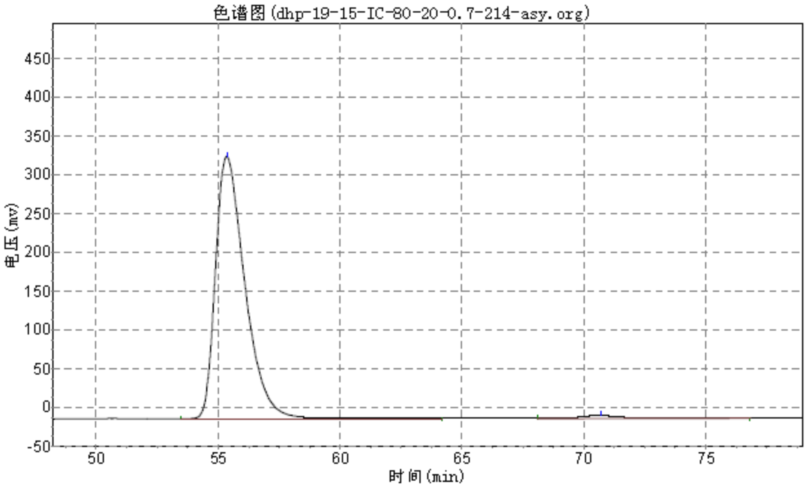

分析结果表

峰号	峰名	保留时间	峰高	峰面积	含量
1		55.365	337659.688	28361346.000	98.3254
2		70.698	3539.049	483030.406	1.6746
总计			341198.737	28844376.406	100.0000

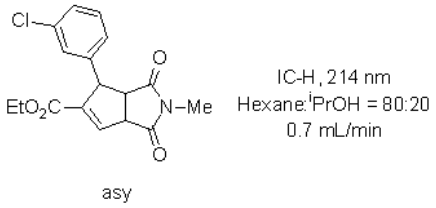

Chiral HPLC report: Enantiomeric excess was determined by HPLC with a Chiralcel IC-H column [λ = 214 nm; eluent: hexane/isopropanol 80/20; flow rate: 0.7 mL/min; t_{major} = 55.36 min, t_{minor} = 70.69 min; ee = 97%; $[\alpha]^{20}_{D}$ = +316.0 (*c* 1.0, $CHCl_3$)].

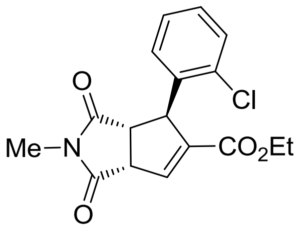

(3a*R*,4*R*,6a*S*)-ethyl 4-(2-chlorophenyl)-2-methyl-1,3-dioxo-1,2,3,3a,4,6a-hexahydrocyclo-penta[*c*]pyrrole-5-carboxylate (3l). Yield: 13 mg, 39%; colorless oil; IR (CH_2Cl_2): ν 2982, 1782,

1705, 1635, 1474, 1433, 1378, 1307, 1268, 1201, 1127, 1096, 1052, 1037, 1000, 959, 792, 758, 690 cm^{-1}; ^{1}H NMR (400 MHz, $CDCl_3$, TMS): δ 1.14 (3H, t, J = 7.2 Hz, CH_3), 3.01 (3H, s, CH_3), 3.37 (1H, d, J = 7.2 Hz, CH), 4.03–4.16 (3H, m, CH+CH_2), 5.02 (1H, s, CH), 6.97 (1H, dd, J = 1.6, 2.0 Hz, CH), 7.04–7.05 (1H, m, ArH), 7.20–7.22 (2H, m, ArH), 7.41–7.43 (1H, m, ArH); ^{13}C NMR (100 MHz, $CDCl_3$, TMS): δ 13.9, 25.2, 51.9, 53.2, 61.0, 127.1, 128.7, 130.3, 133.6, 138.6, 138.8, 140.5, 162.7, 175.2, 177.4; MS (ESI) m/z (%): 334.1 (100) [M^+ + 1]; HRMS (MALDI) Calcd. for $C_{17}H_{16}NO_4ClNa$ (M^+ + Na) requires 356.0660, found: 356.0651; Enantiomeric excess was determined by HPLC with a Chiralcel IC-H column [λ = 214 nm; eluent: hexane/isopropanol 80/20; flow rate: 0.7 mL/min; t_{major} = 42.76 min, t_{minor} = 48.89 min; ee = 73%; $[\alpha]^{20}_{D}$ = +245.6 (c 1.0, $CHCl_3$)].

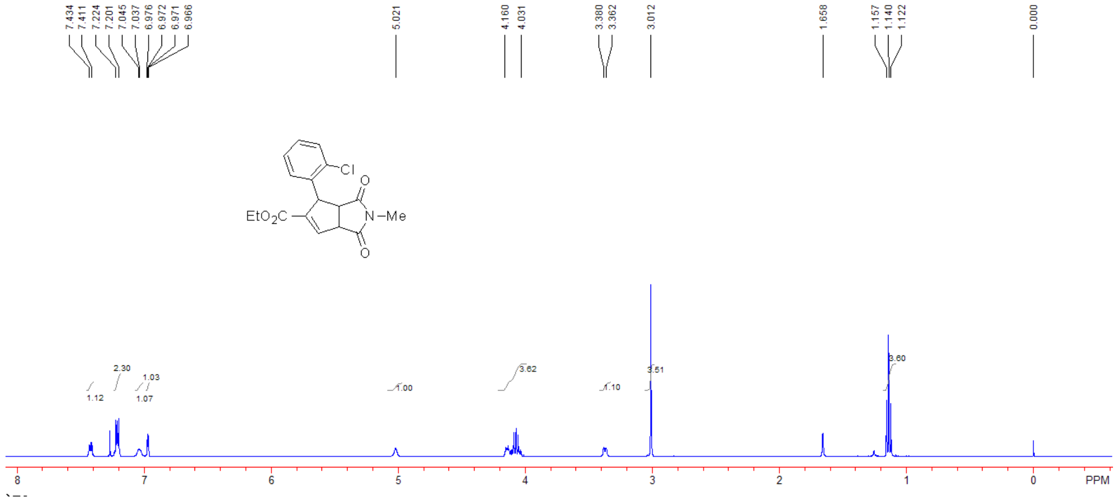

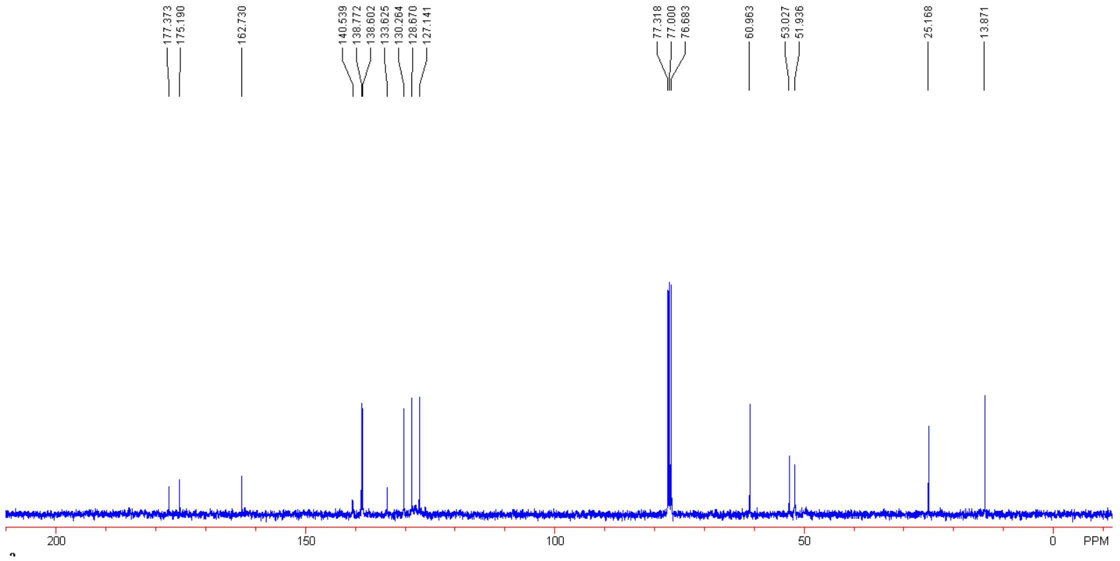

实验时间：2011-09-19,18:08:24
谱图文件:E:\实验数据\HPLC\dhp\dhp-18-26-N-Me-IC-80-20-0.7-214-rac.org

实验者：
报告时间：2011-11-18,22:17:07
积分方法:面积归一法

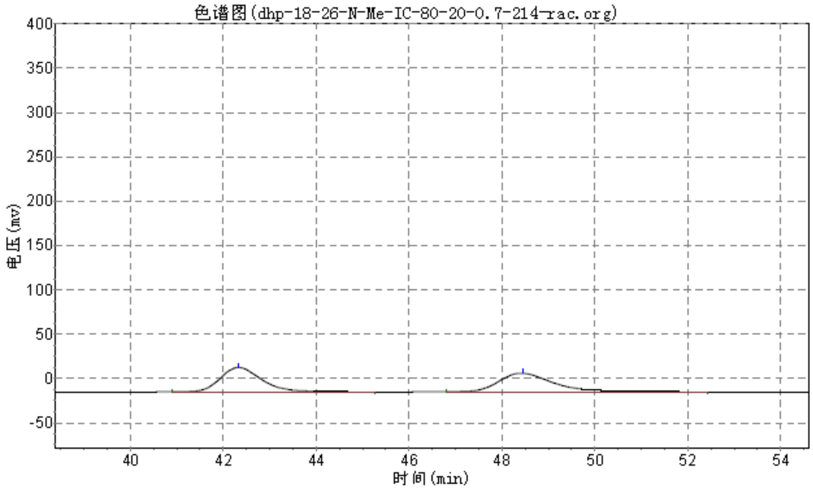

分析结果表

峰号	峰名	保留时间	峰高	峰面积	含量
1		42.332	27284.703	1654170.375	50.1206
2		48.448	20712.541	1646209.250	49.8794
总计			47997.244	3300379.625	100.0000

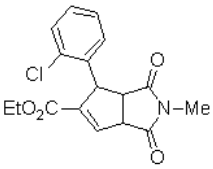

rac

IC-H, 214 nm
Hexane:^{i}PrOH = 80:20
0.7 mL/min

N2000 数据工作站 1

实验时间：2011-09-19, 19:16:19
谱图文件:E:\实验数据\HPLC\dhp\dhp-18-26-N-Me-IC-80-20-0.7-214-asy.org

实验者：
报告时间：2011-11-18, 22:17:38
积分方法:面积归一法

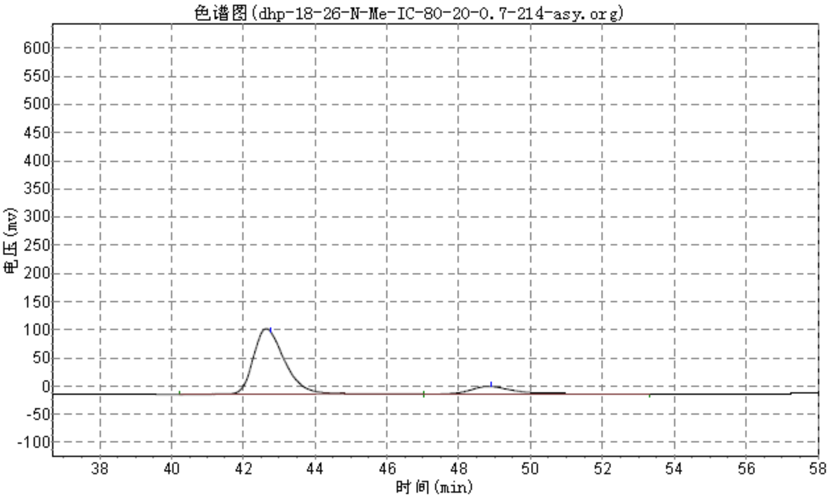

分析结果表

峰号	峰名	保留时间	峰高	峰面积	含量
1		42.765	114180.750	6967741.500	86.6999
2		48.898	12949.637	1068876.625	13.3001
总计			127130.387	8036618.125	100.0000

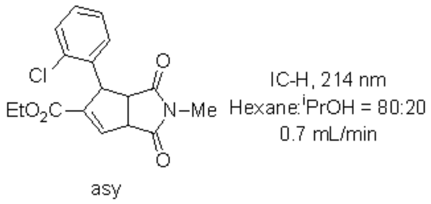

Chiral HPLC report: Enantiomeric excess was determined by HPLC with a Chiralcel IC-H column [λ = 214 nm; eluent: hexane/isopropanol 80/20; flow rate: 0.7 mL/min; t_{major} = 42.76 min, t_{minor} = 48.89 min; ee = 73%; $[\alpha]^{20}_{D}$ = +245.6 (*c* 1.0, $CHCl_3$)].

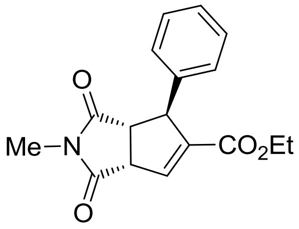

(3a*R*,4*S*,6a*S*)-ethyl 2-methyl-1,3-dioxo-4-phenyl-1,2,3,3a,4,6a-hexahydrocyclo-penta[*c*]pyrrole-5-carboxylate (3m). Yield: 22 mg, 74%; colorless oil; IR (CH_2Cl_2): ν 3029, 2981,

1781, 1705, 1634, 1601, 1558, 1540, 1506, 1495, 1454, 1433, 1379, 1329, 1272, 1202, 1127, 1095, 1045, 997, 959, 889, 872, 793, 761, 736, 700 cm^{-1}; ^{1}H NMR (400 MHz, $CDCl_3$, TMS): δ 1.15 (3H, t, *J* = 7.2 Hz, CH_3), 3.00 (3H, s, CH_3), 3.37 (1H, d, *J* = 7.6 Hz, CH), 4.01–4.17 (3H, m, CH+CH_2), 4.54 (1H, d, *J* = 1.6 Hz, CH), 6.89 (1H, dd, *J* = 1.6, 2.4 Hz, CH), 7.20 (2H, d, *J* = 8.0 Hz, ArH), 7.25–7.27 (1H, m, ArH), 7.31–7.35 (2H, m, ArH); ^{13}C NMR (100 MHz, $CDCl_3$, TMS): δ 13.9, 25.1, 52.8, 52.9, 53.0, 60.9, 126.9, 127.3, 128.9, 137.3, 141.5, 141.8, 163.0, 175.5, 178.0; MS (ESI) *m/z* (%): 300.1 (100) [M^+ + 1]; HRMS (MALDI) Calcd. for $C_{17}H_{17}NO_4Na$ (M^+ + Na) requires 322.1050, found: 322.1053; Enantiomeric excess was determined by HPLC with a Chiralcel IC-H column [λ = 214 nm; eluent: hexane/isopropanol 80/20; flow rate: 0.7 mL/min; t_{major} = 35.87 min, t_{minor} = 43.68 min; ee = 95%; $[\alpha]^{20}_{D}$ = +276.5 (*c* 1.0, $CHCl_3$)].

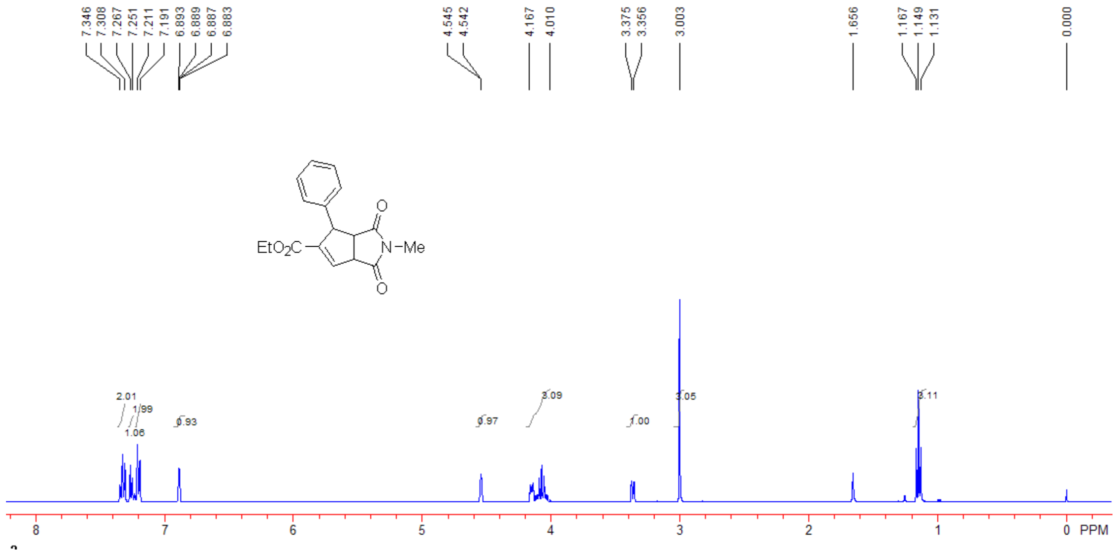

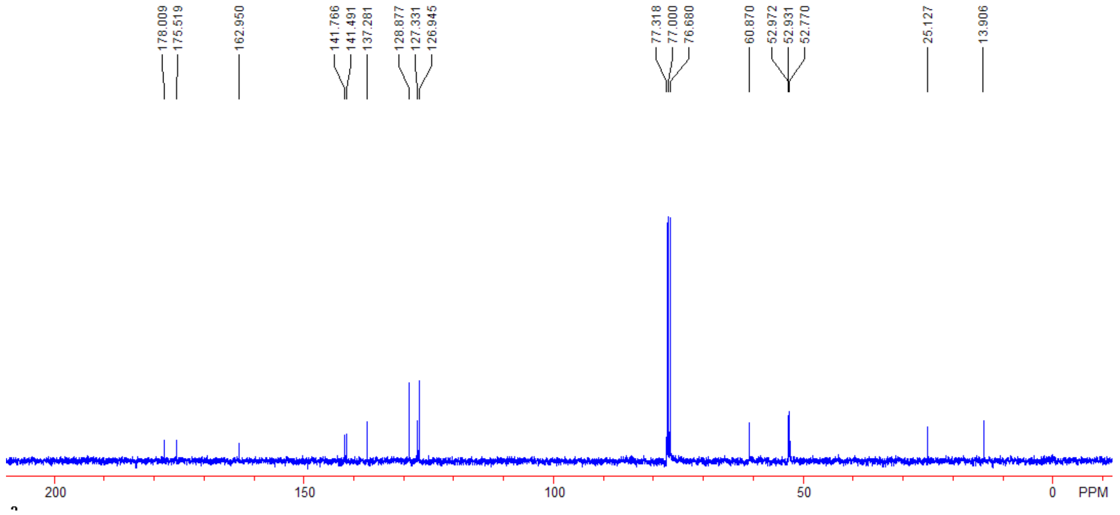

实验时间：2011-09-09, 15:45:23
谱图文件:E:\实验数据\HPLC\dhp\dhp-18-17-N-Me-IC-80-20-0.7-214-rac.org

实验者：
报告时间：2011-11-18, 22:12:30
积分方法:面积归一法

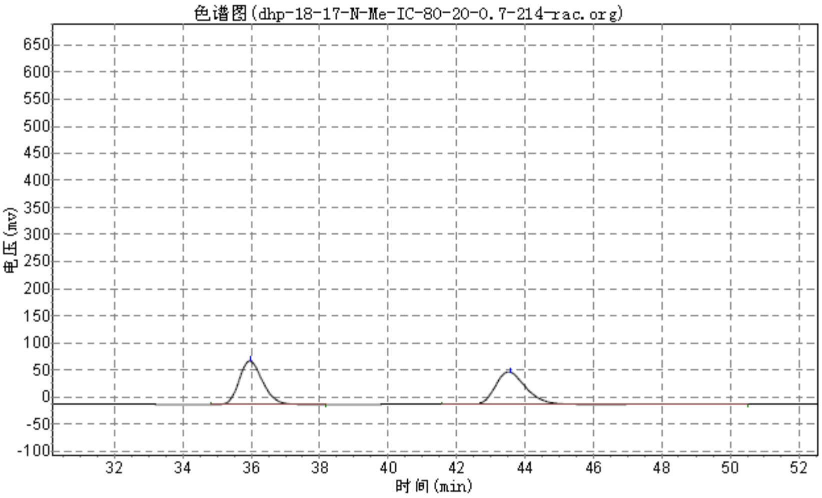

分析结果表

峰号	峰名	保留时间	峰高	峰面积	含量
1		35.965	80659.461	3909325.500	50.2041
2		43.565	60477.879	3877546.500	49.7959
**总计**			141137.340	7786872.000	100.0000

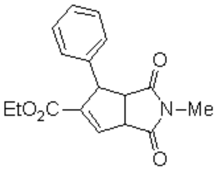

rac

IC-H, 214 nm
Hexane:^{i}PrOH = 80:20
0.7 mL/min

N2000 数据工作站 1

实验时间: 2011-09-09, 16:48:44
谱图文件:E:\实验数据\HPLC\dhp\dhp-18-17-N-Me-IC-80-20-0.7-214-asy.org

实验者:
报告时间: 2011-11-18, 22:13:15
积分方法:面积归一法

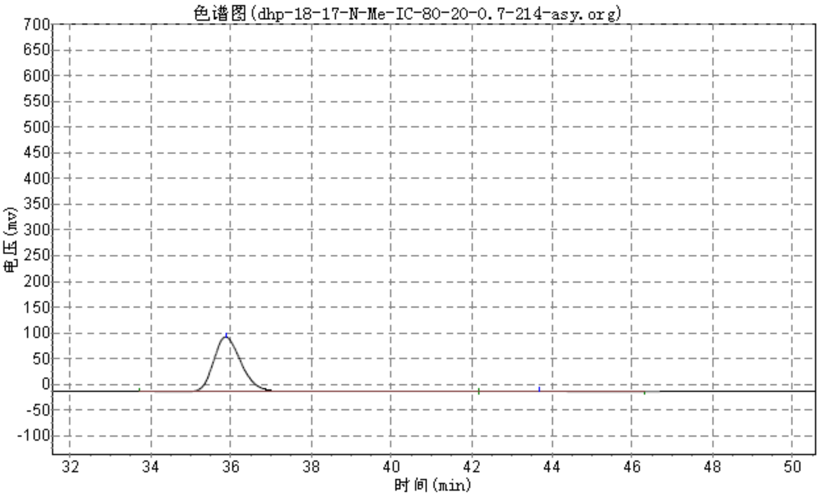

分析结果表

峰号	峰名	保留时间	峰高	峰面积	含量
1		35.873	106873.508	5156261.500	97.2786
2		43.682	2095.773	144247.609	2.7214
**总计**			108969.281	5300509.109	100.0000

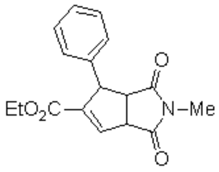

IC-H, 214 nm
Hexane:^{i}PrOH = 80:20
0.7 mL/min

asy

Chiral HPLC report: Enantiomeric excess was determined by HPLC with a Chiralcel IC-H column [λ = 214 nm; eluent: hexane/isopropanol 80/20; flow rate: 0.7 mL/min; t_{major} = 35.87 min, t_{minor} = 43.68 min; ee = 95%; $[\alpha]^{20}_{D}$ = +276.5 (*c* 1.0, $CHCl_3$)].

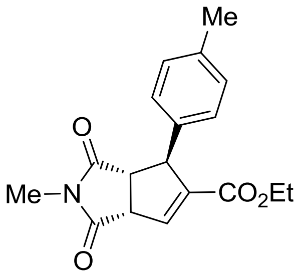

(3a*R*,4*S*,6a*S*)-ethyl 2-methyl-1,3-dioxo-4-(*p*-tolyl)-1,2,3,3a,4,6a-hexahydrocyclopenta[*c*]pyrrole-5-carboxylate (3n). Yield: 20 mg, 64%; colorless oil; IR (CH_2Cl_2): ν 2981, 1780,

1705, 1653, 1634, 1558, 1540, 1513, 1433, 1379, 1327, 1271, 1201, 1126, 1094, 1045, 1000, 959, 819, 786, 739, 721 cm^{-1}; ^{1}H NMR (400 MHz, $CDCl_3$, TMS): δ 1.16 (3H, t, J = 7.2 Hz, CH_3), 2.32 (3H, s, CH_3), 3.00 (3H, s, CH_3), 3.34 (1H, dd, J = 2.0, 7.2 Hz, CH), 4.03–4.15 (3H, m, CH+CH_2), 4.51 (1H, d, J = 2.4 Hz, CH), 6.86–6.87 (1H, m, CH), 7.09 (2H, d, J = 8.0 Hz, ArH), 7.13 (2H, d, J = 8.0 Hz, ArH); ^{13}C NMR (100 MHz, $CDCl_3$, TMS): δ 13.9, 21.0, 25.1, 52.6, 52.89, 52.92, 60.8, 126.8, 129.5, 136.99, 137.03, 138.8, 141.6, 163.0, 175.6, 178.1; MS (ESI) m/z (%): 314.1 (100) [M^+ + 1]; HRMS (MALDI) Calcd. for $C_{18}H_{19}NO_4Na$ (M^+ + Na) requires 336.1206, found: 336.1207; Enantiomeric excess was determined by HPLC with a Chiralcel IC-H column [λ = 214 nm; eluent: hexane/isopropanol 80/20; flow rate: 0.7 mL/min; t_{major} = 33.94 min, t_{minor} = 41.40 min; ee = 96%; $[\alpha]^{20}_{D}$ = +258.2 (c 1.0, $CHCl_3$)].

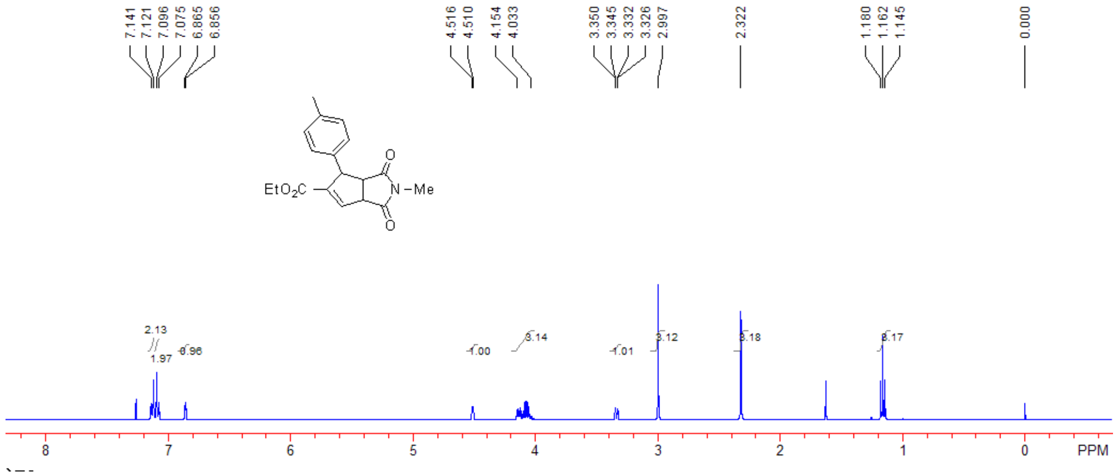

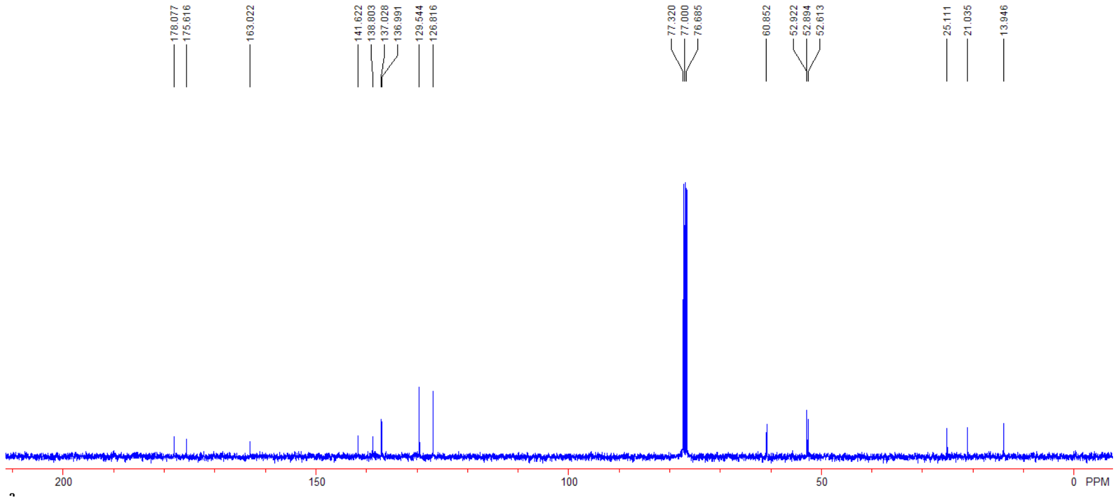

实验时间：2011-09-14,18:46:11
谱图文件:E:\实验数据\HPLC\dhp\dhp-18-21-N-Me-IC-80-20-0.7-214-rac.org

实验者：
报告时间：2011-11-18,22:10:12
积分方法:面积归一法

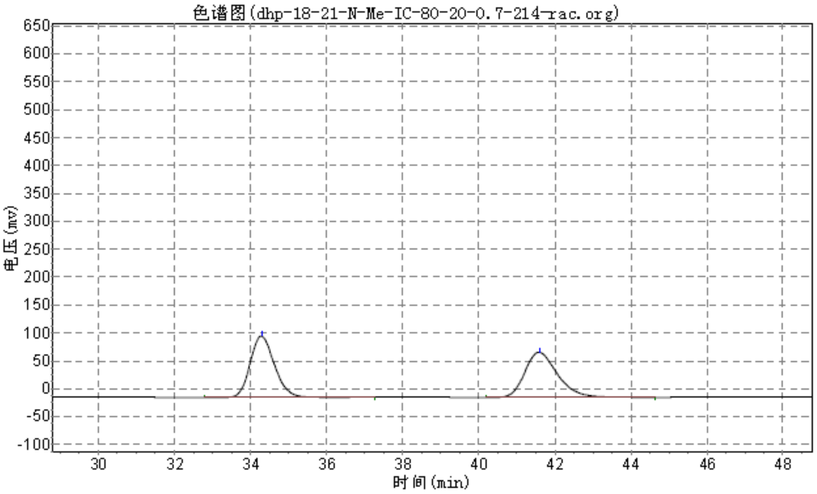

分析结果表

峰号	峰名	保留时间	峰高	峰面积	含量
1		34.290	110073.250	4924989.000	50.3600
2		41.590	80951.609	4854581.000	49.6400
**总计**			191024.859	9779570.000	100.0000

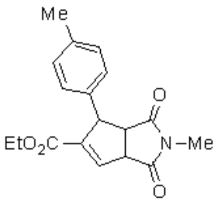

rac

IC-H , 214 nm
Hexane:^{i}PrOH = 80:20
0.7 mL/min

实验时间：2011-09-14,19:43:27
谱图文件:E:\实验数据\HPLC\dhp\dhp-18-21-N-Me-IC-80-20-0.7-214-asy.org

实验者：
报告时间：2011-11-18,22:10:58
积分方法:面积归一法

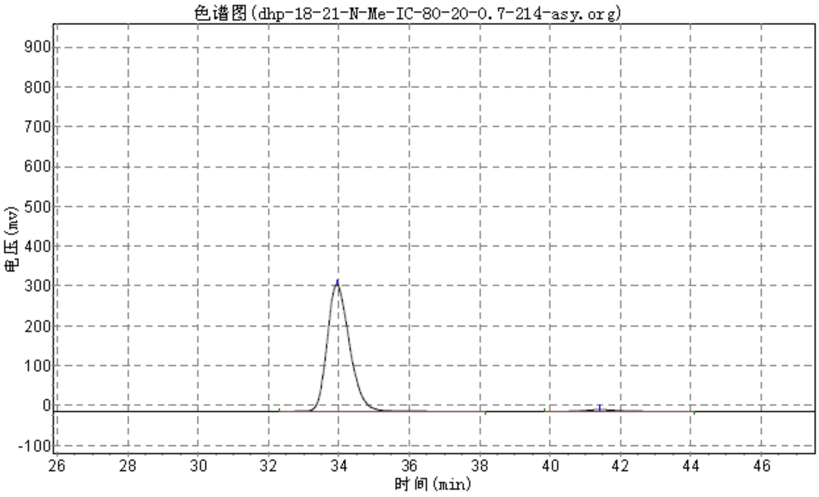

分析结果表

峰号	峰名	保留时间	峰高	峰面积	含量
1		33.947	318548.594	14097616.000	97.9174
2		41.407	4603.955	299846.406	2.0826
**总计**			323152.549	14397462.406	100.0000

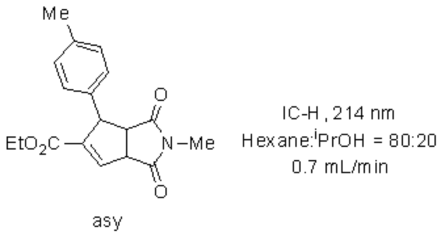

Chiral HPLC report: Enantiomeric excess was determined by HPLC with a Chiralcel IC-H column [λ = 214 nm; eluent: hexane/isopropanol 80/20; flow rate: 0.7 mL/min; t_{major} = 33.94 min, t_{minor} = 41.40 min; ee = 96%; $[\alpha]^{20}_{D}$ = +258.2 (*c* 1.0, $CHCl_3$)].

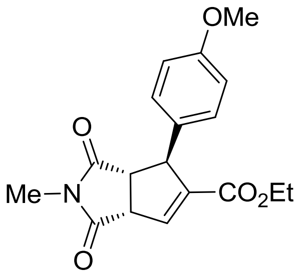

(3a*R*,4*S*,6a*S*)-ethyl 4-(4-methoxyphenyl)-2-methyl-1,3-dioxo-1,2,3,3a,4,6ahexahydrocyclo-penta[*c*]pyrrole-5-carboxylate (3o). Yield: 18 mg, 55%; colorless oil; IR (CH_2Cl_2): ν 2937, 1780, 1704, 1632, 1610, 1512, 1433, 1379, 1274, 1203, 1178, 1126, 1094, 1033, 1000, 959, 833, 794, 727 cm^{-1}; ^1H NMR (400 MHz, $CDCl_3$, TMS): δ 1.16 (3H, t, *J* = 7.2 Hz, CH_3), 3.00 (3H, s, CH_3), 3.33 (1H, dd, *J* = 2.0, 7.2 Hz, CH), 3.79 (3H, s, CH_3), 4.03–4.16 (3H, m, CH+CH_2), 4.49–4.51 (1H, m, CH), 6.84–6.87 (3H, m, CH+ArH), 7.10–7.14 (2H, m, ArH); ^{13}C NMR (100 MHz, $CDCl_3$, TMS): δ 13.9, 25.1, 52.2, 52.8, 52.9, 55.2, 60.8, 114.2, 128.0, 133.9, 136.8, 141.7, 158.7, 163.0, 175.6, 178.1; MS (ESI) *m/z* (%): 330.1 (100) [M^+ + 1]; HRMS (MALDI) Calcd. for $C_{18}H_{19}NO_5Na$ (M^+ + Na) requires 352.1156, found: 352.1145; Enantiomeric excess was determined by HPLC with a Chiralcel IC-H column [λ = 214 nm; eluent: hexane/isopropanol 50/50; flow rate: 0.7 mL/min; t_{major} = 23.56 min, t_{minor} = 34.64 min; ee = 96%; $[\alpha]^{20}_D$ = +273.1 (*c* 1.0, $CHCl_3$)].

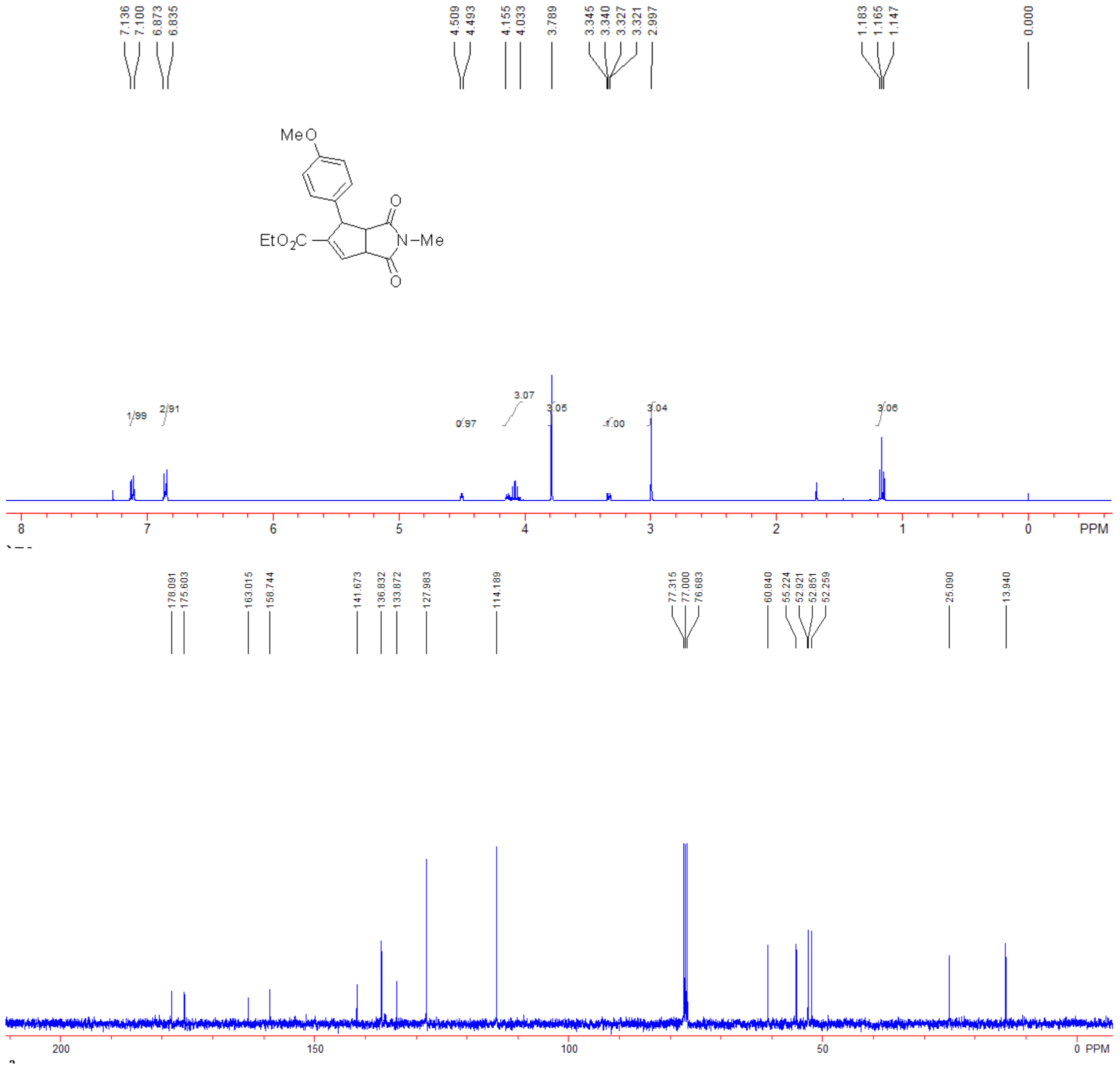

实验时间：2011-09-14，22:47:06
谱图文件:E:\实验数据\HPLC\dhp\dhp-18-22-N-Me-IC-50-50-0.7-214-rac.org

实验者：
报告时间：2011-11-18，22:13:58
积分方法:面积归一法

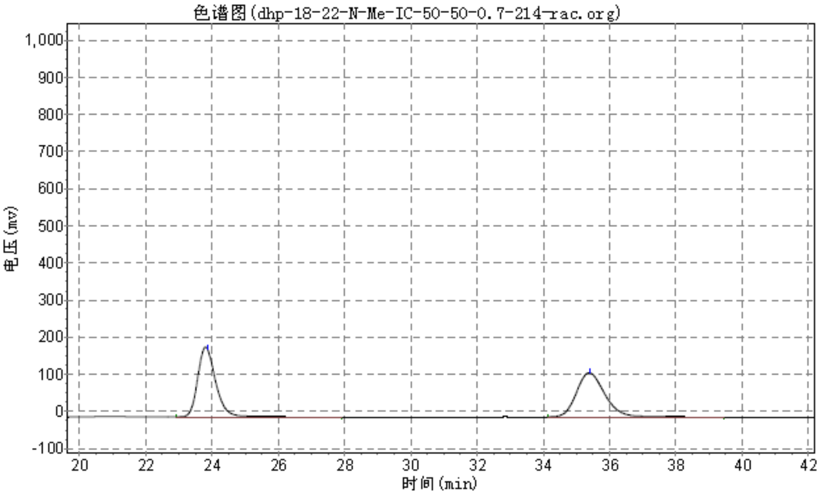

分析结果表

峰号	峰名	保留时间	峰高	峰面积	含量
1		23.857	186342.219	7031739.500	50.0084
2		35.390	118534.805	7029379.500	49.9916
总计			304877.023	14061119.000	100.0000

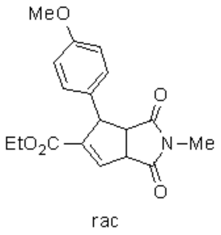

IC-H, 214 nm
Hexane:^{i}PrOH = 50:50
0.7 mL/min

N2000 数据工作站 1

实验时间：2011-09-14, 23:40:34
谱图文件:E:\实验数据\HPLC\dhp\dhp-18-22-N-Me-IC-50-50-0.7-214-asy.org

实验者：
报告时间：2011-11-18, 22:14:52
积分方法:面积归一法

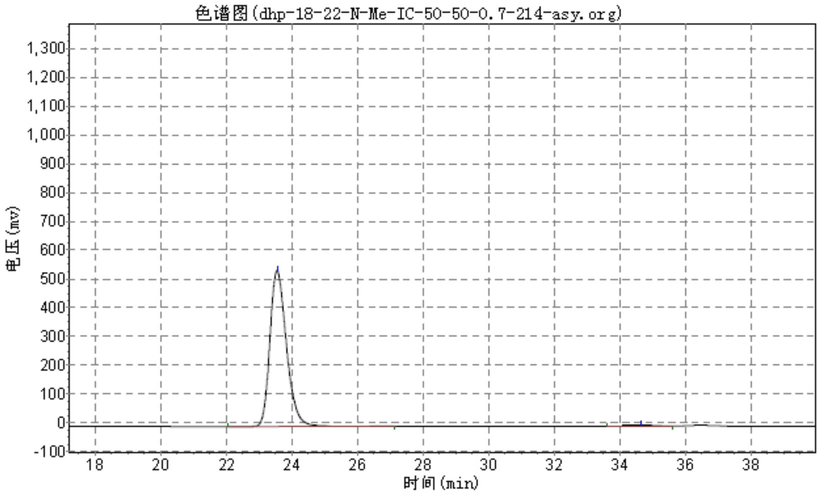

分析结果表

峰号	峰名	保留时间	峰高	峰面积	含量
1		23.555	541739.313	19044796.000	98.7160
2		34.640	4509.533	247716.656	1.2840
**总计**			546248.845	19292512.656	100.0000

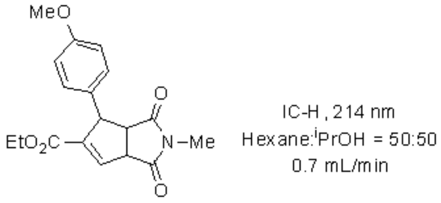

asy

Chiral HPLC report: Enantiomeric excess was determined by HPLC with a Chiralcel IC-H column [λ = 214 nm; eluent: hexane/isopropanol 50/50; flow rate: 0.7 mL/min; t_{major} = 23.56 min, t_{minor} = 34.64 min; ee = 96%; $[\alpha]^{20}_{D}$ = +273.1 (*c* 1.0, $CHCl_3$)].

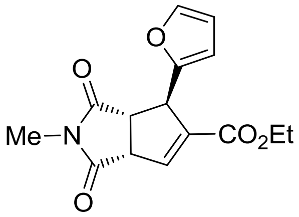

(3a*R*,4*R*,6a*S*)-ethyl 4-(furan-2-yl)-2-methyl-1,3-dioxo-1,2,3,3a,4,6a-hexahydrocyclo-penta[*c*]pyrrole-5-carboxylate (3p). Yield: 26 mg, 69%; yellow oil; IR (CH_2Cl_2): ν 2982, 1780,

1704, 1635, 1505, 1434, 1380, 1282, 1195, 1127, 1096, 1013, 959, 893, 856, 791, 738 cm^{-1}; ^{1}H NMR (400 MHz, $CDCl_3$, TMS): δ 1.23 (3H, t, J = 7.2 Hz, CH_3), 3.00 (3H, s, CH_3), 3.55 (1H, dd, J = 2.0, 7.2 Hz, CH), 4.04–4.15 (3H, m, CH+CH_2), 4.60 (1H, d, J = 2.0 Hz, CH), 6.18 (1H, d, J = 3.2 Hz, ArH), 6.32 (1H, dd, J = 2.0, 3.2 Hz, CH), 6.83–6.84 (1H, m, ArH), 7.31–7.32 (1H, m, ArH); ^{13}C NMR (100 MHz, $CDCl_3$, TMS): δ 14.0, 25.2, 46.2, 49.7, 52.9, 61.0, 106.5, 110.6, 138.1, 138.8, 142.0, 153.2, 162.8, 175.5, 177.7; MS (ESI) m/z (%): 290.0 (100) [M^+ + 1]; HRMS (ESI) Calcd. for $C_{15}H_{15}NNaO_5$ (M^+ + Na) requires 312.0842, found: 312.0836; Enantiomeric excess was determined by HPLC with a Chiralcel IC-H column [λ = 214 nm; eluent: hexane/isopropanol 50/50; flow rate: 0.7 mL/min; t_{major} = 19.80 min, t_{minor} = 23.53 min; ee = 96%; $[\alpha]^{20}_{D}$ = +240.8 (c 1.0, $CHCl_3$)].

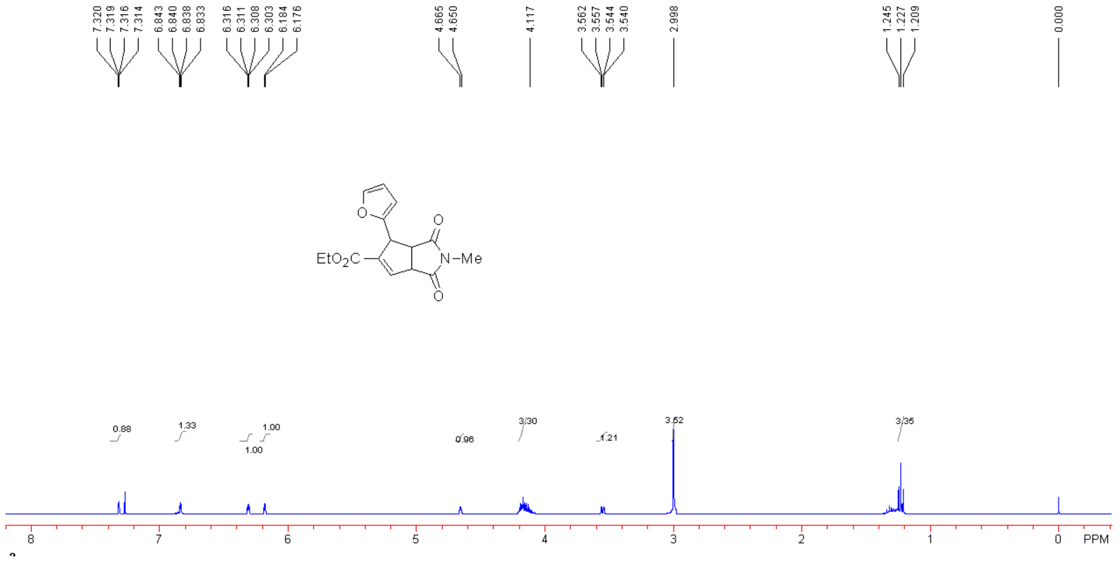

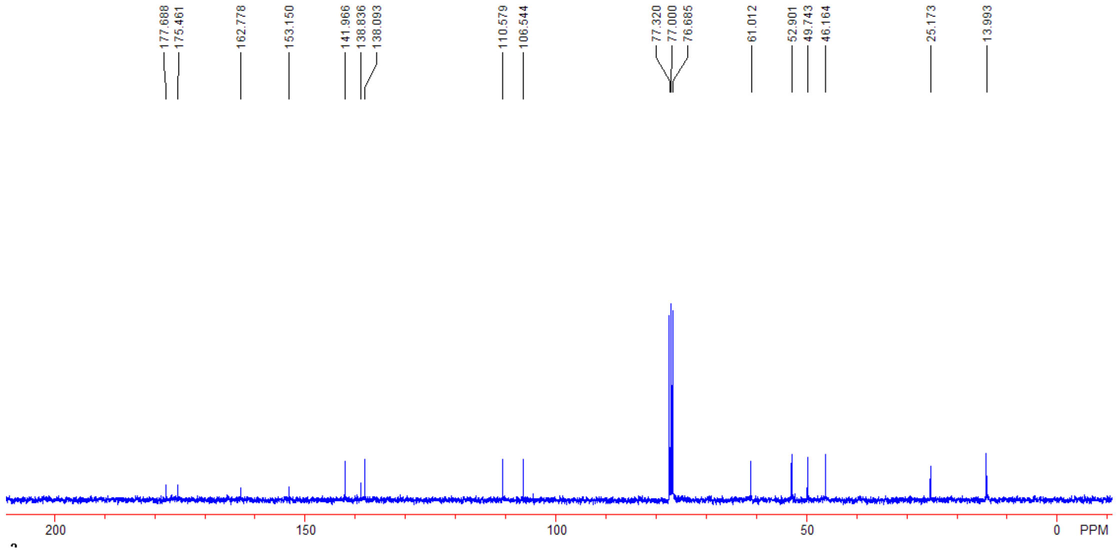

实验时间: 2011-10-21,10:43:14
谱图文件:E:\实验数据\HPLC\dhp\dhp-18-44-IC-50-50-0.7-214-rac.org

实验者:
报告时间: 2011-11-26,10:54:42
积分方法:面积归一法

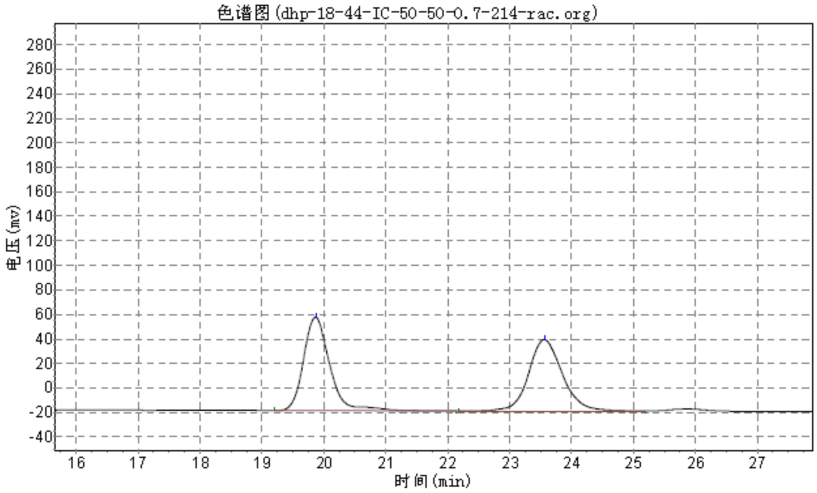

分析结果表

峰号	峰名	保留时间	峰高	峰面积	含量
1		19.865	75889.680	2232837.000	50.1217
2		23.565	58237.262	2221996.250	49.8783
**总计**			134126.941	4454833.250	100.0000

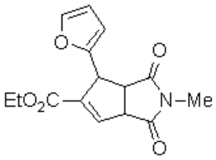

rac

IC-H, 214 nm
Hexane:^{i}PrOH = 50:50
0.7 mL/min

N2000 数据工作站 1

实验时间：2011-10-21,11:24:11
谱图文件:E:\实验数据\HPLC\dhp\dhp-18-44-IC-50-50-0.7-214-asy.org

实验者：
报告时间：2011-11-26,10:56:55
积分方法:面积归一法

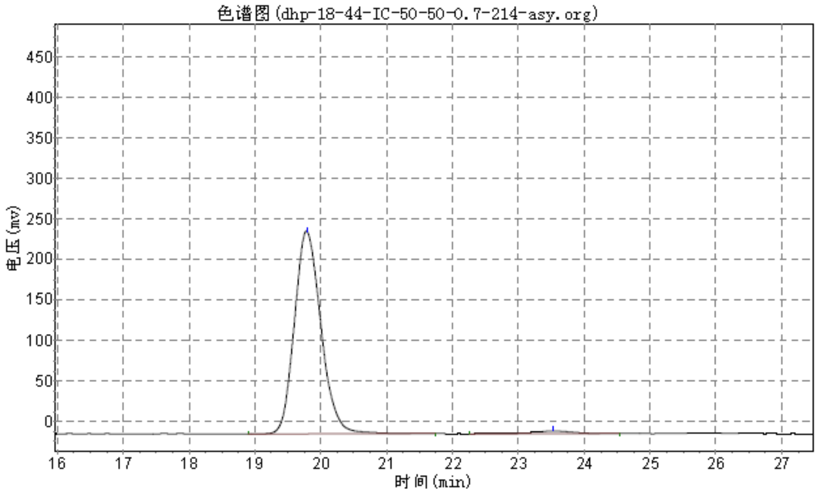

分析结果表

峰号	峰名	保留时间	峰高	峰面积	含量
1		19.798	250046.594	7008134.500	97.8119
2		23.532	3327.882	156772.406	2.1881
**总计**			253374.476	7164906.906	100.0000

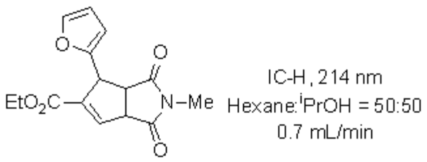

asy

Chiral HPLC report: Enantiomeric excess was determined by HPLC with a Chiralcel IC-H column [λ = 214 nm; eluent: hexane/isopropanol 50/50; flow rate: 0.7 mL/min; t_{major} = 19.80 min, t_{minor} = 23.53 min; ee = 96%; $[\alpha]^{20}_{D}$ = +240.8 (*c* 1.0, $CHCl_3$)].

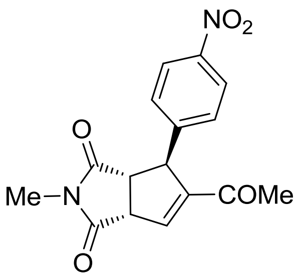

(3a*S*,6*S*,6a*R*)-5-acetyl-2-methyl-6-(4-nitrophenyl)-6,6a-dihydrocyclopenta[*c*]pyrrole-1,3(2*H*,3*H*)-dione (3q). Yield: 32 mg, >99%; colorless solid, mp 268–272 ^{o}C; IR (CH_2Cl_2): ν 2957,

2927, 2858, 1780, 1701, 1668, 1596, 1514, 1433, 1347, 1280, 1124, 1058, 1011, 963, 851, 809, 756, 740, 705, 640 cm^{-1}; ^{1}H NMR (400 MHz, $CDCl_3$, TMS): δ 2.32 (3H, s, CH_3), 3.03 (3H, s, CH_3) 3.33 (1H, dd, J = 2.0, 7.2 Hz, CH), 4.28–4.31 (1H, m, CH), 4.65–4.68 (1H, m, CH), 6.89 (1H, dd, J = 1.2, 2.0 Hz, CH), 7.36 (2H, d, J = 8.4 Hz, ArH), 8.18 (2H, d, J = 8.4 Hz, ArH); ^{13}C NMR (100 MHz, $CDCl_3$, TMS): δ 25.3, 27.2, 51.7, 52.3, 53.5, 124.2, 127.9, 138.3, 147.1, 148.3, 149.1, 174.8, 177.1, 194.1; MS (ESI) m/z (%): 315.0 (100) [M^+ + 1]; HRMS (ESI) Calcd. for $C_{16}H_{14}N_2NaO_5$ (M^+ + Na) requires 337.0795, found: 337.0793; Enantiomeric excess was determined by HPLC with a Chiralcel PA-2 column [λ = 214 nm; eluent: hexane/isopropanol 50/50; flow rate: 0.7 mL/min; t_{major} = 47.66 min, t_{minor} = 70.64 min; ee = 97%; $[\alpha]^{20}_{D}$ = +389.8 (c 1.0, $CHCl_3$)].

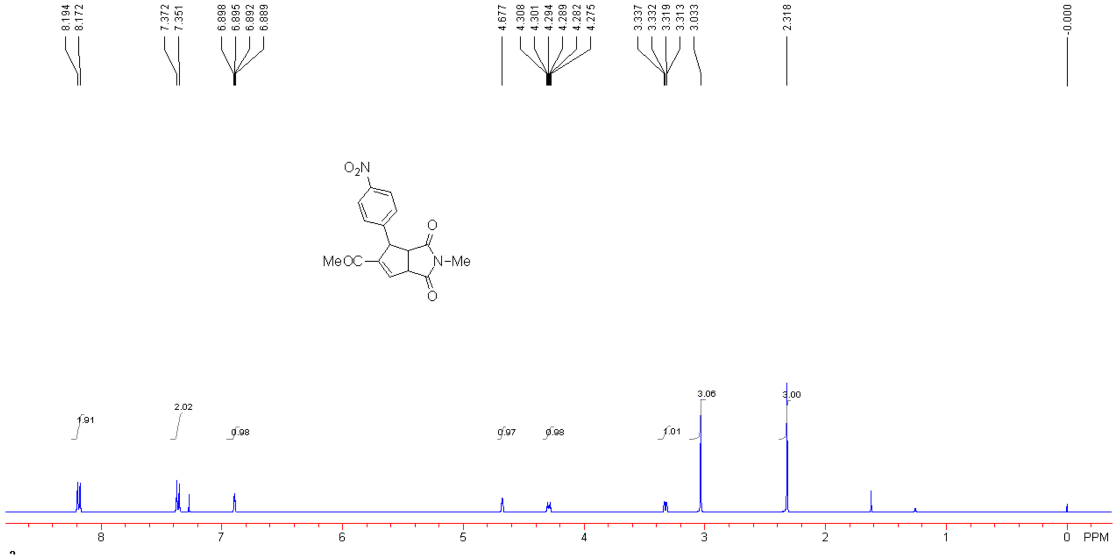

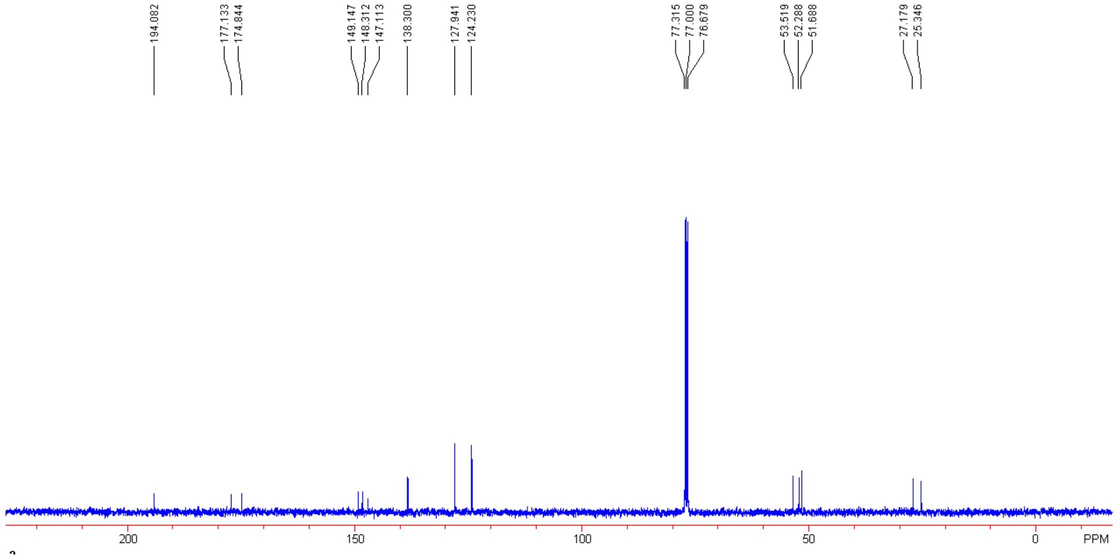

实验时间：2012-01-14,12:07:09
谱图文件:E:\实验数据\HPLC\dhp\dhp-19-70-sub1-PA-2-50-50-0.7-214-rac-re.org
实验者：
报告时间：2012-01-16,15:44:44
积分方法:面积归一法

使用仪器类型:气相色谱 检测器:FID 进样器:分流

柱温:程序升温

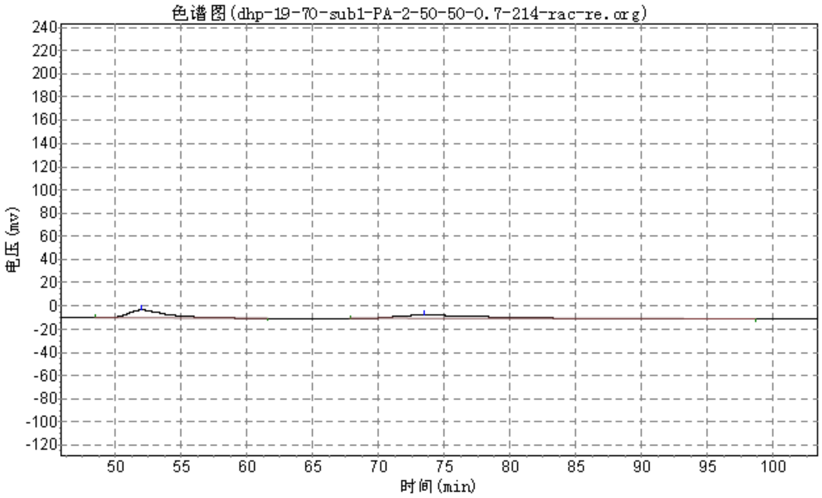

分析结果表

峰号	峰名	保留时间	峰高	峰面积	含量
1		51.985	6634.631	1377864.750	49.0344
2		73.472	2890.285	1432131.500	50.9656
**总计**			9524.916	2809996.250	100.0000

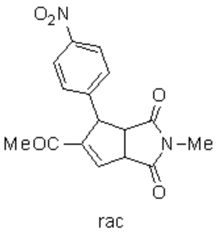

PA-2, 214 nm
Hexane:^{i}PrOH = 50:50
0.7 mL/min

实验时间：2012-01-14,14:28:13
谱图文件:E:\实验数据\HPLC\dhp\dhp-19-70-sub1-PA-2-50-50-0.7-214-asy.org

实验者：
报告时间：2012-01-16,15:40:53
积分方法:面积归一法

使用仪器类型:气相色谱 检测器:FID 进样器:分流

柱温:程序升温

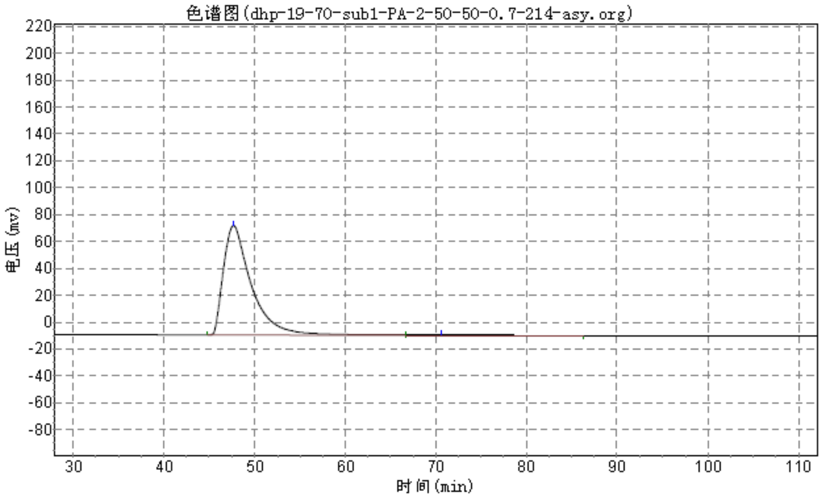

分析结果表

峰号	峰名	保留时间	峰高	峰面积	含量
1		47.662	81543.414	17458990.000	98.3125
2		70.643	676.247	299673.844	1.6875
总计			82219.661	17758663.844	100.0000

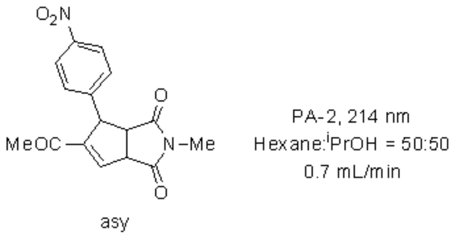

Chiral HPLC report: Enantiomeric excess was determined by HPLC with a Chiralcel PA-2 column [λ = 214 nm; eluent: hexane/isopropanol 50/50; flow rate: 0.7 mL/min; t_{major} = 47.66 min, t_{minor} = 70.64 min; ee = 97%; $[\alpha]^{20}_{D}$ = +389.8 (c 1.0, $CHCl_3$)].

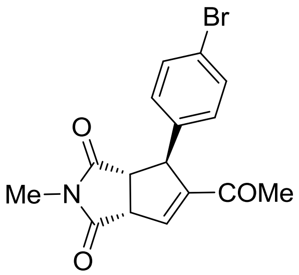

(3a*S*,6*S*,6a*R*)-5-acetyl-6-(4-bromophenyl)-2-methyl-6,6a-dihydrocyclopenta[*c*]pyrrole-1,3(2*H*,3a*H*)-dione (3o). Yield: 32 mg, 92%; colorless solid, mp 185–189 °C; IR (CH_2Cl_2): ν 3072, 2950, 2926, 1780, 1701, 1677, 1616, 1487, 1432, 1380, 1282, 1127, 1072, 1010, 962, 893, 825, 744 cm^{-1}; ^{1}H NMR (400 MHz, $CDCl_3$, TMS): δ 2.28 (3H, s, CH_3), 3.01 (3H, s, CH_3) 3.29 (1H, dd, *J* = 1.6, 7.2 Hz, CH), 4.20–4.23 (1H, m, CH), 4.53–4.56 (1H, m, CH), 6.89 (1H, dd, *J* = 1.6, 2.8 Hz, CH), 7.06 (2H, d, *J* = 8.4 Hz, ArH), 7.44 (2H, d, *J* = 8.4 Hz, ArH); ^{13}C NMR (100 MHz, $CDCl_3$, TMS): δ 25.2, 27.3, 52.0, 52.1, 53.4, 121.2, 128.6, 132.0, 137.4, 140.8, 148.7, 175.3, 177.5, 194.3; MS (ESI) *m/z* (%): 348.0 (100) [M$^+$ + 1]; HRMS (ESI) Calcd. for $C_{16}H_{14}BrNNaO_3$ (M$^+$ + Na) requires 370.0049, found: 370.0037; Enantiomeric excess was determined by HPLC with a Chiralcel PA-2 column [λ = 214 nm; eluent: hexane/isopropanol 50/50; flow rate: 0.7 mL/min; t_{major} = 17.12 min, t_{minor} = 24.92 min; ee = 98%; $[\alpha]^{20}_D$ = +360.6 (*c* 1.0, $CHCl_3$)].

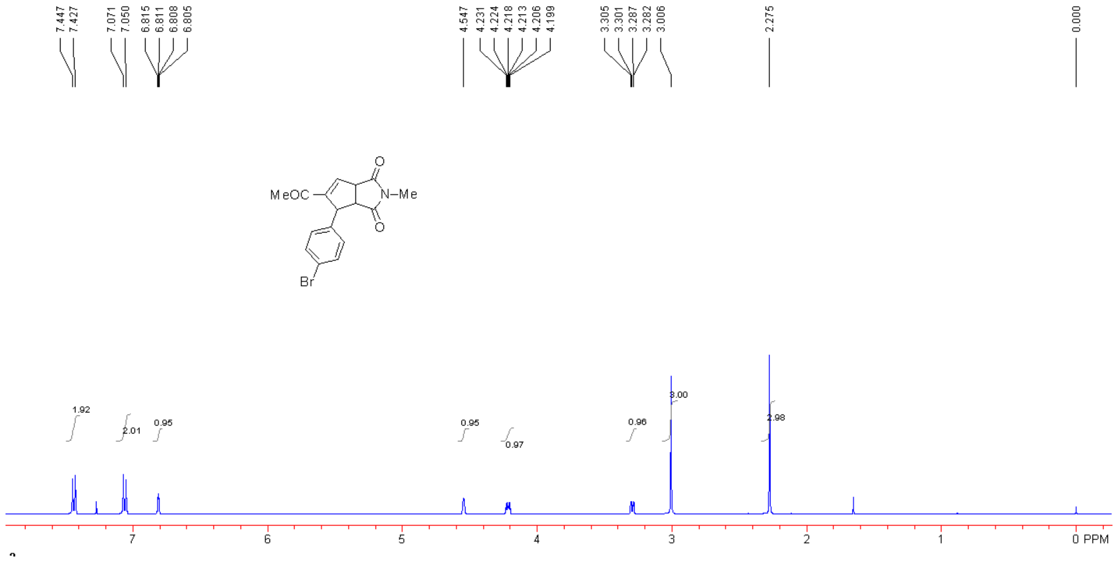

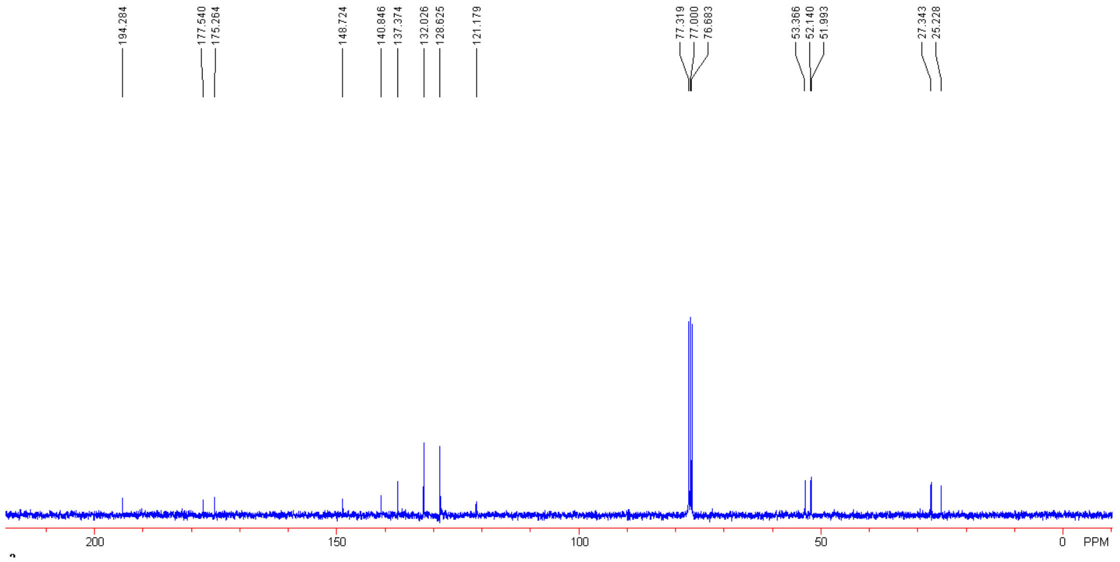

HPLC Report

Sample Name: Data File:Dhp-19-70-sub2-rac PA-2 55 214 0.7.che

Operator: Date:2012-01-13

Time:15:35

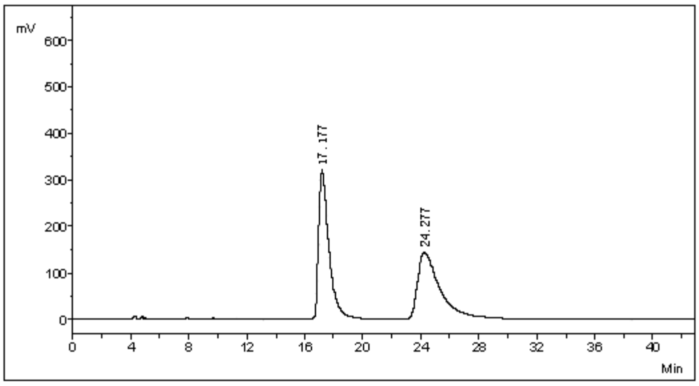

No.	PeakNo	ID.Name	R.Time	PeakHeight	PeakArea	PerCent
1	1		17.177	320400.0	16081475.9	50.8485
2	2		24.277	143764.6	15544750.1	49.1515
Total				464164.6	31626226.0	100.0000

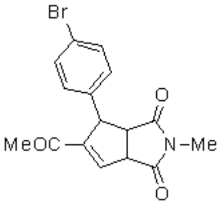

rac

PA-2, 214 nm
Hexane:^{i}PrOH = 50:50
0.7 mL/min

HPLC Report

Sample Name: Data File:Dhp-19-70-sub2-asy PA-2 55 214 0.7.che

Operator: Date:2012-01-13

Time:16:20

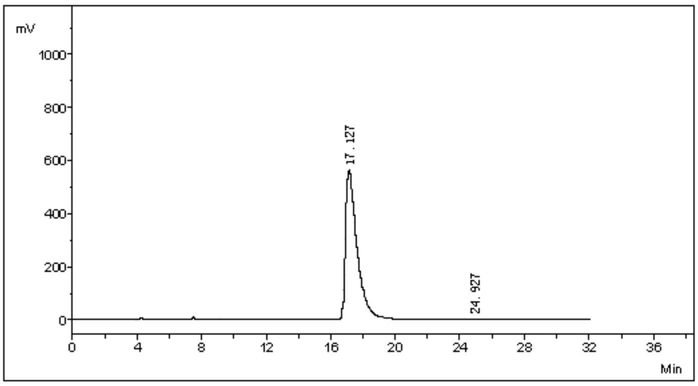

No.	PeakNo	ID.Name	R.Time	PeakHeight	PeakArea	PerCent
1	1		17.127	562022.2	28294438.9	99.1938
2	2		24.927	2200.4	229976.4	0.8062
Total				564222.6	28524415.3	100.0000

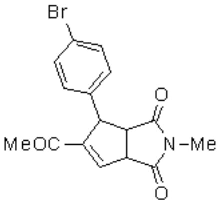

asy

PA-2, 214 nm
Hexane:^{i}PrOH = 50:50
0.7 mL/min

Chiral HPLC report: Enantiomeric excess was determined by HPLC with a Chiralcel PA-2 column [λ = 214 nm; eluent: hexane/isopropanol 50/50; flow rate: 0.7 mL/min; t_{major} = 17.12 min, t_{minor} = 24.92 min; ee = 98%; $[\alpha]^{20}_{D}$ = +360.6 (*c* 1.0, $CHCl_3$)].

2. General procedure for the dihydroxylation of 4c [2,3].

General Procedure: $NaIO_4$ (0.16 mmol, 34 mg, 2 equiv) and distilled water (0.1 mL) were added to a flask. After the $NaIO_4$ had been dissolved, the solution was cooled to 0 ^oC, and two drops of H_2SO_4 aqueous (2 M) was added and stirred for 5 min. Then $RuCl_3{\cdot}3H_2O$ (0.008 mmol, 3.0 mg, 0.1 equiv) was added, followed by two drops of H_2SO_4 aqueous (2 M). The resulting solution was stirred for 5 min, and then EtOAc (0.2 mL) was added. After the solution had been stirred for 5 min, CH_3CN (0.3 mL) was added. The solution was stirred for 5 min, and then a solution of substrate **3c** (0.08 mmol,

34 mg) in EtOAc (0.3 mL) was added. The solution was further stirred for 10 min, and then 10% $NaHCO_3$ (0.7 mL) and saturated Na_2SO_3 solution (2.5 mL) were added. After the solution had been stirred for 30 min at room temperature, it was extracted with EtOAc (3 × 5 mL). The combined organic extracts were dried over anhydrous Na_2SO_4, filtered, and the solvent was removed under reduced pressure and residue was chromatographed on silica gel (elution with petroleum ether/EtOAc 2:1) to provide compound **4c**.

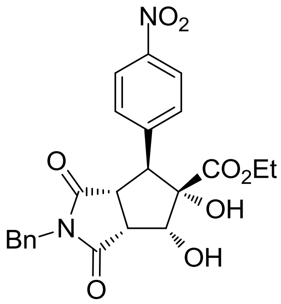

(3a*R*,4*R*,5*S*,6*S*,6a*R*)-ethyl 2-benzyl-4,5-dihydroxy-6-(4-nitrophenyl)-1,3-dioxooctahydrocyclo-penta[*c*]pyrrole-5-carboxylate (4c). Yield: 25 mg, 69%; yellow oil; IR (CH_2Cl_2): ν 2961, 2925, 2854, 1699, 1519, 1399, 1347, 1259, 1089, 1017, 796, 699 cm^{-1}; ^1H NMR (400 MHz, $CDCl_3$, TMS): δ 1.00 (3H, t, J = 7.2 Hz, CH_3), 3.32 (1H, d, J = 3.6 Hz, OH), 3.66–3.76 (2H, m, CH+CH), 3.82 (1H, s, OH), 3.88 (1H, d, J = 5.6 Hz, CH), 3.92–4.08 (2H, m, CH_2), 4.59 (1H, dd, J = 3.6, 5.6 Hz, CH), 4.63–4.74 (2H, m, CH_2), 7.28–7.41 (5H, m, ArH), 7.48–7.51 (2H, m, ArH), 8.18–8.20 (2H, m, ArH); ^{13}C NMR (100 MHz, $CDCl_3$, TMS): δ 13.8, 42.8, 46.8, 47.6, 52.9, 62.9, 74.7, 87.3, 123.7, 127.9, 128.5, 128.58, 128.64, 135.2, 144.3, 147.3, 170.7, 175.0, 177.3; MS (MADLI) *m/z* (%): 477.4 (38.0) [M^+ + Na]; HRMS (MADLI) Calcd. for $C_{23}H_{23}N_2O_8$ (M^+ + 1) requires 455.1449, found: 455.1457; $[\alpha]^{20}_D$ = +73.3 (*c* 1.0, $CHCl_3$)].

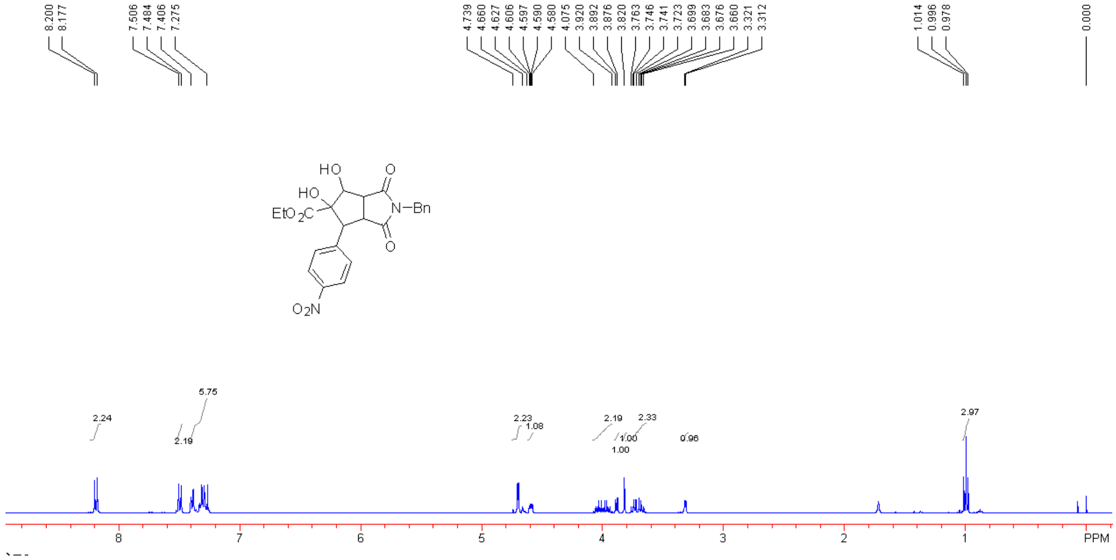

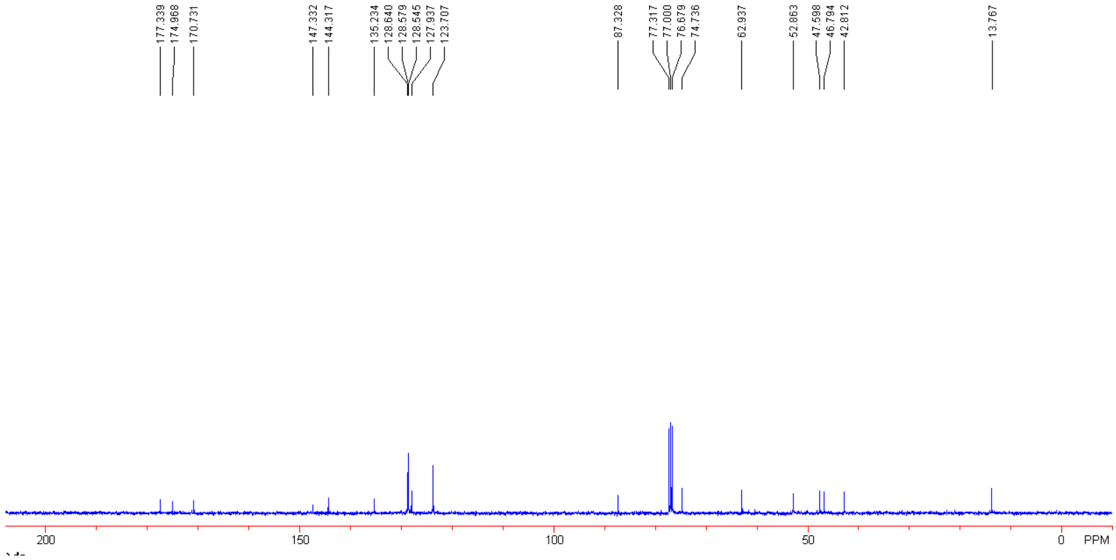

^{1}H NMR (400 MHz) spectroscopy of **4c** after adding D_2O into the NMR tube.

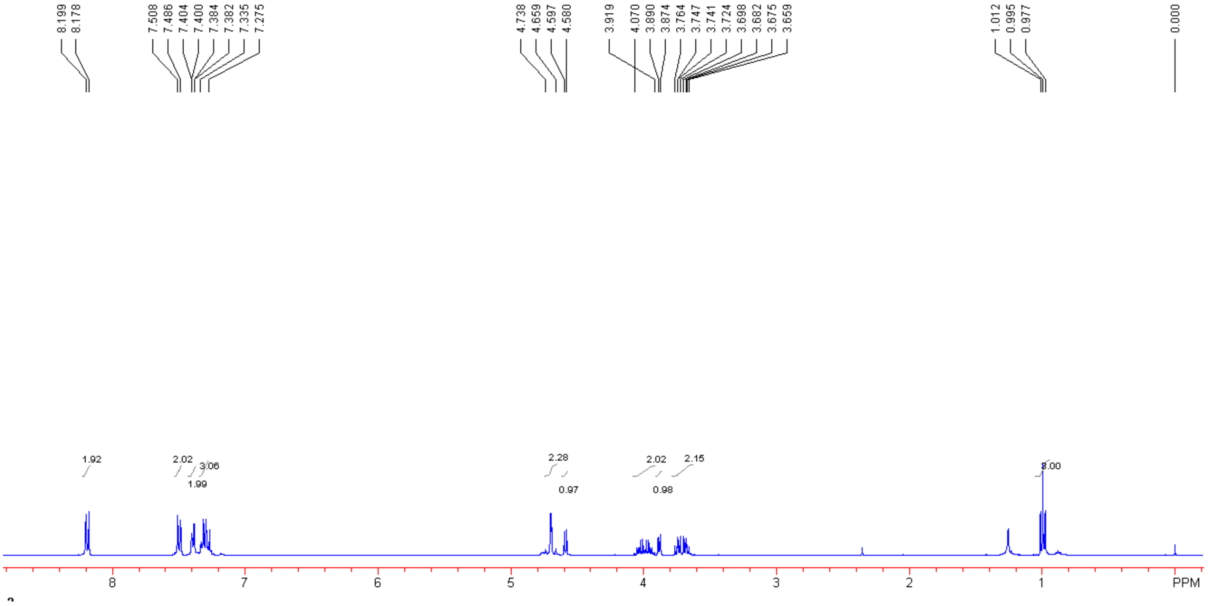

NOESY (Varian Mercury-300 MHz)

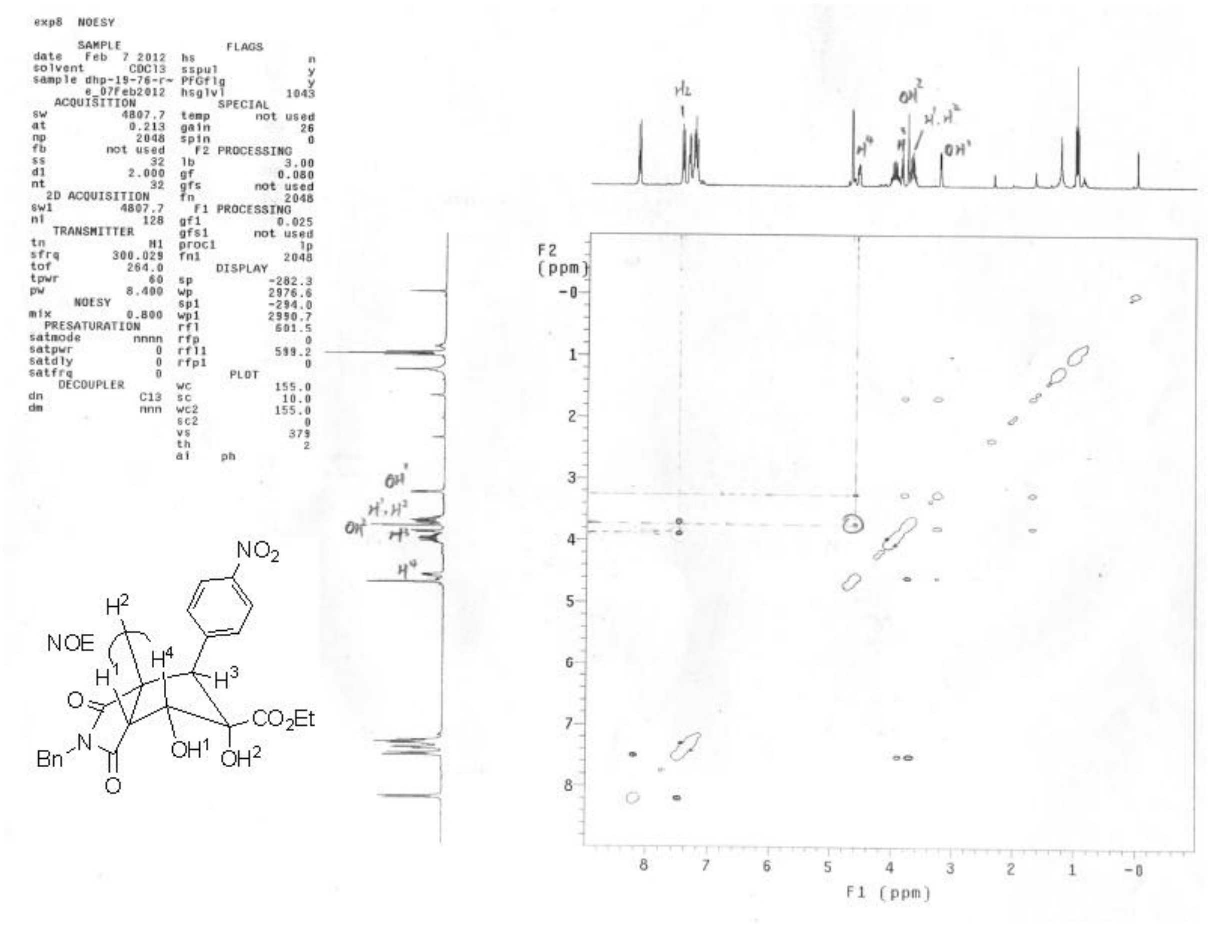

NOESY spectra of **4c** after adding D_2O into the NMR tube

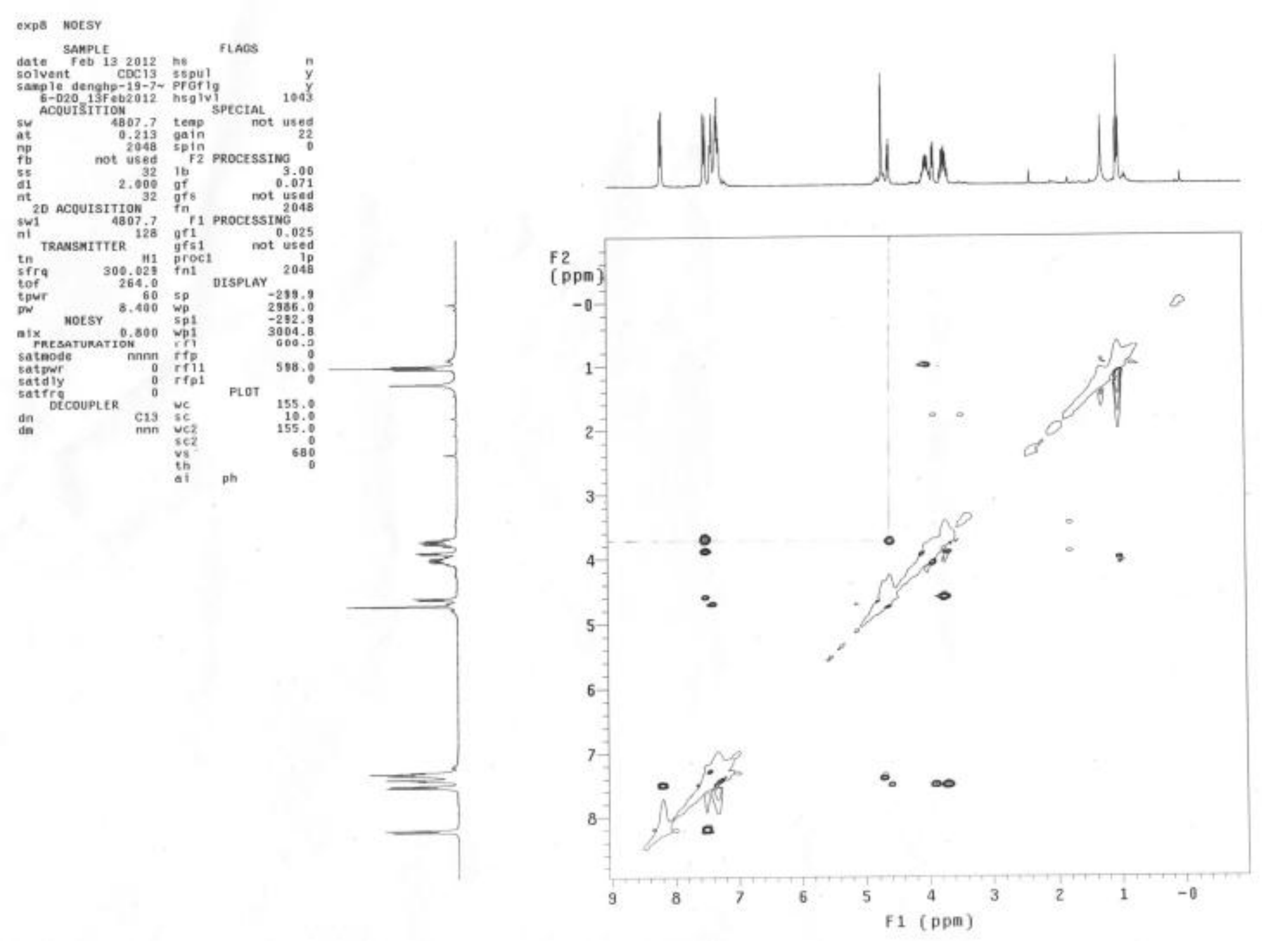

3. ^{31}P NMR Spectroscopy of the control experiments

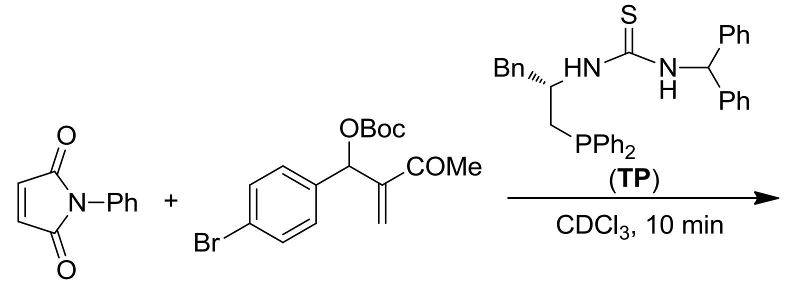

1a **2d**

a. **TP** (0.05 mmol)
b. **1a** (0.05 mmol) and **TP** (0.05 mmol)
c. **2d** (0.05 mmol) and **TP** (0.05 mmol)
d. **1a** (0.05 mmol), **2d** (0.05 mmol) and **TP** (0.05 mmol)

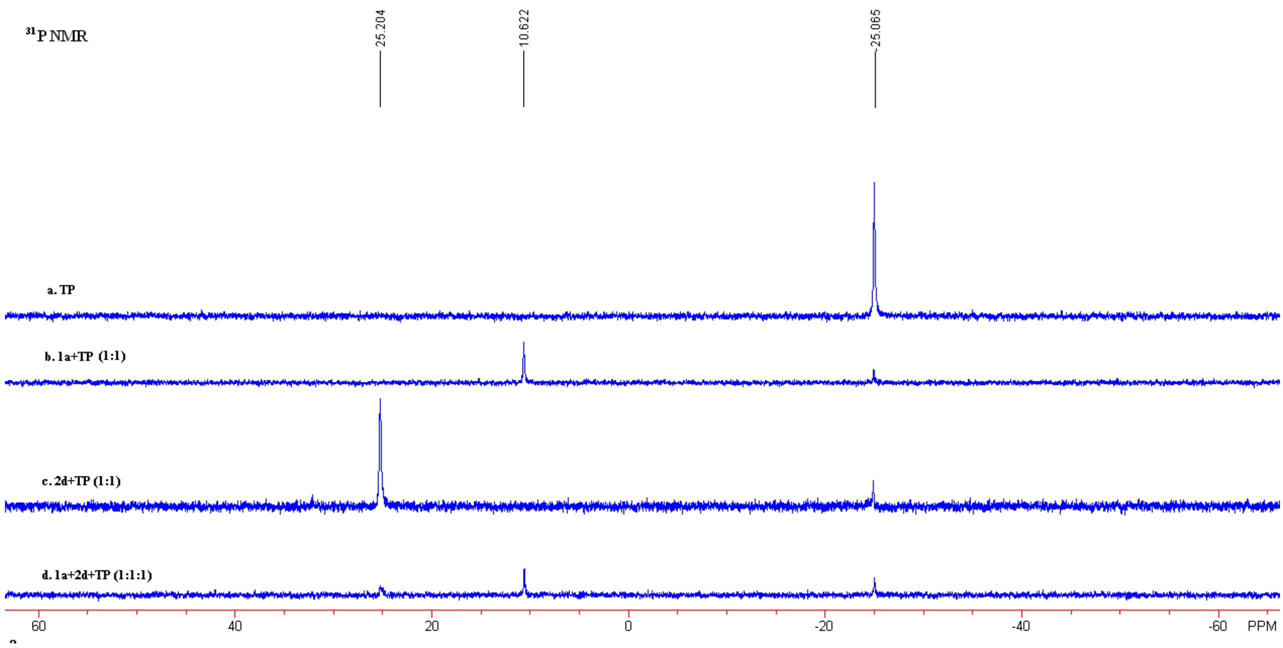

Figure SI-1. ^{31}P NMR Spectra (161.9 MHz, $CDCl_3$) of control experiments

4. References:

1. Deng, H.-P.; Wei, Y.; Shi, M. *Adv. Synth. Catal.* **2012**, *354*, 783–789.
2. Plietker, B.; Niggemann, M. *Org. Lett.* **2003**, *5*, 3353–3356.
3. Wurz, R. P.; Fu, G. C. *J. Am. Chem. Soc.* **2005**, *127*, 12234–12235.
