# Supplementary material for: Chiral multifunctional thiourea-phosphine catalyzed asymmetric [3 + 2] annulation of Morita–Baylis–Hillman carbonates with maleimides
Source: Beilstein J Org Chem. 2012 Jul 16;8:1098–104. doi: 10.3762/bjoc.8.121 (PMC3458726; doi:10.3762/bjoc.8.121)
Supplement: File 2 — Crystal structure data of compound 3r. [file Beilstein_J_Org_Chem-08-1098-s002.pdf]

Supporting Information

for

Chiral multifunctional thiourea-phosphine catalyzed asymmetric [3 + 2] annulation of Morita–Baylis–Hillman carbonates with maleimides

Hong-Ping Deng[1], De Wang[2], Yin Wei[1] and Min Shi*[1,2,§]

Address: [1]State Key Laboratory of Organometallic Chemistry, Shanghai Institute of Organic Chemistry, Chinese Academy of Sciences, 354 Fenglin Road, Shanghai 200032, People's Republic of China and [2]Key Laboratory for Advanced Materials and Institute of Fine Chemicals, School of Chemistry & Molecular Engineering, East China University of Science and Technology, and 130 MeiLong Road, Shanghai 200237, People's Republic of China

Email: Min Shi - Mshi@mail.sioc.ac.cn

[§]Fax: 86-21-64166128

*Corresponding author

Crystal structure data of compound 3r

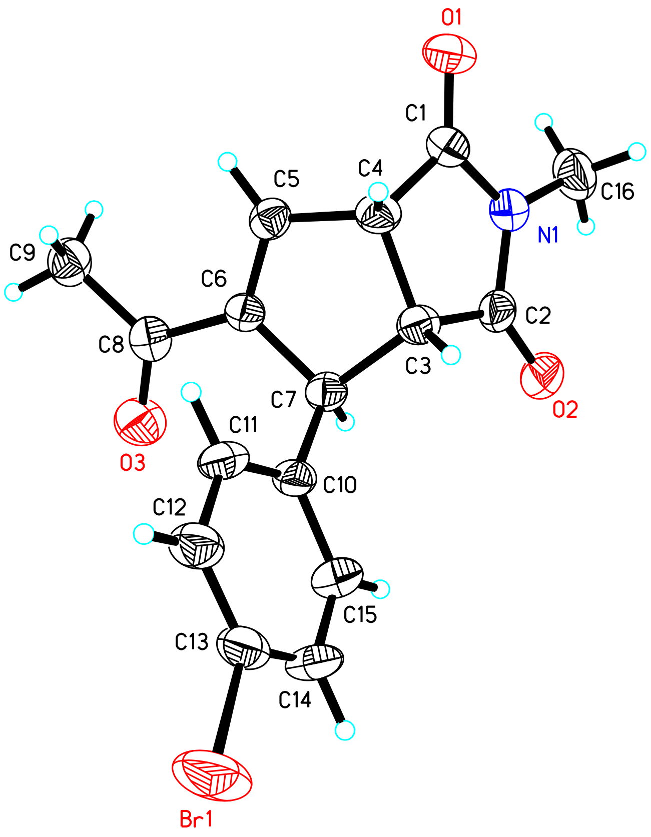

The crystal data of **3r** have been deposited in CCDC with number 863228. Empirical Formula: $C_{16}H_{14}BrNO_3$; Formula Weight: 348.19; Crystal Color, Habit: colorless, Crystal Dimensions: 0.211 × 0.157 × 0.104 mm; Crystal System: Orthorhombic; Lattice Parameters: a = 9.5280(6)Å, b = 9.6271(6) Å, c = 16.3779(10) Å, α = 90°, β = 90°, γ = 90°, V = 1502.30(16) $Å^3$; Space group: P2(1)2(1)2(1); Z = 4; D_{calc}= 1.539 g/cm^3; F_{000} = 704; Final R indices [I > 2sigma(I)]: R1 = 0.0384; wR2 = 0.0900.
